# Organocatalytic Asymmetric Conjugate Addition of Aldehydes to Maleimides and Nitroalkenes in Deep Eutectic Solvents

**DOI:** 10.3390/molecules24224058

**Published:** 2019-11-09

**Authors:** Alejandro Torregrosa-Chinillach, Alba Sánchez-Laó, Elisa Santagostino, Rafael Chinchilla

**Affiliations:** Departamento de Química Orgánica, Facultad de Ciencias, and Instituto de Síntesis Orgánica (ISO), Universidad de Alicante, Apdo. 99, 03080 Alicante, Spain; alextorregrosa96@gmail.com (A.T.-C.); alba.sl96@hotmail.com (A.S.-L.); elisanta33@gmail.com (E.S.)

**Keywords:** organocatalysis, deep eutectic solvents, conjugate addition, maleimides, nitroalkenes

## Abstract

A chiral primary amine-salicylamide is used as an organocatalyst for the enantioselective conjugate addition of α,α-disubstituted aldehydes to maleimides and nitroalkenes. The reactions are performed in deep eutectic solvents as reaction media at room temperature, leading to the corresponding adducts with enantioselectivities up to 88% (for maleimides) and 80% (for nitroalkenes). Catalyst and solvent can be recovered and reused.

## 1. Introduction

Currently, traditional volatile organic compounds (VOCs) are the common solvents for performing organic reactions although, from an environmental point of view, they show many intrinsic drawbacks, such as accumulation in the atmosphere due to their low boiling points, flammability, toxicity and non-biodegradability. For all these reasons several greener and friendlier synthetic methodologies based on alternative reaction media have been developed, all of them having certain number of advantages, as well as disadvantages [1,2].

Among these alternative reaction media, deep eutectic solvents (DESs) are attracting a growing interest [3]. A DES is a combination of two or three compounds, Lewis or Brønsted acids and bases containing a variety of anionic and/or cationic species, which interacts through hydrogen bonds forming a eutectic mixture with a melting point lower than the individual components [3]. DESs are non-volatile, show a low ecological footprint, are economical, essentially nontoxic and easily recyclable, therefore research concerning their use as environmentally friendly neoteric solvents in organic synthesis is growing rapidly [4,5,6,7,8,9,10,11].

In addition, probably the most attractive methodology for the enantioselective preparation of functionalized molecules in organic synthesis is the use of asymmetric organocatalysis, since metal-free small organic compounds are used as catalysts under usually very mild and simple reaction conditions [12,13]. This methodology maintains sustainability in organic synthesis and provides many advantages, such as accessibility, inexpensive catalysts and reduced toxicity. However, most of these asymmetric processes are carried out using environmentally unfriendly VOCs as reaction media, although efforts have been devoted to achieving more sustainable synthetic procedures [14].

**Figure 1:**
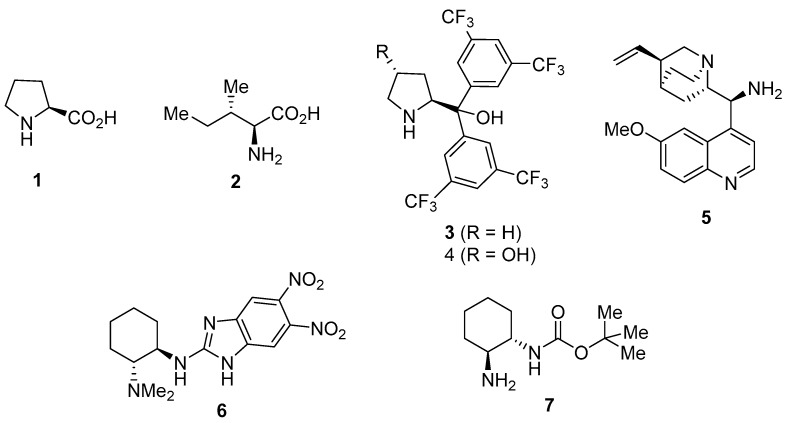
Chiral organocatalysts employed in enantioselective reactions performed in deep eutectic solvents (DESs).

Therefore, the combination of asymmetric organocatalysis and the use of DESs as solvents would show quite powerful and promising in order to achieve the enantioselective preparation of compounds of interest in an environmentally friendly manner. However, the application of DESs in asymmetric organocatalysis is still in its infancy, very few examples being reported (Figure 1). Thus, enantioselective aldol reactions have been performed in DESs using the amino acids L-proline (**1**) [15,16] and L-isoleucine (**2**) [17] as organocatalysts, as well as a combination of diaryl prolinols **3** [18] or **4** [19] and an enzyme. In addition, some enantioselective conjugate additions have been performed in DESs, such as the reaction of isobutyraldehyde and β-nitrostyrene and other conjugate addition reactions using 9-amino-9-deoxy-*epi*-quinine (**5**) as catalyst [20], the reaction of 1,3-dicarbonyl compounds with β-nitrostyrenes organocatalyzed by 2-amino benzimidazole **6** [21], and the reaction of aldehydes and maleimides organocatalyzed by primary-amine monocarbamate **7** [22]. Moreover, benzimidazole *ent*-**6** has also been used as catalyst for the α-amination of 1,3-dicarbonyl compounds in DESs [23].

**Figure 2:**
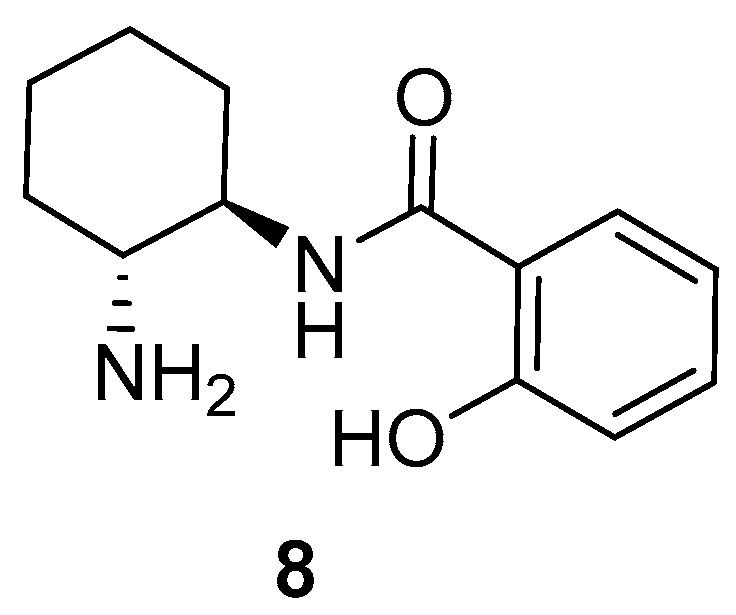
Organocatalyst employed in this study.

Recently, our group has employed a simple chiral primary amine-monosalicylamide from *trans*-cyclohexane-1,2-diamine **8** (Figure 2) and *ent*-**8** as organocatalysts in the enantioselective Michael addition of the “difficult” α,α-disubstituted aldehydes to maleimides [24] and β-nitroalkenes [25], obtaining the corresponding succinimides and γ-nitroaldehydes, respectively, in excellent chemical yields and enantioselections, but working in conventional VOCs as reaction media. The enantioselective preparation of succinimides and γ-nitroaldehydes shows interest, as the succinimide moiety is present in natural products and drug candidates [26,27,28], and also can be transformed into interesting compounds such as γ-lactams [29], which are important in the treatment of HIV [30] and neurological disorders [31,32]. In addition, γ-nitroaldehydes are precursors of γ-aminobutyric acid analogues (GABAs), which exhibit many pharmacological activities including antidepressant, anticonvulsant, anxiolytic and others [33,34], as well as can be potent drugs in the treatment of neurodegenerative disorders [35].

Therefore, the asymmetric preparation of these compounds using **8** as catalyst would gain considerably from an environmentally point if the mentioned preparations could be performed in DESs. Thus, we present now the enantioselective addition of aldehydes to maleimides and nitroalkenes organocatalyzed by **8** using DESs as reaction media.

## 2. Results and Discussion

The primary amine-salicylamide **8** was prepared as described by monoamidation of (1*R*,2*R*)-cyclohexane-1,2-diamine with phenyl salicylate in refluxing propan-2-ol [25]. Initially, we explored the reaction of aldehydes with maleimides in DESs, using the model conjugate addition of isobutyraldehyde (**9a**) to *N*-phenylmaleimide (**10a**) (Table 1).

Thus, the reaction organocatalyzed by **8** (10 mol%) carried out in several choline chloride (ChCl)-containing DESs (1:2 molar ratio) at room temperature afforded the corresponding substituted succinimide (*S*)-**11aa** after 2 d reaction time (Table 1, entries 1–4). The (*S*) absolute configuration of the final adduct was determined by comparison of the elution order of the corresponding enantiomers in chiral HPLC with those in the literature [36]. The DES resulting in a higher enantioselection was the formed by the mixture 1ChCl/2EG (EG = ethylene glycol), which gave **11aa** in 80% *ee* (Table 1, entry 3). The use of a mixture of 1Ph_3_MePBr/2Gly as DES gave a similar enantioselectivity, but slightly lower conversion (Table 1, entry 5). In addition, we also assayed the influence of the addition of some acid or basic additives. Thus, acid additives improved the enantioselectivity of the reaction (Table 1, entries 6–9), the best *ee* for **11aa** (88%) being obtained when 4-nitrobenzoic acid was used (Table 1, entry 8). The addition of a basic additive such as imidazole gave very similar results, but the addition of 4-(dimethylamino)pyridine (DMAP) gave rise to decomposition products. We also increased and lowered the loading of organocatalyst and the best additive but without achieving better results (Table 1, entries 12–14).

Next, we extended this enantioselective reaction to other maleimides 10 under the best conditions [**8** (10 mol%), 4-O_2_NC_6_H_4_CO_2_H (10 mol%), 1ChCl/2EG, rt], the results being summarized in Table 2. The absolute configuration of the known succinimides 11 was assigned in accordance with the elution order of the enantiomers in chiral HPLC (see the Experimental Section). Thus, when **9a** reacted with *N*-aryl maleimides **10b** and **10c** bearing electron-donating groups on the phenyl ring, such as 4-methyl and 4-methoxy, adducts **11ab** and **11ac** were obtained in 66 and 86% *ee*, respectively (Table 2, entries 2 and 3). When *N*-aryl maleimides **10d** and **10e**, bearing chloro and bromo groups at the *para*-position, were used, succinimides **11ad** and **11ae** were isolated in similar 81 and 78% *ee*, respectively (Table 2, entries 4 and 5). Interestingly, the reaction of isobutyraldehyde with maleimides non-*N*-arylated such as *N*-methylmaleimide (**10f**) or even the simple maleimide (**10g**), gave rise to the corresponding succinimides **11af** and **11ag** in almost quantitative yield and with enantioselectivities of 78 and 73%, respectively (Table 2, entries 6 and 7). We also employed cyclohexanecarbaldehyde (**9b**) as reacting aldehyde with *N*-phenylmaleimide (**10a**), although isolated yield and enantioselectivity for the corresponding adduct **11ba** diminished (Table 2, entry 8). Moreover, we also explored the performance of organocatalyst **7** (Figure 1), instead of **8**, in the case of entry 1 (Table 2), but we obtained adduct 11aa in only 73% *ee*. 

Next, we explored the suitability of DESs as reaction media in the reaction of isobutyraldehyde (**9a**) with β-nitrostyrene (**12a**) organocatalyzed by **8** (10 mol%) to give γ-nitroaldehyde **13aa** (Table 3). Thus, when ChCl-including mixtures were employed as DESs, adduct **13aa** was obtained (Table 3, entries 1–4), the (*R*) absolute configuration of the final adduct being determined by comparison of the elution order of the corresponding enantiomers in chiral HPLC with those in the literature [37]. However, the results were rather disappointing, the conversions being low-to-very low. The conversion rose up dramatically when employing as DES the mixture 1Ph_3_MePBr/2Gly, but the enantioselection was very poor (Table 3, entry 5). The addition of different acid or basic additives when the DESs 1ChCl/2Urea, 1ChCl/2Gly or 1ChCl/2EG were used did not give higher conversions, whereas the addition of additives when the DES 1Ph_3_MePBr/2Gly was employed did not afford a higher enantioselectivity. However, in the case of using 1ChCl/2H_2_O, we observed that the presence of basic additives certainly had an influence on the conversion and the enantioselectivity to **13aa** (Table 3, entries 6–8). Thus, the use of 1,4-diazabicyclo[2.2.2]octane (DABCO) (10 mol%) as basic additive gave rise to quantitative conversion to **13aa** with 55% *ee* (Table 3, entry 7), whereas the use of DMAP (10 mol%) afforded also quantitative conversion and 75% *ee* (Table 3, entry 8). On the contrary, the addition of acid additives such as benzoic acid or hexanedioic acid (HDA) lowered down the conversion dramatically (Table 3, entries 9 and 10). Finally, lowering or increasing the amount of organocatalyst **8** and DMAP gave a lower conversion or enantioselection (Table 3, entries 11 and 12).

Using the above obtained more efficient reaction conditions [8 (10 mol%), DMAP (10 mol%), 1ChCl/2H_2_O, rt], we explored the use of other β-nitroolefins in the enantioselective conjugate addition reaction with isobutyraldehyde (Table 4). The absolute configuration of the known γ-nitroaldehydes 13a was assigned in accordance with the elution order of the enantiomers in chiral HPLC when compared to the literature (see the Experimental Section).

Thus, the reaction of **9a** with nitroalkenes **12b** and **12c**, bearing electron-releasing groups such as 4-methyl or 4-methoxy in the aromatic ring, were used, the corresponding adducts **13ab** and **13ac** were isolated with similar enantioselectivities (Table 4, entries 2 and 3). The presence of a dioxolane system on the aromatic ring in the nitroolefin **12d** raised the enantioselection up to 75% (Table 4, entry 4), whereas this was lowered down to 68% when **12e** containing three methoxy groups was used as electrophile (Table 2, entry 5). When a 4-fluoro group was present (**12f**) the corresponding **13af** was isolated in 72% *ee*, whereas a chloro group at 2-position (**12g**) afforded adduct **13ag** in 80% *ee* (Table 4, entries 6 and 7). The presence of a 4-chloro (**12h**) gave **13ah** in only a 53% *ee* (Table 4, entry 8), whereas the presence of bromo groups in 2- and 4- position gave the corresponding products with enantioselectivities of 80 and 70%, respectively (Table 4, entries 9 and 10). In addition, an electron-withdrawing group such as the 4-trifluoromethyl (**12k**) gave a 70% enantioselectivity for **13ak** (Table 4, entry 11). Moreover, when the nitroalkene **12l**, bearing a 2-naphthyl group, was employed as Michael acceptor, the corresponding adduct **13al** was obtained in 75% *ee* (Table 4, entry 12), whereas the use of a 2-furanyl-containing nitroalkene **12m** gave rise to adduct **13am** in 76% *ee* (Table 4, entry 13). Furthermore, we also explored the behavior of organocatalyst **7** (Figure 1) when used instead of **8** in the reaction described in entry 1 (Table 4) but adduct **13aa** was obtained in a lower 69% *ee*.

The possibility of reusing the DES is very important in a synthetic methodology performed using these neoteric solvents. Therefore, we explored the reusability of the DES, and the catalytic system, by carrying out different reaction cycles of the model conjugate addition reactions performed under the best reaction conditions depicted in entry 1 of Table 2 and Table 4.

Thus, we explored the reusability of the system in the model reaction of isobutyraldehyde (**9a**) and *N*-phenylmaleimide (**10a**) (Table 2, entry 1). Once the reaction was finished, 2-methyltetrahydrofuran (2-MeTHF) was added and the resulting mixture was stirred vigorously. After the two layers settled down, the upper layer, containing the final adduct **11aa**, was separated. ^1^H NMR analysis of this crude revealed that the 4-nitrobenzoic acid used as additive, as well as a small amount of ethylene glycol, were also extracted from the DES, although no traces of the organocatalyst were observed. Refreshing the catalytic system by the addition of new additive (but no new chiral organocatalyst **8**) to the recovered DES allowed to obtain **11aa** in a second reaction cycle with similar conversion and identical enantioselectivity than when used for the first time. Following this recovery procedure, the DES containing the organocatalyst **8** was used in the third cycle, but the conversion diminished, although the enantioinduction remained similar (Table 5).

In addition, we also explored the reusability of the system DES/organocatalyst in the model reaction of isobutyraldehyde (**9a**) and β-nitrostyrene (**12a**) in 1ChCl/2H_2_O (Table 4, entry 1). Thus, performing a similar extraction than in the previous case using 2-MeTHF, we could also observe (^1^H NMR) the necessity of adding new additive (DMAP) after the first reaction cycle, but no leaching of organocatalyst **8** was detected. Again, refreshing the catalytic system by the addition of new DMAP as additive (but no new **8**) to the recovered DES, allowed to obtain **13aa** in a second reaction cycle with identical enantioselectivity than in the first one (Table 6). The DES containing the organocatalyst **8** was reused in an additional cycle with a decrease in the conversion but essentially without diminishing the achieved enantioinduction (Table 6). The reason for the observed decrease in this case (and in the former) is not totally clear. Probably, the structure of the DES results somehow degraded in the extraction/recovery process.

## 3. Experimental Section

### 3.1. General Information

All the reagents and solvents employed were of the best grade available and were used without further purification. Isobutyraldehyde was distilled. Organocatalyst **8** was obtained as reported [25]. Nitroolefins **12** were purchased or prepared according to a described procedure [38]. The ^1^H and ^13^C spectra were recorded at room temperature on a Bruker Oxford (Bruker, Billerica, MA, USA) AV300 at 300 MHz and on a Bruker Oxford AV400 at 101 MHz, respectively, using TMS as internal standard. Absolute configurations for adducts **11** and **13a** were determined according to the order of elution of their enantiomers in chiral HPLC. Reference racemic samples of adducts **11** and **13a** were obtained by performing the conjugate addition reactions using 4-methoxybenzylamine (20 mol%) as organocatalyst in toluene as solvent at room temperature.

### 3.2. General Procedure for the Preparation of DESs

A mixture of the two components, with the specified molar ratio, was added to a round bottom flask and the mixture was stirred for 60 min in a temperature range between 65 and 80 °C, obtaining the corresponding DES [39].

### 3.3. General Procedure for the Enantioselective Conjugate Addition of Aldehydes to Maleimides

To a mixture of catalyst **8** (4.7 mg, 0.02 mmol), 4-nitrobenzoic acid (3.3 mg, 0.02 mmol) and maleimide **10** (0.2 mmol) in ChCl/EG (1/2 molar ratio, 0.5 mL) was added the aldehyde **9** (0.4 mmol), and the reaction was vigorously stirred at rt until completion (TLC) (Table 2). After this period, HCl 2N (10 mL) was added and the reaction product was extracted with AcOEt (3 × 10 mL). The combined organic phases were washed with saturated NaHCO_3_ (10 mL) and brine (10 mL), dried over MgSO_4_ and, after filtration, the solvent was evaporated under reduced pressure (15 torr) to get the crude product, which was purified by flash column chromatography on silica gel (*n*-hexane/AcOEt gradients). The adducts 11 were identified by comparison of their NMR data with those of the literature (Supplementary Materials, NMR spectra). Their enantiomeric excesses were determined by chiral HPLC on the reaction crude, using the conditions described in each case (Supplementary Materials, HPLC chromatograms).

*2-(2,5-Dioxo-1-phenylpyrrolidin-3-yl)-2-methylpropanal* (**11aa**) [36]. White solid (48 mg, 98%); ^1^H NMR (CDCl_3_): δ_H_ = 9.52 (s, 1H), 7.51−7.43 (m, 2H), 7.42–7.36 (m, 1H), 7.31−7.26 (m, 2H), 3.15 (dd, *J* = 9.5, 5.5 Hz, 1H), 2.98 (dd, *J* = 18.3, 9.5 Hz, 1H), 2.62 (dd, *J* = 18.3, 5.5 Hz, 1H), 1.33 (s, 3H), 1.29 (s, 3H) ppm; ^13^C NMR (CDCl_3_): δ_C_ = 202.9, 177.0, 174.9, 131.9, 129.3, 128.9, 126.7, 48.7, 45.1, 32.0, 20.5, 19.8 ppm; HPLC: Chiralcel OD-H, λ = 240 nm, *n*-hexane/2-propanol, 80:20, 1.0 mL/min, t_r_ (*major*) = 25.0 min, t_r_ (*minor*) = 30.6 min.

*2-(2,5-Dioxo-1-(p-tolyl)pyrrolidin-3-yl)-2-methylpropanal* (**11ab**) [36]. White solid (46 mg, 88%); ^1^H NMR (CDCl_3_): δ_H_ = 9.49 (s, 1H), 7.31−7.22 (m, 2H), 7.17−7.08 (m, 2H), 3.12 (dd, *J* = 9.5, 5.5 Hz, 1H), 2.93 (dd, *J* = 18.3, 9.5 Hz, 1H), 2.57 (dd, *J* = 18.3, 5.5 Hz, 1H), 2.37 (s, 3H), 1.28 (s, 3H), 1.24 (s, 3H) ppm; ^13^C NMR (CDCl_3_): δ_C_ = 202.9, 177.1, 175.0, 138.8, 129.9, 129.2, 126.4, 48.5, 45.0, 31.8, 21.2, 20.3, 19.4 ppm; HPLC: Chiralcel OD-H, λ = 230 nm, *n*-hexane/2-propanol, 80:20, 1.0 mL/min, t_r_ (*major*) = 19.4 min, t_r_ (*minor*) = 22.6 min.

*2-(1-(4-Methoxyphenyl)-2,5-dioxopyrrolidin-3-yl)-2-methylpropanal* (**11ac**) [40]. White solid (54 mg, 98%); ^1^H NMR (CDCl_3_): δ_H_ = 9.52 (s, 1H), 7.23−7.14 (m, 2H), 7.02−6.93 (m, 2H), 3.82 (s, 3H), 3.14 (dd, *J* = 9.5, 5.4 Hz, 1H), 2.97 (dd, *J* = 18.2, 9.5 Hz, 1H), 2.60 (dd, *J* = 18.2, 5.4 Hz, 1H), 1.32 (s, 3H), 1.28 (s, 3H) ppm; ^13^C NMR (CDCl_3_): δ_C_ = 202.9, 177.3, 175.2, 159.7, 127.9, 124.5, 114.6, 55.6, 48.6, 45.1, 31.9, 20.4, 19.7 ppm; HPLC: Chiralpak AS-H, λ = 240 nm, *n*-hexane/2-propanol, 80:20, 1.0 mL/min, t_r_ (*major*) = 31.0 min, t_r_ (*minor*) = 34.8 min.

*2-(1-(4-Chlorophenyl)-2,5-dioxopyrrolidin-3-yl)-2-methylpropanal* (**11ad**) [36]. White solid (33 mg, 59%); ^1^H NMR (CDCl_3_): δ_H_ = 9.49 (s, 1H), 7.51−7.38 (m, 2H), 7.31−7.20 (m, 2H), 3.11 (dd, *J* = 9.5, 5.4 Hz, 1H), 2.97 (dd, *J* = 18.1, 9.5 Hz, 1H), 2.61 (dd, *J* = 18.1, 5.4 Hz, 1H), 1.36 (s, 3H), 1.29 (s, 3H) ppm; ^13^C NMR (CDCl_3_): δ_C_ = 202.8, 176.8, 174.6, 134.6, 130.4, 129.5, 127.9, 48.8, 45.1, 32.1, 20.6, 20.0 ppm; HPLC: Chiralcel OD-H, λ = 230 nm, *n*-hexane/2-propanol, 80:20, 1.0 mL/min, t_r_ (*major*) = 21.2 min, t_r_ (*minor*) = 35.6 min.

*2-(1-(4-Bromophenyl)-2,5-dioxopyrrolidin-3-yl)-2-methylpropanal* (**11ae**) [36]. White solid (55 mg, 85%); ^1^H NMR (CDCl_3_): δ_H_ = 9.48 (s, 1H), 7.64−7.55 (m, 2H), 7.23−7.14 (m, 2H), 3.11 (dd, *J* = 9.5, 5.5 Hz, 1H), 2.97 (dd, *J* = 18.2, 9.5 Hz, 1H), 2.61 (dd, *J* = 18.2, 5.5 Hz, 1H), 1.35 (s, 3H), 1.28 (s, 3H) ppm; ^13^C NMR (CDCl_3_): δ_C_ = 202.8, 176.7, 174.5, 132.5, 130.9, 128.2, 122.7, 48.8, 45.1, 32.1, 20.6, 20.0 ppm; HPLC: Chiralcel OD-H, λ = 240 nm, *n*-hexane/2-propanol, 80:20, 1.0 mL/min, t_r_ (*major*) = 22.2 min, t_r_ (*minor*) = 34.9 min.

*2-Methyl-2-(1-methyl-2,5-dioxopyrrolidin-3-yl)propanal* (**11af**) [22]. White solid (36 mg, 98%); ^1^H NMR (CDCl_3_): δ_H_ = 9.51 (s, 1H), 3.05 (dd, *J* = 9.2, 5.2 Hz, 1H), 2.99 (s, 3H), 2.83 (dd, *J* = 18.2, 9.2 Hz, 1H), 2.45 (dd, *J* = 18.2, 5.2 Hz, 1H), 1.22 (s, 3H), 1.21 (s, 3H) ppm; ^13^C NMR (CDCl_3_): δ_C_ = 202.9, 177.9, 175.9, 48.0, 45.1, 31.5, 24.9, 20.1, 19.2 ppm; HPLC: Chiralpak AS-H, λ = 210 nm, *n*-hexane/2-propanol, 80:20, 1.0 mL/min, t_r_ (*minor*) = 11.5 min, t_r_ (*major*) = 12.5 min.

*2-(2,5-Dioxopyrrolidin-3-yl)-2-methylpropanal* (**11ag**) [22]. White solid (33 mg, 98%); ^1^H NMR (CDCl_3_): δ_H_ = 9.49 (s, 1H), 9.03 (br. s, 1H), 3.10 (dd, *J* = 7.7, 5.5 Hz, 1H), 2.85 (dd, *J* = 18.3, 7.7 Hz, 1H), 2.50 (dd, *J* = 18.3, 5.5 Hz, 1H), 1.24 (s, 3H), 1.23 (s, 3H) ppm; ^13^C NMR (CDCl_3_): δ_C_ = 203.0, 178.5, 176.4, 48.1, 46.4, 32.9, 20.2, 19.4 ppm; HPLC: Chiralpak AD-H, λ = 210 nm, *n*-hexane/2-propanol, 80:20, 1.0 mL/min, t_r_ (*minor*) = 16.8 min, t_r_ (*major*) = 21.2 min.

*1-(2,5-Dioxo-1-phenylpyrrolidin-3-yl)cyclohexane-1-carbaldehyde* (**11ba**) [36]. White solid (36 mg, 63%); ^1^H NMR (CDCl_3_): δ_H_ = 9.54 (s, 1H), 7.56−7.33 (m, 3H), 7.33−7.26 (m, 2H), 3.22 (dd, *J* = 9.4, 6.0 Hz, 1H), 2.88 (dd, *J* = 18.1, 9.4 Hz, 1H), 2.68 (dd, *J* = 18.1, 6.0 Hz, 1H), 1.96 (m, 2H), 1.60 (m, 8H) ppm; ^13^C NMR (CDCl_3_): δ_C_ = 204.7, 177.2, 174.9, 132.1, 129.3, 128.8, 126.8, 52.3, 42.9, 31.7, 28.8, 28.3, 25.3, 21.6, 21.4 ppm; HPLC: Chiralcel OD-H, λ = 240 nm, *n*-hexane/2-propanol, 80:20, 1.0 mL/min, t_r_ (*major*) = 23.9 min, t_r_ (*minor*) = 30.9 min.

### 3.4. General Procedure for the Enantioselective Conjugate Addition of Isobutyraldehyde to Nitroalkenes

To a mixture of catalyst **8** (4.7 mg, 0.02 mmol), DMAP (2.4 mg, 0.02 mmol) and nitroalkene **12** (0.2 mmol) in ChCl/H_2_O (1/2 molar ratio, 0.5 mL) was added isobutyraldehyde **9a** (37 µL, 28.8 mg, 0.4 mmol), and the reaction was vigorously stirred at rt until completion (TLC) (Table 4). After this period, HCl 2N (10 mL) was added and the reaction product was extracted with AcOEt (3 × 10 mL). The combined organic phases were washed with saturated NaHCO_3_ (10 mL) and brine (10 mL), dried over MgSO_4_ and, after filtration, the solvent was evaporated under reduced pressure (15 torr) to get the crude product, which was purified by flash column chromatography on silica gel (*n*-hexane/AcOEt gradients). The adducts 13a were identified by comparison of their NMR data with those of the literature (Supplementary Materials, NMR spectra). Their enantiomeric excesses were determined by chiral HPLC on the reaction crude, using the conditions described in each case (Supplementary Materials, HPLC chromatograms).

*2,2-Dimethyl-4-nitro-3-phenylbutanal* (**13aa**) [37]. Yellow oil (41 mg, 92%); ^1^H NMR (CDCl_3_): δ_H_ = 9.53 (s, 1H), 7.39−7.28 (m, 3H), 7.24−7.15 (m, 2H), 4.86 (dd, *J* = 13.0, 11.2 Hz, 1H), 4.69 (dd, *J* = 13.0, 4.3 Hz, 1H), 3.78 (dd, *J* = 11.2, 4.3 Hz, 1H), 1.14 (s, 3H), 1.01 (s, 3H) ppm; ^13^C NMR (CDCl_3_): δ_C_ = 204.4, 135.5, 129.2, 128.9, 128.3, 76.5, 48.7, 48.4, 21.8, 19.1 ppm; HPLC: Chiralcel OD-H, λ = 210 nm, *n*-hexane/2-propanol, 80:20, 1.0 mL/min, t_r_ (*major*) = 12.2 min, t_r_ (*minor*) = 17.4 min.

*2,2-Dimethyl-4-nitro-3-(p-tolyl)butanal* (**13ab**) [41]. Yellow oil (28 mg, 60%); ^1^H NMR (CDCl_3_): δ_H_ = 9.53 (s, 1H), 7.18–7.03 (m, 4H), 4.83 (dd, *J* = 12.9, 11.3 Hz, 1H), 4.67 (dd, *J* = 12.9, 4.3 Hz, 1H), 3.74 (dd, *J* = 11.3, 4.3 Hz, 1H), 2.32 (s, 3H), 1.13 (s, 3H), 1.00 (s, 3H) ppm; ^13^C NMR (CDCl_3_): δ_C_ = 204.5, 138.1, 132.4, 129.6, 129.1, 76.6, 48.5, 48.4, 21.8, 21.2, 19.1 ppm; HPLC: Chiralcel OD-H, λ = 240 nm, *n*-hexane/2-propanol, 80:20, 1.0 mL/min, t_r_ (*major*) = 9.2 min, t_r_ (*minor*) = 12.4 min.

*3-(4-Methoxyphenyl)-2,2-dimethyl-4-nitrobutanal* (**13ac**) [37]. Yellow oil (37 mg, 74%); ^1^H NMR (CDCl_3_): δ_H_ = 9.52 (s, 1H), 7.17−7.05 (m, 2H), 6.91−6.79 (m, 2H), 4.81 (dd, *J* = 12.8, 11.3 Hz, 1H), 4.66 (dd, *J* = 12.8, 4.3 Hz, 1H), 3.79 (s, 3H), 3.73 (dd, *J* = 11.3, 4.3 Hz, 1H), 1.12 (s, 3H), 1.00 (s, 3H) ppm; ^13^C NMR (CDCl_3_): δ_C_ = 204.5, 159.5, 130.3, 127.3, 114.3, 76.7, 55.4, 48.5, 48.1, 21.7, 19.1 ppm; HPLC: Chiralcel OD-H, λ = 240 nm, *n*-hexane/2-propanol, 80:20, 1.0 mL/min, t_r_ (*major*) = 12.1 min, t_r_ (*minor*) = 16.4 min.

*3-(Benzo[d]* [1,3] *dioxol-5-yl)-2,2-dimethyl-4-nitrobutanal* (**13ad**) [25]. Yellow oil (36 mg, 67%); ^1^H NMR (CDCl_3_): δ_H_ = 9.51 (s, 1H), 6.75 (d, *J* = 7.9 Hz, 1H), 6.72−6.61 (m, 2H), 5.96 (s, 2H), 4.78 (dd, *J* = 12.9, 11.3 Hz, 1H), 4.65 (dd, *J* = 12.9, 4.3 Hz, 1H), 3.69 (dd, *J* = 11.3, 4.3 Hz, 1H), 1.13 (s, 3H), 1.02 (s, 3H) ppm; ^13^C NMR (CDCl_3_): δ_C_ = 204.4, 148.1, 147.6, 129.1, 122.8, 109.3, 108.5, 101.4, 76.7, 48.5 (2xC), 21.8, 19.2 ppm; HPLC: Chiralcel OD-H, λ = 230 nm, *n*-hexane/2-propanol, 80:20, 1.0 mL/min, t_r_ (*major*) = 15.8 min, t_r_ (*minor*) = 20.5 min.

*2,2-Dimethyl-4-nitro-3-(3,4,5-trimethoxyphenyl)butanal* (**13ae**) [25]. Yellow oil (45 mg, 73%); ^1^H NMR (CDCl_3_): δ_H_ = 9.52 (s, 1H), 6.38 (s, 2H), 4.85 (dd, *J* = 13.0, 11.2 Hz, 1H), 4.69 (dd, *J* = 13.0, 4.3 Hz, 1H), 3.84 (s, 6H), 3.83 (s, 3H), 3.69 (dd, *J* = 11.2, 4.3 Hz, 1H), 1.16 (s, 3H), 1.06 (s, 3H) ppm; ^13^C NMR (CDCl_3_): δ_C_ = 204.5, 153.6, 138.1, 131.2, 106.6, 76.6, 61.0, 56.4, 49.2, 48.4, 22.0, 19.6 ppm; HPLC: Chiralcel OD-H, λ = 210 nm, *n*-hexane/2-propanol, 80:20, 1.0 mL/min, t_r_ (*major*) = 15.5 min, t_r_ (*minor*) = 17.8 min.

*3-(4-Fluorophenyl)-2,2-dimethyl-4-nitrobutanal* (**13af**) [37]. Yellow oil (76 mg, 78%); ^1^H NMR (CDCl_3_): δ_H_ = 9.51 (s, 1H), 7.24−7.13 (m, 2H), 7.09−6.98 (m, 2H), 4.82 (dd, *J* = 13.0, 11.3 Hz, 1H), 4.69 (dd, *J* = 13.0, 4.3 Hz, 1H), 3.78 (dd, *J* = 11.3, 4.3 Hz, 1H), 1.13 (s, 3H), 1.01 (s, 3H) ppm; ^13^C NMR (CDCl_3_): δ_C_ = 204.1, 163.6, 161.6, 130.9, 130.8, 116.0, 115.8, 76.5, 48.4, 48.1, 21.8, 19.1 ppm; HPLC: Chiralcel OD-H, λ = 210 nm, *n*-hexane/2-propanol, 80:20, 1.0 mL/min, t_r_ (*major*) = 10.0 min, t_r_ (*minor*) = 15.6 min.

*3-(2-Chlorophenyl)-2,2-dimethyl-4-nitrobutanal* (**13ag**) [42]. Yellow oil (25 mg, 49%); ^1^H NMR (CDCl_3_): δ_H_ = 9.55 (s, 1H), 7.45–7.39 (m, 1H), 7.31–7.26 (m, 2H), 7.23 (m, 1H), 4.97–4.48 (m, 3H), 1.17 (s, 3H), 1.07 (s, 3H) ppm; ^13^C-NMR (CDCl_3_): δ_C_ = 203.9, 135.9, 133.9, 130.6, 129.3, 128.4, 127.3, 76.3, 49.2, 42.6, 21.0, 18.8 ppm; HPLC: Chiralcel OD-H, λ = 210 nm, *n*-hexane/2-propanol, 80:20, 1.0 mL/min, t_r_ (*major*) = 9.7 min, t_r_ (*minor*) = 23.2 min.

*3-(4-Chlorophenyl)-2,2-dimethyl-4-nitrobutanal* (**13ah**) [37]. Yellow oil (45 mg, 89%); ^1^H NMR (CDCl_3_): δ_H_ = 9.50 (s, 1H), 7.35−7.29 (m, 2H), 7.18−7.11 (m, 2H), 4.83 (dd, *J* = 13.1, 11.3 Hz, 1H), 4.69 (dd, *J* = 13.1, 4.2 Hz, 1H), 3.77 (dd, *J* = 11.3, 4.2 Hz, 1H), 1.13 (s, 3H), 1.01 (s, 3H) ppm; ^13^C-NMR (CDCl_3_): δ_C_ = 204.0, 134.3, 134.1, 130.5, 129.1, 76.3, 48.3, 48.1, 21.9, 19.1 ppm; HPLC: Chiralcel OD-H, λ = 210 nm, *n*-hexane/2-propanol, 80:20, 1.0 mL/min, t_r_ (*major*) = 11.2 min, t_r_ (*minor*) = 16.4 min.

*3-(2-Bromophenyl)-2,2-dimethyl-4-nitrobutanal* (**13ai**) [41]. Yellow oil (42 mg, 70%); ^1^H NMR (CDCl_3_): δ_H_ = 9.55 (s, 1H), 7.61 (dd, *J* = 8.0, 1.2 Hz, 1H), 7.36–7.25 (m, 2H), 7.15 (ddd, *J* = 8.0, 6.8, 2.2 Hz, 1H), 4.84 (dd, *J* = 13.2, 11.3 Hz, 1H), 4.72 (dd, *J* = 13.2, 4.1 Hz, 1H), 4.62 (dd, *J* = 11.3, 4.1 Hz, 1H), 1.17 (s, 3H), 1.09 (s, 3H) ppm; ^13^C-NMR (CDCl_3_): δ_C_ = 203.9, 135.7, 134.0, 129.6, 128.5, 128.0, 127.2, 76.6, 49.2, 45.4, 21.1, 18.9 ppm; HPLC: Chiralcel OD-H, λ = 230 nm, *n*-hexane/2-propanol, 80:20, 1.0 mL/min, t_r_ (*major*) = 10.5 min, t_r_ (*minor*) = 25.6 min.

*3-(4-Bromophenyl)-2,2-dimethyl-4-nitrobutanal* (**13aj**) [37]. Yellow oil (50 mg, 83%); ^1^H NMR (CDCl_3_): δ_H_ = 9.50 (s, 1H), 7.51−7.42 (m, 2H), 7.15−7.04 (m, 2H), 4.82 (dd, *J* = 13.1, 11.3 Hz, 1H), 4.69 (dd, *J* = 13.1, 4.2 Hz, 1H), 3.76 (dd, *J* = 11.3, 4.2 Hz, 1H), 1.12 (s, 3H), 1.01 (s, 3H) ppm; ^13^C NMR (CDCl_3_): δ_C_ = 203.9, 134.7, 132.0, 130.9, 122.4, 76.2, 48.2, 48.1, 21.9, 19.1 ppm; HPLC: Chiralcel OD-H, λ = 230 nm, *n*-hexane/2-propanol, 80:20, 1.0 mL/min, t_r_ (*major*) = 12.4 min, t_r_ (*minor*) = 17.4 min.

*2,2-Dimethyl-4-nitro-3-(4-(trifluoromethyl)phenyl)butanal* (**13ak**) [43]. Yellow oil (49 mg, 85%); ^1^H NMR (CDCl_3_): δ_H_ = 9.50 (s, 1H), 7.61 (d, *J* = 8.1 Hz, 2H), 7.36 (d, *J* = 8.1 Hz, 2H), 4.89 (dd, *J* = 13.3, 11.4 Hz, 1H), 4.74 (dd, *J* = 13.3, 4.1 Hz, 1H), 3.87 (dd, *J* = 11.4, 4.1 Hz, 1H), 1.15 (s, 3H), 1.03 (s, 3H) ppm; ^13^C NMR (CDCl_3_): δ_C_ = 203.7, 139.9, 129.7, 125.9 (2xC), 125.8 (2xC), 76.1, 48.4, 48.3, 22.0, 19.1 ppm; HPLC: Chiralcel OD-H, λ = 210 nm, *n*-hexane/2-propanol, 80:20, 1.0 mL/min, t_r_ (*major*) = 10.4 min, t_r_ (*minor*) = 16.2 min.

*2,2-Dimethyl-3-(naphthalen-2-yl)-4-nitrobutanal* (**13al**) [37]. Yellow oil (36 mg, 67%); ^1^H NMR (CDCl_3_): δ_H_ = 9.55 (s, 1H), 7.84−7.77 (m, 3H), 7.66 (d, *J* = 1.4 Hz, 1H), 7.52−7.45 (m, 2H), 7.31 (dd, *J* = 8.6, 1.9 Hz, 1H), 4.98 (dd, *J* = 13.1, 11.3 Hz, 1H), 4.76 (dd, *J* = 13.1, 4.2 Hz, 1H), 3.95 (dd, *J* = 11.3, 4.2 Hz, 1H), 1.17 (s, 3H), 1.04 (s, 3H) ppm; ^13^C NMR (CDCl_3_): δ_C_ = 204.4, 133.2, 133.1, 133.0, 128.6, 128.5, 128.0, 127.8, 126.7, 126.6, 126.5, 76.5, 48.8, 48.6, 22.0, 19.2 ppm; HPLC: Chiralcel OD-H, λ = 280 nm, *n*-hexane/2-propanol, 75:25, 1.0 mL/min, t_r_ (*major*) = 15.6 min, t_r_ (*minor*) = 18.1 min.

*3-(Furan-2-yl)-2,2-dimethyl-4-nitrobutanal* (**13am**) [37]. Yellow oil (32 mg, 75%); ^1^H NMR (CDCl_3_): δ_H_ = 9.52 (s, 1H), 7.37 (d, *J* = 1.9 Hz, 1H), 6.32 (dd, *J* = 3.2, 1.9 Hz, 1H), 6.22 (dd, *J* = 7.1, 3.2 Hz, 1H), 4.76 (dd, *J* = 12.9, 11.0 Hz, 1H), 4.59 (dd, *J* = 12.9, 3.9 Hz, 1H), 3.92 (dd, *J* = 11.0, 3.9 Hz, 1H), 1.18 (s, 3H), 1.05 (s, 3H) ppm; ^13^C NMR (CDCl_3_): δ_C_ = 203.6, 149.9, 142.9, 110.6, 109.8, 75.0, 48.3, 42.4, 21.3, 19.3 ppm; HPLC: Chiralcel OD-H, λ = 230 nm, *n*-hexane/2-propanol, 80:20, 1.0 mL/min, t_r_ (*major*) = 8.1 min, t_r_ (*minor*) = 11.8 min.

### 3.5. General Procedure for Recycling Experiments

To a mixture of catalyst **8** (4.7 mg, 0.02 mmol), additive (4-nitrobenzoic acid or DMAP, 0.02 mmol) and *N*-phenylmaleimide **10a** (34.6 mg, 0.2 mmol) or β-nitrostyrene **12a** (29.8 mg, 0.2 mmol) in the corresponding DES (0.5 mL) was added isobutyraldehyde **9a** (37 µL, 28.8 mg, 0.4 mmol), and the reaction was vigorously stirred for 2 days at rt. After this period, 2-MeTHF (3 mL) was added and the mixture was stirred for 10 min at rt. The stirring was stopped to allow phase separation and the upper organic layer was removed with a pipette. This extractive procedure was repeated two more times combining the organic extracts, which were washed with water (3 × 5 mL), dried over MgSO_4_, filtered and evaporated under reduced pressure (15 torr) to afford the reaction product. The residual volatile organic solvent present in the DES phase was removed under vacuum evaporation (15 torr) and the catalytic system was regenerated by addition of new additive (0.02 mmol) (in the case of the reaction of isobutyraldehyde and *N*-phenylmaleimide, 140 μL of additional ethylene glycol were added). The next reaction cycle was performed with the DES mixture adding isobutyraldehyde and *N*-phenylmaleimide or β-nitrostyrene. Once the reaction was finished, the reaction mixture was subjected again to the above-described procedure and further reaction cycles were repeated using the recycled DES phase containing **8**.

## 4. Conclusions

We concluded that a primary amine-salicylamide, prepared by a simple monoamidation of an enantiomerically pure *trans*-cyclohexane-1,2-diamine, could act as chiral organocatalyst suitable for the enantioselective conjugate addition of aldehydes to maleimides or nitroolefins carried out in deep eutectic mixtures as “green” solvents. Good yields and enantioselectivities could be achieved working in choline chloride/ethylene glycol (for maleimides) and choline chloride/water (for nitroolefins), the presence of acid and basic additives, respectively, being necessary. The eutectic solvent containing the organocatalyst could be recycled and reused affording similar enantioselectivities.

## Figures and Tables

**Table 1:**
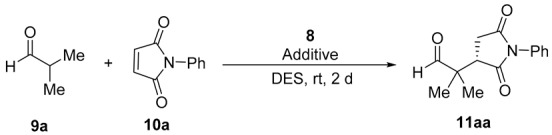
Screening and optimization of the reaction conditions for the model enantioselective conjugate addition of isobutyraldehyde to *N*-phenylmaleimide in DESs organocatalyzed by **8**.

Entry	8 mol%	Additive (mol%) ^a^	DES ^a,b^	Conv. (%) ^c^	*ee* (%) ^d^
1	10	-	ChCl/Urea	27	24
2	10	-	ChCl/Gly	40	63
3	10	-	ChCl/EG	99	80
4	10	-	ChCl/H_2_O	96	19
5	10	-	Ph_3_MePBr/Gly	96	79
6	10	PhCO_2_H (10)	ChCl/EG	99	82
7	10	4-MeOC_6_H_4_CO_2_H (10)	ChCl/EG	99	81
8	10	4-O_2_NC_6_H_4_CO_2_H (10)	ChCl/EG	99	88
9	10	HDA	ChCl/EG	99	86
10	10	Imidazole (10)	ChCl/EG	98	87
11	10	DMAP (10)	ChCl/EG	dec.	n.d.
12	10	4-O_2_NC_6_H_4_CO_2_H (20)	ChCl/EG	99	78
13	5	4-O_2_NC_6_H_4_CO_2_H (5)	ChCl/EG	63	81
14	20	4-O_2_NC_6_H_4_CO_2_H (20)	ChCl/EG	99	82

^a^ Abbreviations: HDA: hexanedioic acid; DMAP: 4-(dimethylamino)pyridine; ChCl: choline chloride; Gly: glycerol; EG: ethylene glycol. ^b^ 1:2 molar ratio. ^c^ Determined by ^1^H NMR (300 MHz). ^d^ Enantioselectivities and absolute stereochemistry determined by chiral HPLC.

**Table 2:**
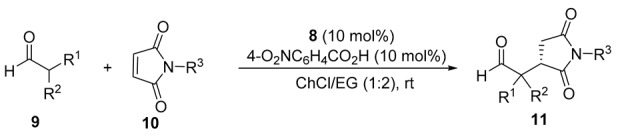
Enantioselective conjugate addition of aldehydes to maleimides organocatalyzed by **8** in a DES.

Entry	Aldehyde		Maleimide		Time (day)	Succinimide		
	R^1^,R^2^	No.	R^3^	No.		No.	Yield (%) ^a^	*ee*^b^ (%) ^b^
1	Me,Me	**9a**	Ph	**10a**	2	**11aa**	98	88
2	Me,Me	**9a**	4-MeC_6_H_4_	**10b**	2	**11ab**	88	66
3	Me,Me	**9a**	4-MeOC_6_H_4_	**10c**	2	**11ac**	98	86
4	Me,Me	**9a**	4-ClC_6_H_4_	**10d**	2	**11ad**	59	81
5	Me,Me	**9a**	4-BrC_6_H_4_	**10e**	2	**11ae**	85	78
6	Me,Me	**9a**	Me	**10f**	2	**11af**	98	78
7	Me,Me	**9a**	H	**10g**	2	**11ag**	98	73
8	-(CH_2_)_5_-	**9b**	Ph	**10a**	4	**11ba**	63	41

^a^ Isolated yield after flash chromatography. ^b^ Enantioselectivities determined by chiral HPLC. Absolute configuration assigned by the order of elution of the enantiomers in chiral HPLC (see the Experimental Section).

**Table 3:**
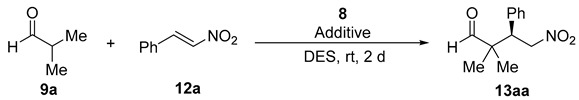
Screening and optimization of the reaction conditions for the model enantioselective conjugate addition of isobutyraldehyde to *trans*-β-nitrostyrene in DES organocatalyzed by **8**.

Entry	8 mol%	Additive (mol%) ^a^	DES ^a,b^	Conv. (%) ^c^	*ee* (%) ^d^
1	10	-	ChCl/Urea	35	57
2	10	-	ChCl/Gly	7	n.d.
3	10	-	ChCl/EG	16	74
4	10	-	ChCl/H_2_O	18	15
5	10	-	Ph_3_MePBr/Gly	99	11
6	10	Imidazole (10)	ChCl/H_2_O	30	62
7	10	DABCO (10)	ChCl/H_2_O	99	55
8	10	DMAP (10)	ChCl/H_2_O	99	75
9	10	PhCO_2_H (10)	ChCl/H_2_O	3	n.d.
10	10	HDA (10)	ChCl/H_2_O	12	n.d.
11	5	DMAP (5)	ChCl/H_2_O	81	73
12	20	DMAP (20)	ChCl/H_2_O	99	64

^a^ Abbreviations: DABCO: 1,4-diazabicyclo[2.2.2]octane; DMAP: 4-(dimethylamino)pyridine; HDA: hexanedioic acid; ChCl: choline chloride; Gly: glycerol; EG: ethylene glycol. ^b^ 1:2 molar ratio. ^c^ Determined by ^1^H NMR (300 MHz). ^d^ Enantioselectivities and absolute stereochemistry determined by chiral HPLC.

**Table 4:**
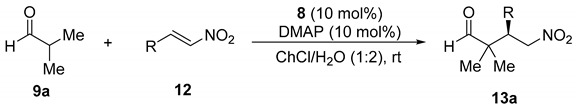
Enantioselective conjugate addition of isobutyraldehyde to nitroalkenes organocatalyzed by **8** in a DES.

Entry	Nitroalkene		Time (day)	γ-Nitroaldehyde		
	R	No.		No.	Yield (%) ^a^	*ee* (%)^b^
1	Ph	**12a**	2	**13aa**	92	75
2	4-MeC_6_H_4_	**12b**	1	**13ab**	60	61
3	4-MeOC_6_H_4_	**12c**	1	**13ac**	74	63
4	3,4-(OCH_2_O)C_6_H_3_	**12d**	2	**13ad**	67	75
5	3,4,5-(MeO)_3_C_6_H_2_	**12e**	1	**13ae**	73	68
6	4-FC_6_H_4_	**12f**	2	**13af**	78	72
7	2-ClC_6_H_4_	**12g**	1	**13ag**	49	80
8	4-ClC_6_H_4_	**12h**	2	**13ah**	89	53
9	2-BrC_6_H_4_	**12i**	2	**13ai**	70	80
10	4-BrC_6_H_4_	**12j**	1	**13aj**	83	70
11	4-F_3_CC_6_H_4_	**12k**	1	**13ak**	85	70
12	2-Naphthyl	**12l**	1	**13al**	67	75
13	2-Furanyl	**12m**	2	**13am**	75	76

^a^ Isolated yield after flash chromatography. ^b^ Enantioselectivities determined by chiral HPLC. Absolute configuration assigned by the order of elution of the enantiomers in chiral HPLC (see the Experimental Section).

**Table 5:** Recycle experiments in the reaction of **9a** and **10a**. Conversions and *ee*’s of 11aa after consecutive reaction cycles.

Reaction Cycle	Conv. (%) ^a^	*ee* (%) ^b^
1	99	88
2	97	88
3	53	85

^a^ Determined by ^1^H NMR (300 MHz). ^b^ Enantioselectivity determined by chiral HPLC.

**Table 6:** Recycle experiments in the reaction of **9a** and **12a**. Conversions and *ee*’s of **13aa** after consecutive reaction cycles.

Reaction Cycle	Conv. (%) ^a^	*ee* (%) ^b^
1	99	75
2	93	75
3	59	74

^a^ Determined by ^1^H NMR (300 MHz). ^b^ Enantioselectivity determined by chiral HPLC.

## Supplementary Materials

The following are available online, NMR spectra and HPLC chromatograms.
