# Supplementary material for: Organocatalytic Asymmetric Conjugate Addition of Aldehydes to Maleimides and Nitroalkenes in Deep Eutectic Solvents
Source: Molecules. 2019 Nov 9;24(22):4058. doi: 10.3390/molecules24224058 (PMC6891809; doi:10.3390/molecules24224058)
Supplement: Supplementary file 1 [file molecules-24-04058-s001.pdf]

Supplementary Materials

Organocatalytic asymmetric conjugate addition of aldehydes to maleimides and nitroalkenes in deep eutectic solvents

Alejandro Torregrosa-Chinillach, Alba Sánchez-Laó, Elisa Santagostino, Rafael Chinchilla*

NMR Spectra

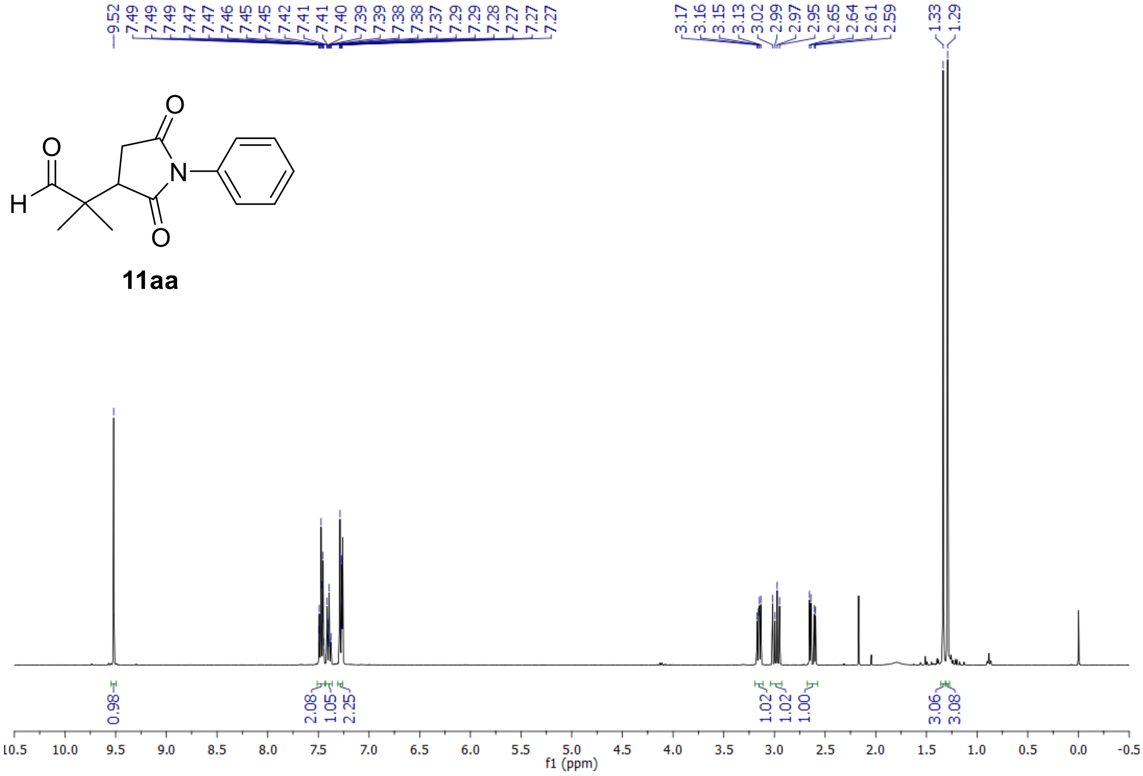

9.52
7.49
7.49
7.49
7.47
7.47
7.46
7.45
7.45
7.42
7.41
7.41
7.40
7.39
7.39
7.38
7.38
7.37
7.29
7.29
7.28
7.27
7.27
7.27
3.17
3.16
3.15
3.13
3.02
2.99
2.97
2.95
2.65
2.64
2.61
2.59
1.33
1.29
O
O
H
N
O
11aa
0.98
2.08
1.05
2.25
1.02
1.02
1.00
3.06
3.08
10.5
10.0
9.5
9.0
8.5
8.0
7.5
7.0
6.5
6.0
5.5
5.0
4.5
4.0
3.5
3.0
2.5
2.0
1.5
1.0
0.5
0.0
-0.5
f1 (ppm)

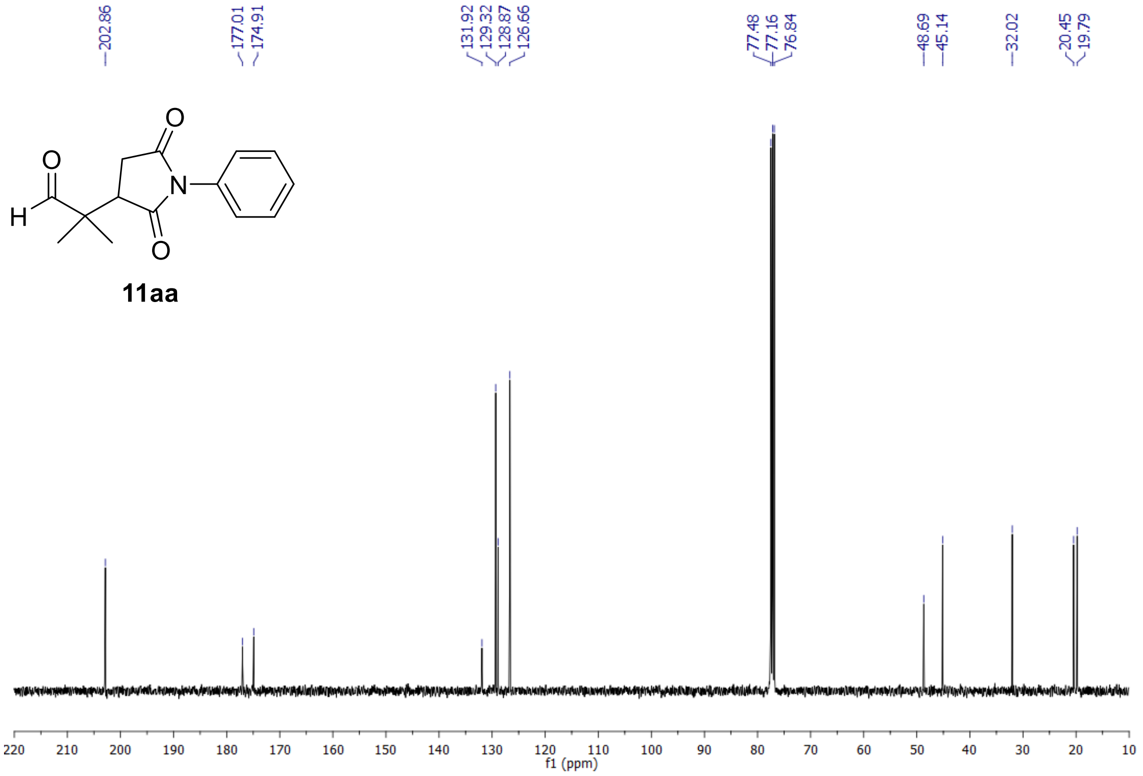

202.86
177.01
174.91
131.92
129.32
128.87
126.66
77.48
77.16
76.84
48.69
45.14
32.02
20.45
19.79
O
O
H
N
O
11aa
220
210
200
190
180
170
160
150
140
130
120
110
100
90
80
70
60
50
40
30
20
10
f1 (ppm)

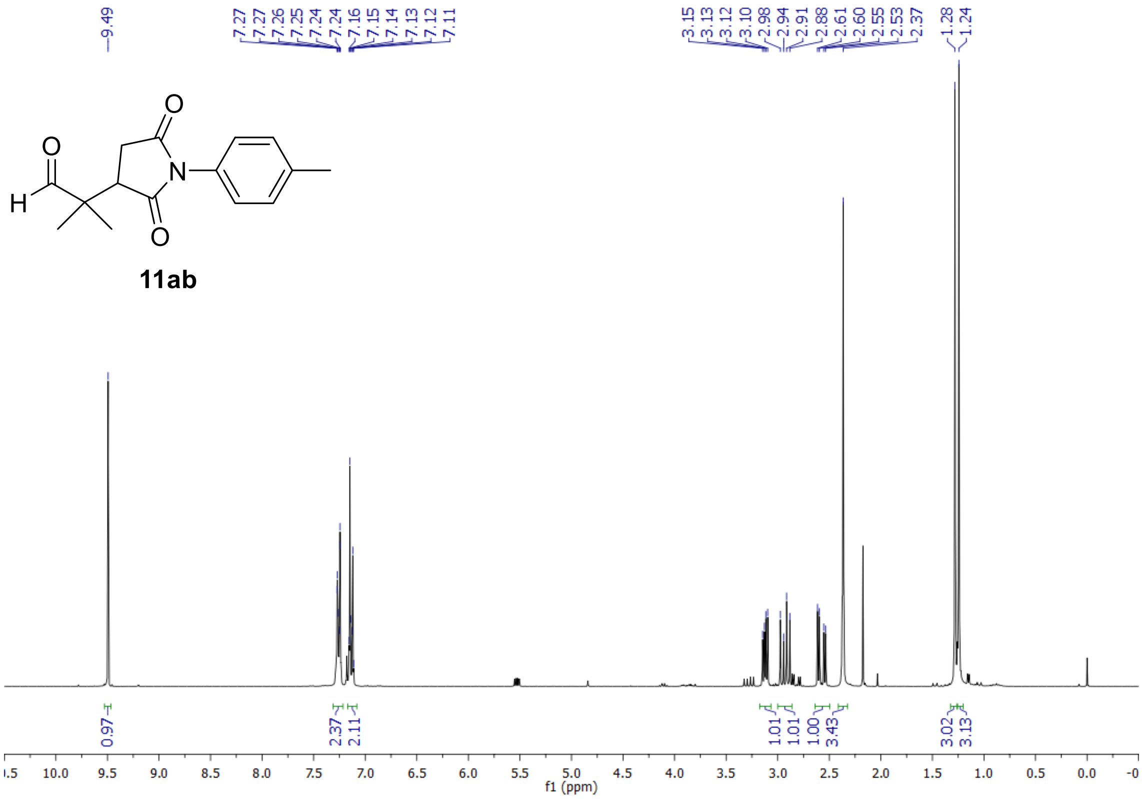
9.49
7.27 7.27 7.26 7.25 7.24 7.24 7.16 7.15 7.14 7.13 7.12 7.11
3.15 3.13 3.12 3.10 2.98 2.94 2.91 2.88 2.61 2.60 2.55 2.53 2.37
1.28 1.24
0.97 2.37 2.11 1.01 1.01 1.00 3.43 3.02 3.13
f1 (ppm)

11ab

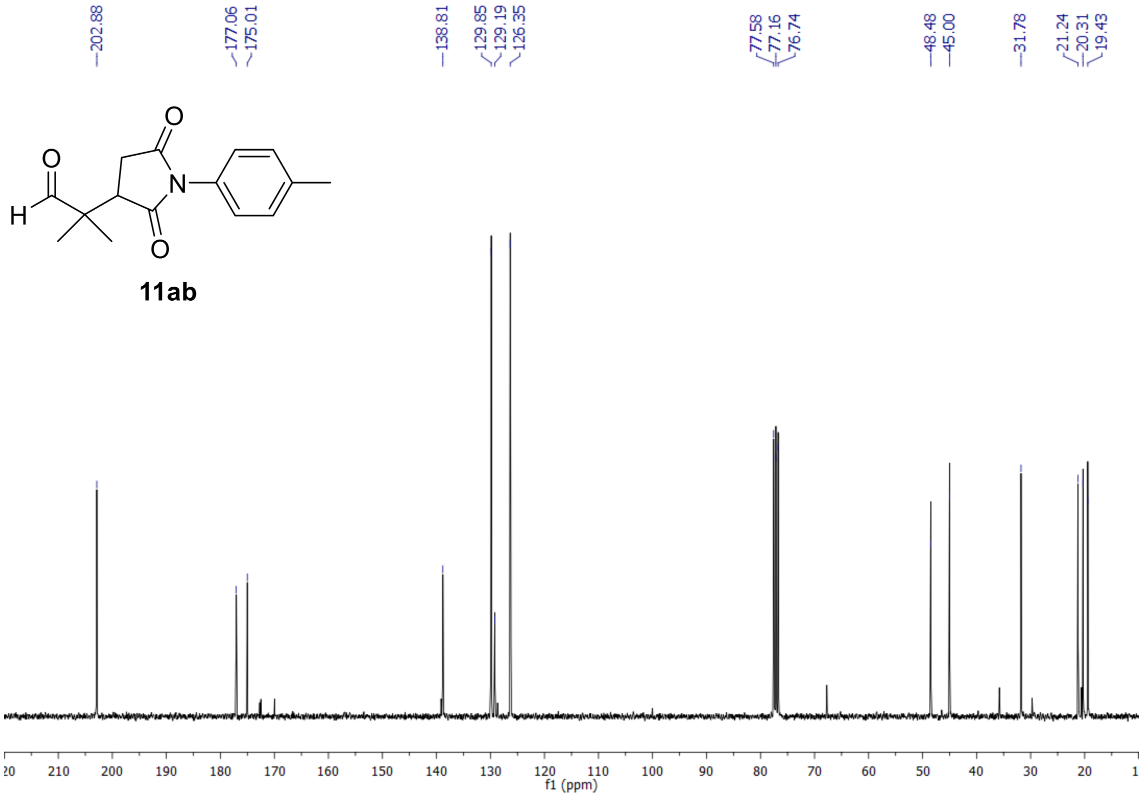
202.88
177.06 175.01
138.81 129.85 129.19 126.35
77.58 77.16 76.74
48.48 45.00
31.78
21.24 20.31 19.43
f1 (ppm)

11ab

11ac

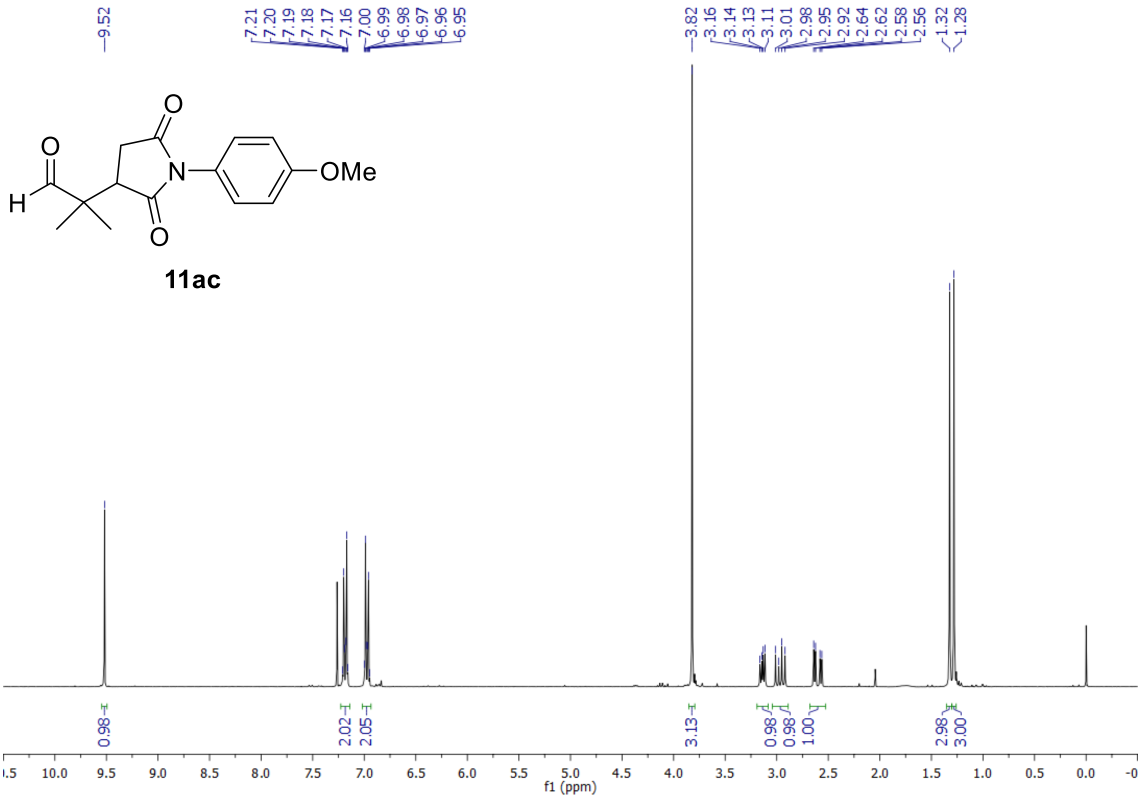

9.52
7.21
7.20
7.19
7.18
7.17
7.16
7.00
6.99
6.98
6.97
6.96
6.95
3.82
3.16
3.14
3.13
3.11
3.01
2.98
2.95
2.92
2.64
2.62
2.58
2.56
1.32
1.28
0.98
2.02
2.05
3.13
0.98
0.98
1.00
2.98
3.00
f1 (ppm)

11ac

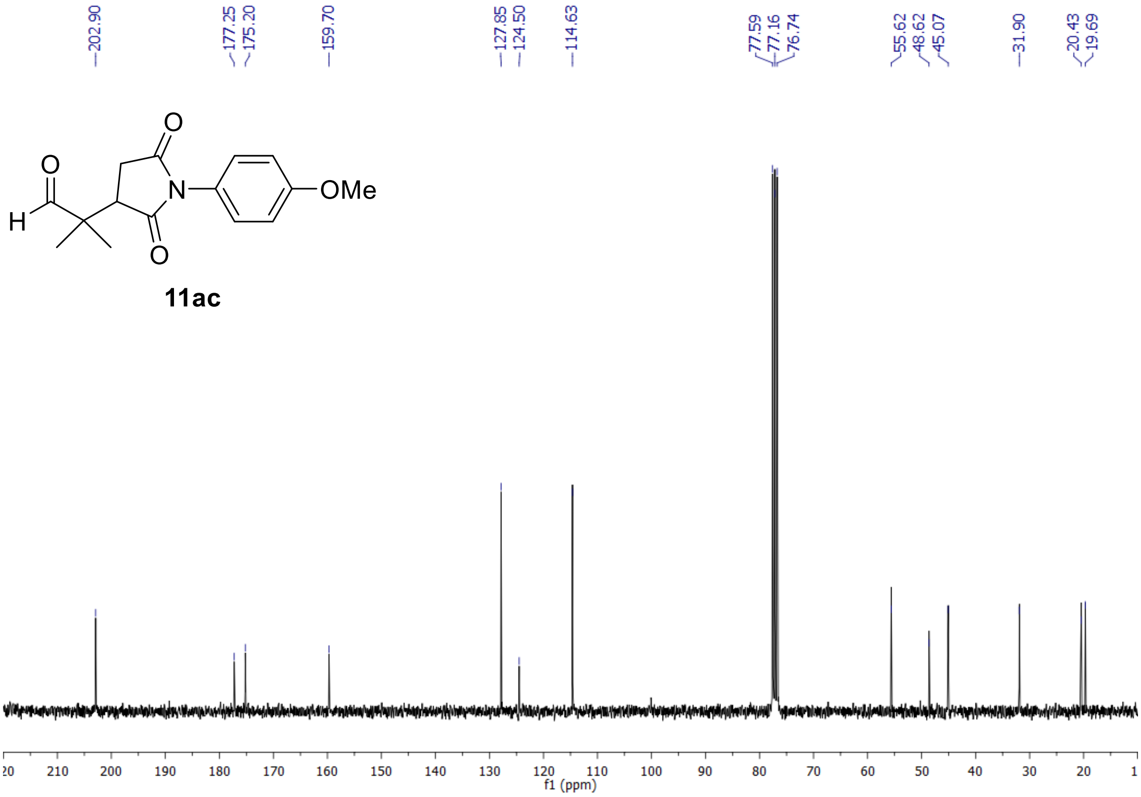

202.90
177.25
175.20
159.70
127.85
124.50
114.63
77.59
77.16
76.74
55.62
48.62
45.07
31.90
20.43
19.69
f1 (ppm)

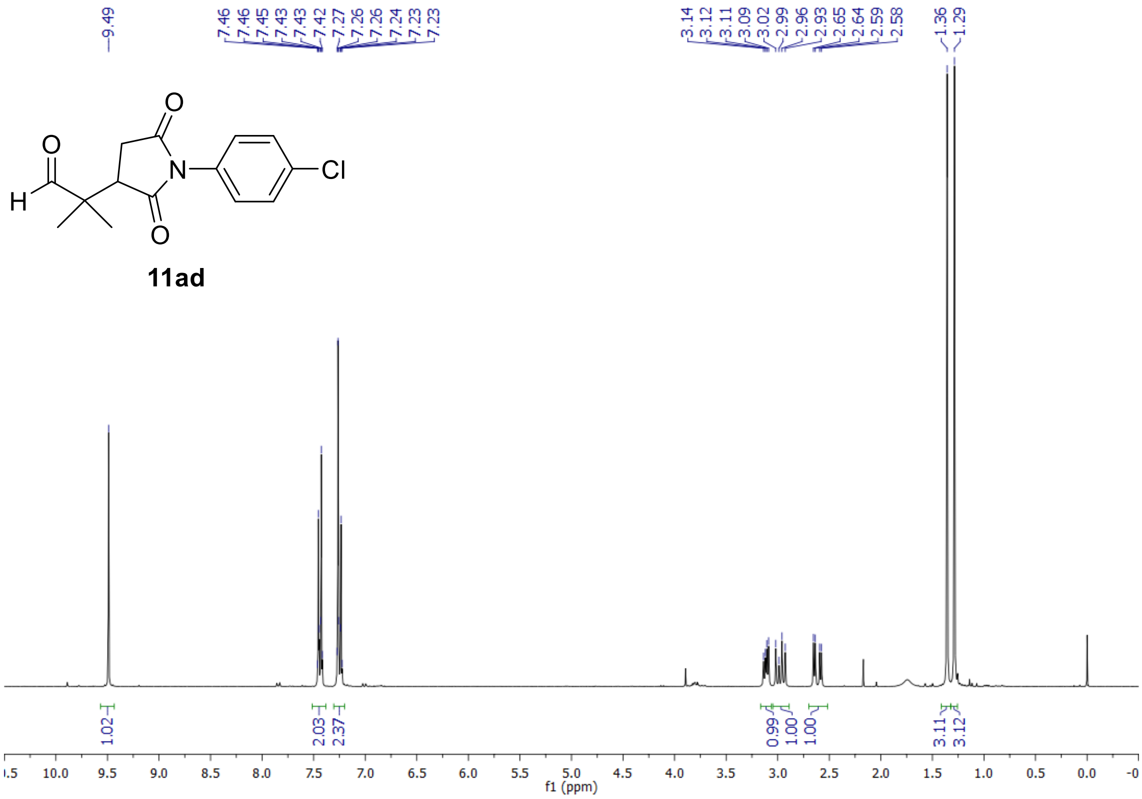
9.49
7.46
7.46
7.45
7.43
7.43
7.42
7.27
7.26
7.26
7.24
7.23
7.23
3.14
3.12
3.11
3.09
3.02
2.99
2.96
2.93
2.65
2.64
2.59
2.58
1.36
1.29
O
O
H
N
Cl
O
11ad
1.02
2.03
2.37
0.99
1.00
1.00
3.11
3.12
10.0 9.5 9.0 8.5 8.0 7.5 7.0 6.5 6.0 5.5 5.0 4.5 4.0 3.5 3.0 2.5 2.0 1.5 1.0 0.5 0.0
f1 (ppm)

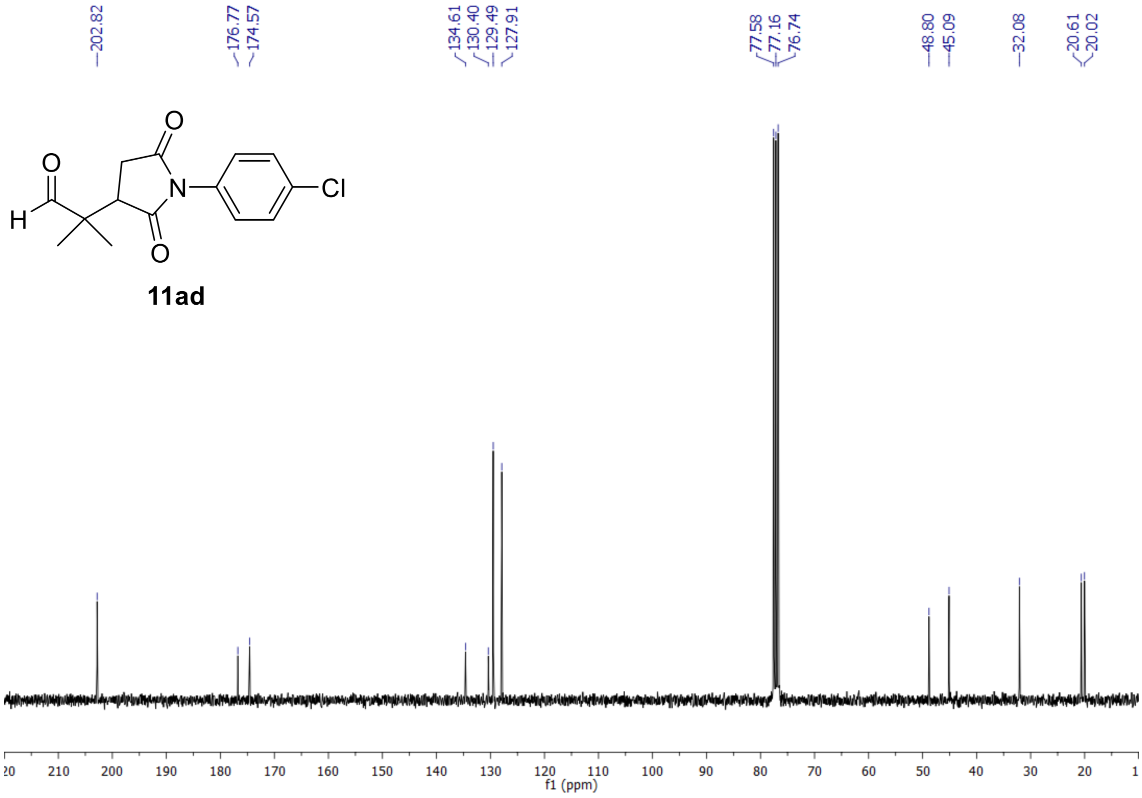
202.82
176.77
174.57
134.61
130.40
129.49
127.91
77.58
77.16
76.74
48.80
45.09
32.08
20.61
20.02
O
O
H
N
Cl
O
11ad
210 200 190 180 170 160 150 140 130 120 110 100 90 80 70 60 50 40 30 20
f1 (ppm)

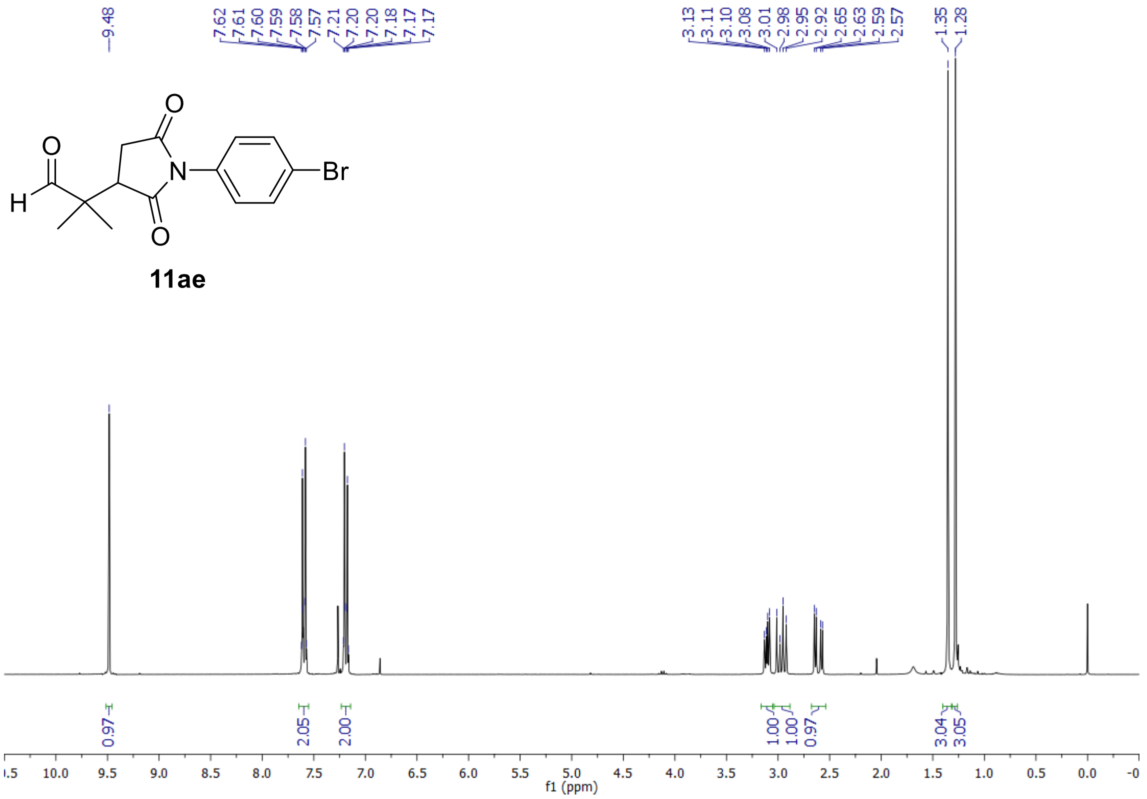
9.48
7.62
7.61
7.60
7.59
7.58
7.57
7.21
7.20
7.20
7.18
7.17
7.17
3.13
3.11
3.10
3.08
3.01
2.98
2.95
2.92
2.65
2.63
2.59
2.57
1.35
1.28
O
O
O
H
N
Br
11ae
0.97
2.05
2.00
1.00
1.00
0.97
3.04
3.05
f1 (ppm)

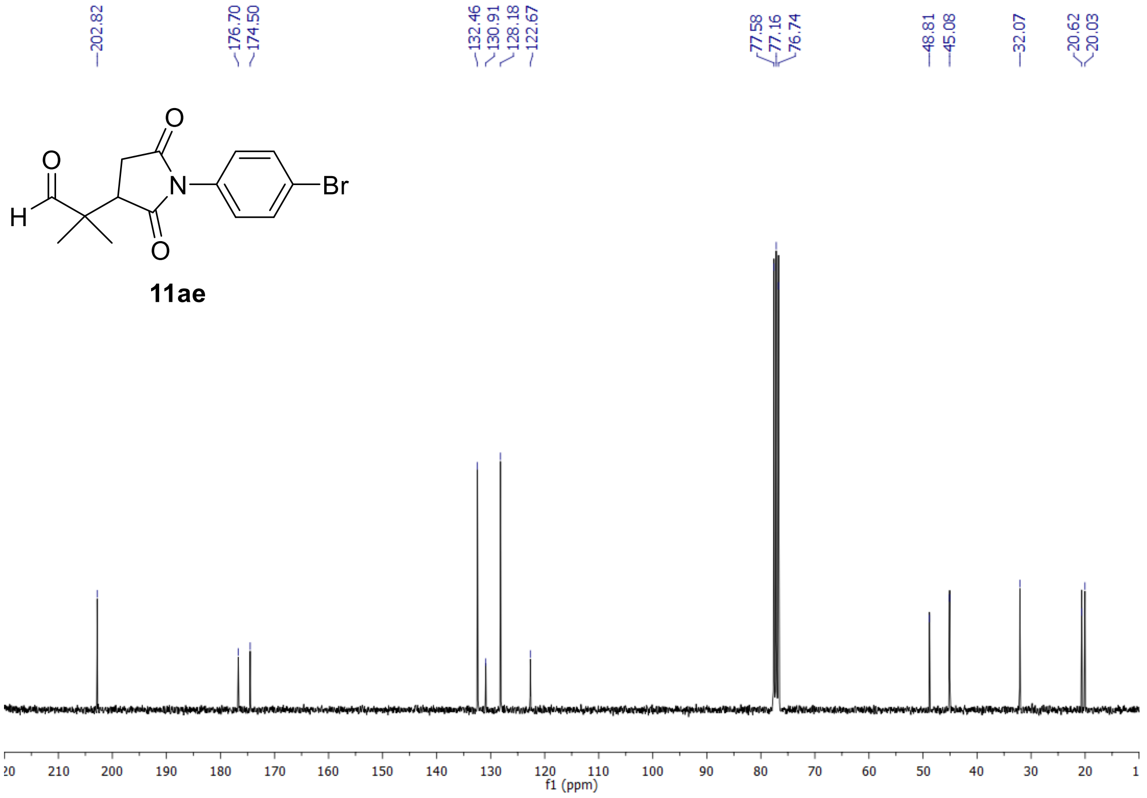
202.82
176.70
174.50
132.46
130.91
128.18
122.67
77.58
77.16
76.74
48.81
45.08
32.07
20.62
20.03
O
O
O
H
N
Br
11ae
f1 (ppm)

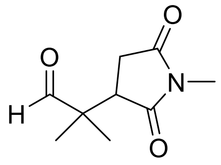

11af

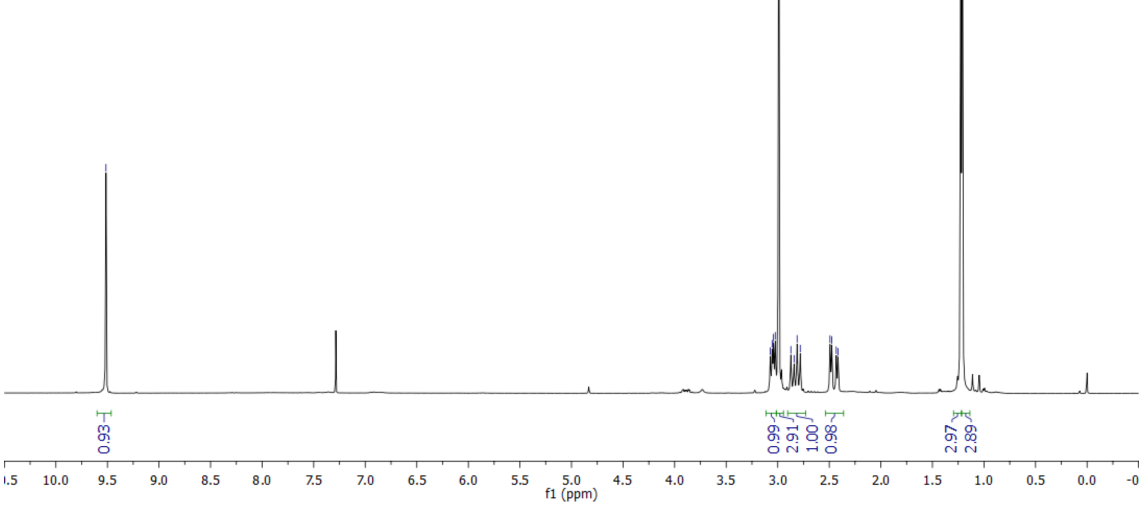

9.51
3.07
3.05
3.04
3.02
2.99
2.87
2.84
2.81
2.78
2.49
2.48
2.43
2.42
1.22
1.21
0.93
0.99
2.91
1.00
0.98
2.97
2.89
f1 (ppm)

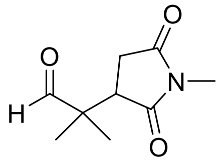

11af

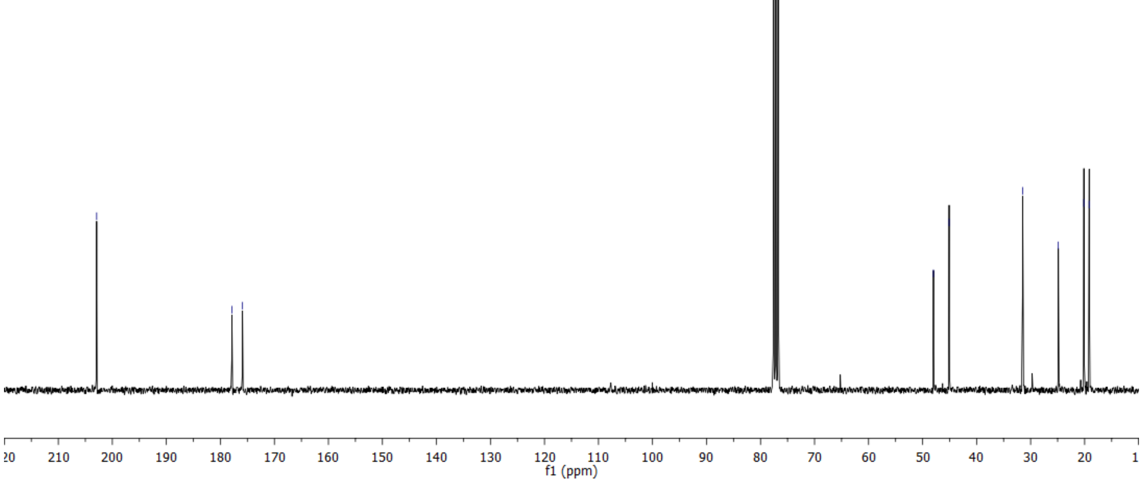

202.90
177.86
175.91
77.58
77.16
76.74
47.99
45.09
31.48
24.88
20.14
19.15
f1 (ppm)

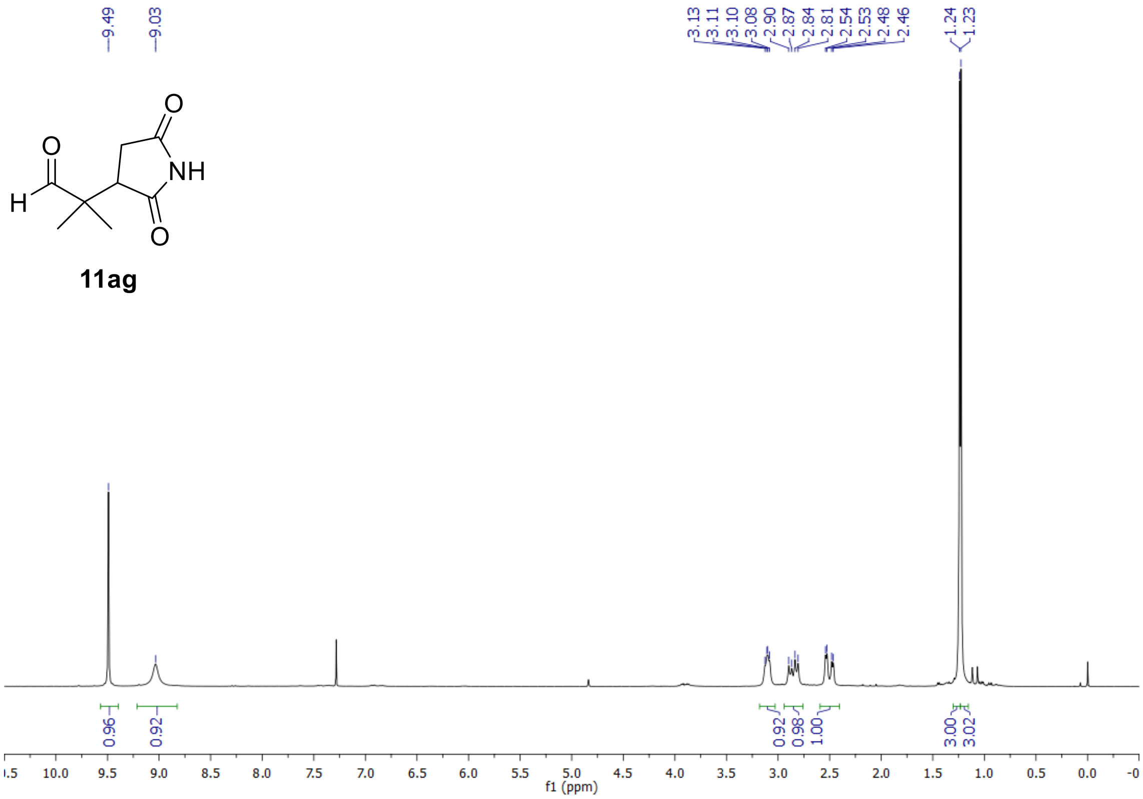
9.49
9.03
3.13
3.11
3.10
3.08
2.90
2.87
2.84
2.81
2.54
2.53
2.48
2.46
1.24
1.23
0.96
0.92
0.92
0.98
1.00
3.00
3.02
f1 (ppm)

11ag

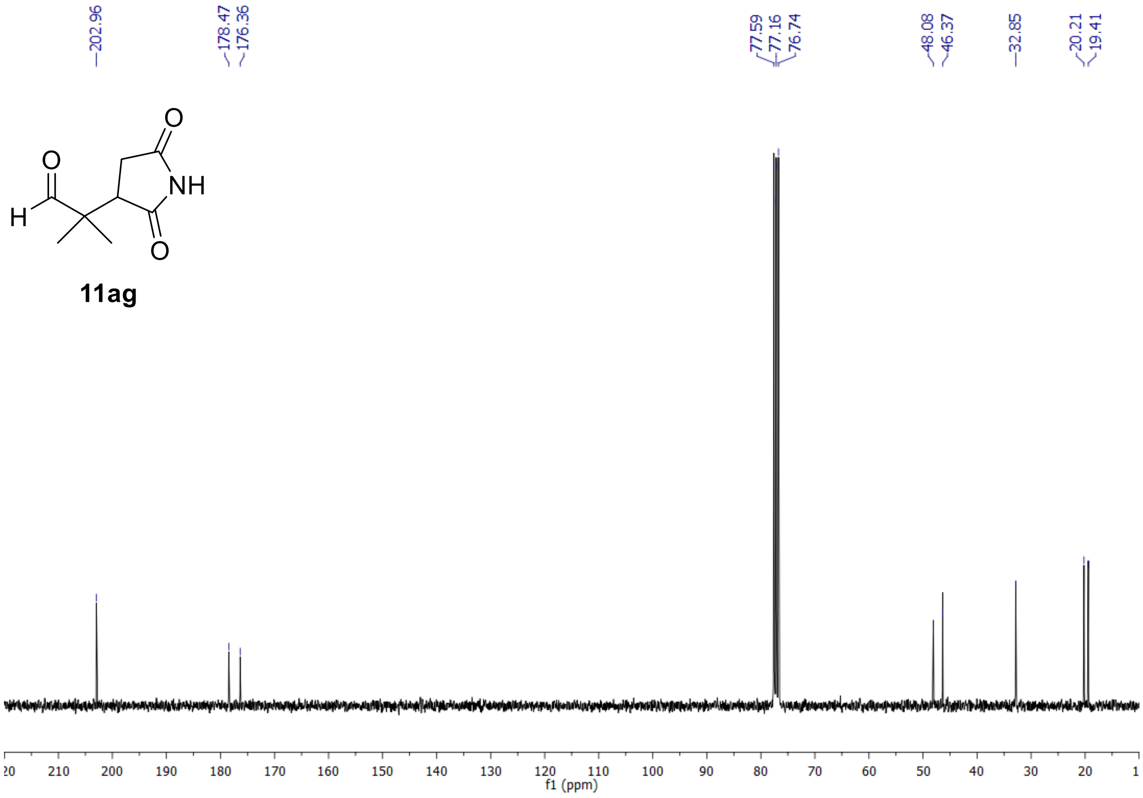
202.96
178.47
176.36
77.59
77.16
76.74
48.08
46.37
32.85
20.21
19.41
f1 (ppm)

11ag

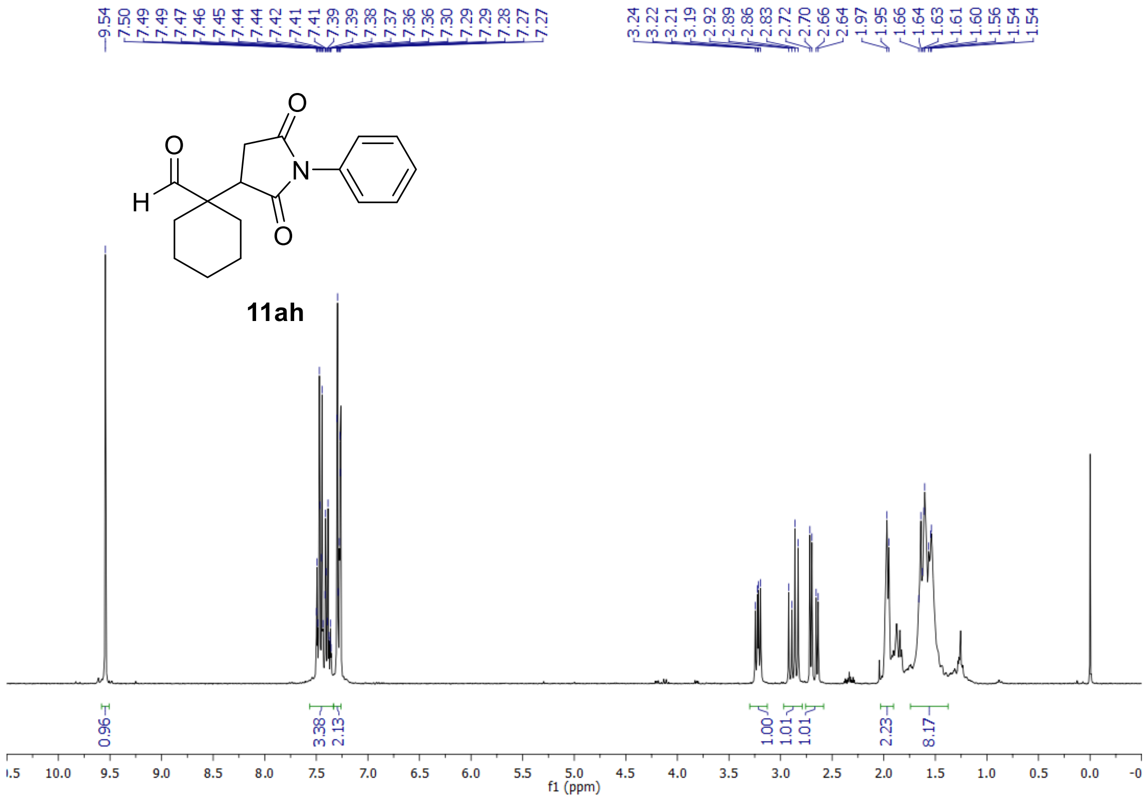
9.54
7.50
7.49
7.49
7.47
7.46
7.45
7.44
7.44
7.42
7.41
7.39
7.39
7.38
7.37
7.36
7.36
7.30
7.29
7.29
7.28
7.27
7.27
3.24
3.22
3.21
3.19
2.92
2.89
2.86
2.83
2.72
2.70
2.66
2.64
1.97
1.95
1.66
1.64
1.63
1.61
1.60
1.56
1.54
1.54
11ah
0.96
3.38
2.13
1.00
1.01
1.01
2.23
8.17
f1 (ppm)

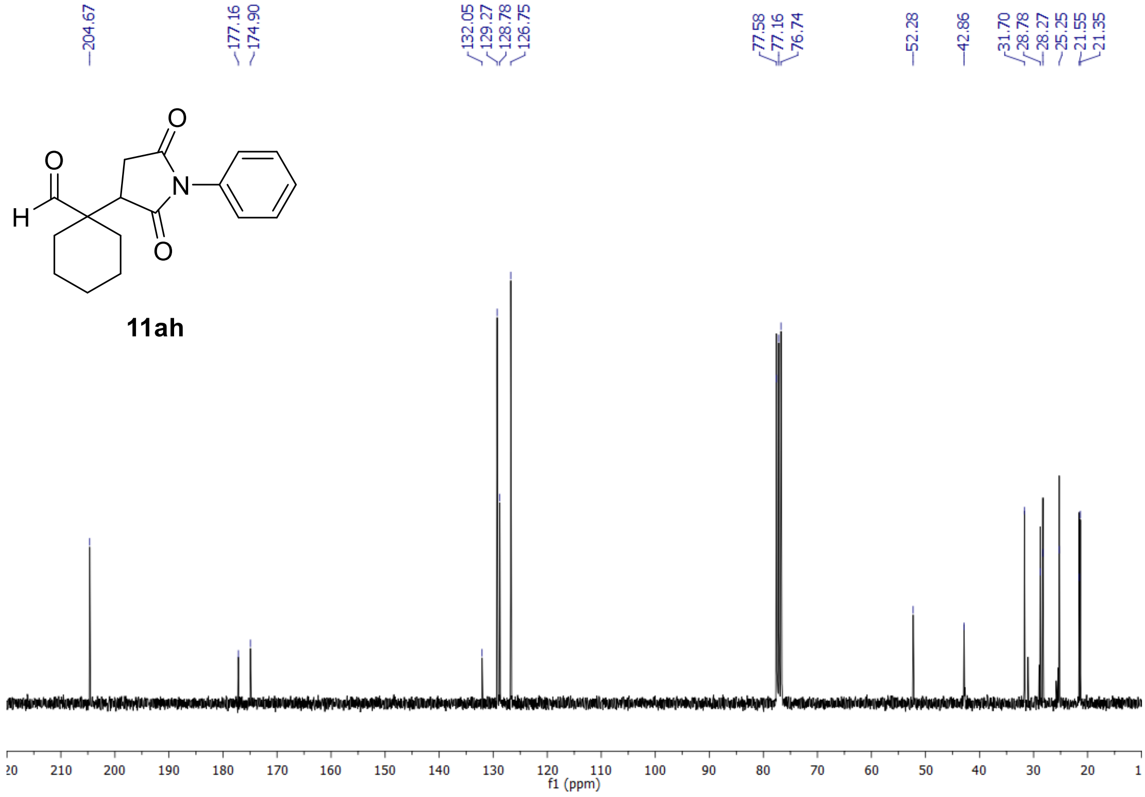
204.67
177.16
174.90
132.05
129.27
128.78
126.75
77.58
77.16
76.74
52.28
42.86
31.70
28.78
28.27
25.25
21.55
21.35
11ah
f1 (ppm)

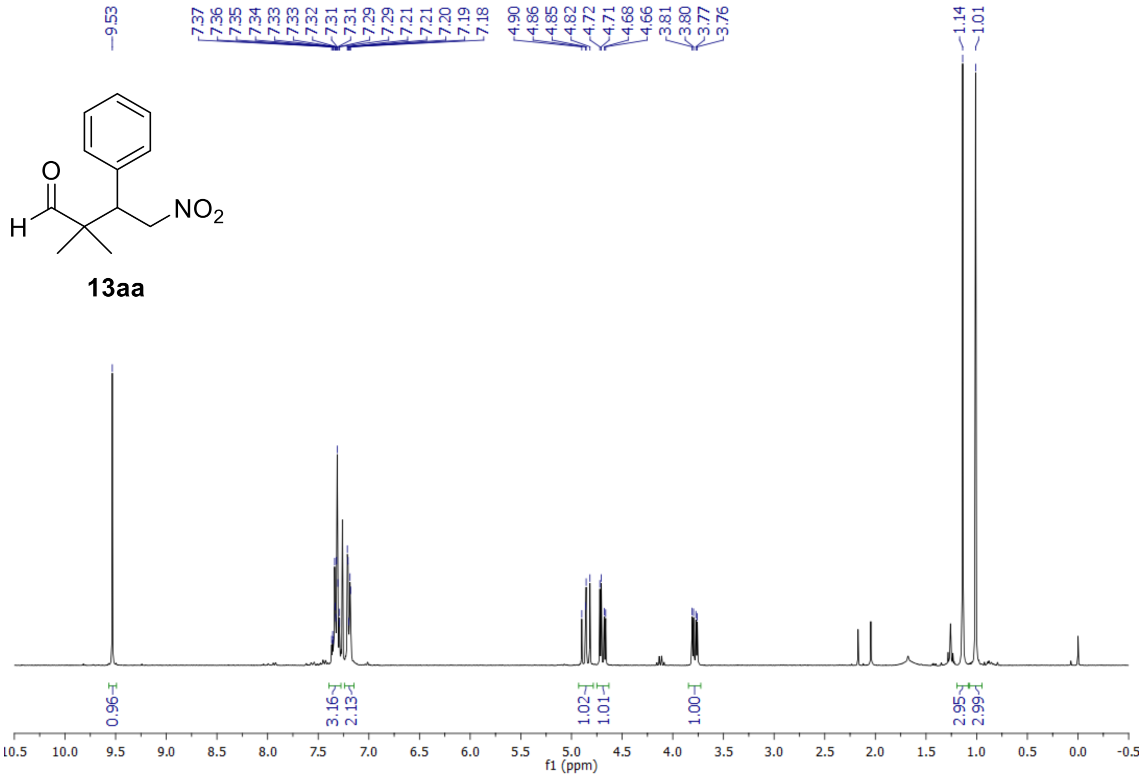

9.53
7.37
7.36
7.35
7.34
7.33
7.33
7.32
7.31
7.31
7.29
7.29
7.21
7.21
7.20
7.19
7.18
4.90
4.86
4.85
4.82
4.72
4.71
4.68
4.66
3.81
3.80
3.77
3.76
1.14
1.01
O
H
NO2
13aa
0.96
3.16
2.13
1.02
1.01
1.00
2.95
2.99
10.5 10.0 9.5 9.0 8.5 8.0 7.5 7.0 6.5 6.0 5.5 5.0 4.5 4.0 3.5 3.0 2.5 2.0 1.5 1.0 0.5 0.0 -0.5
f1 (ppm)

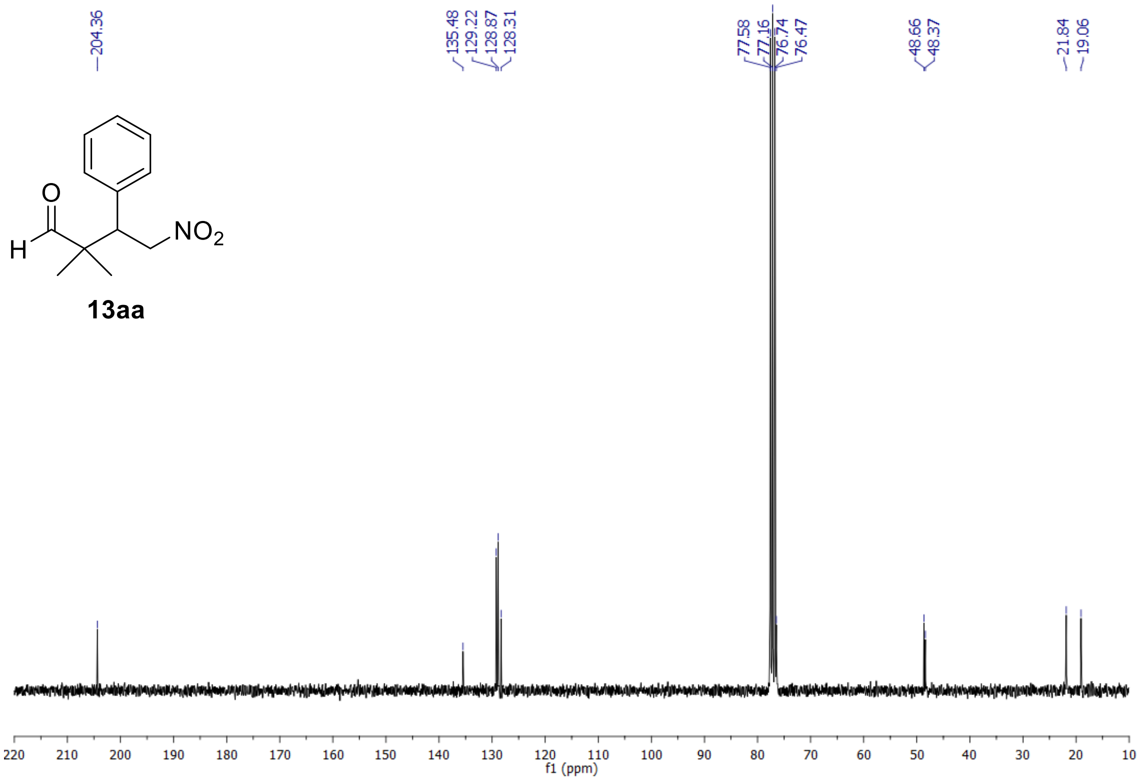

204.36
135.48
129.22
128.87
128.31
77.58
77.16
76.74
76.47
48.66
48.37
21.84
19.06
O
H
NO2
13aa
220 210 200 190 180 170 160 150 140 130 120 110 100 90 80 70 60 50 40 30 20 10
f1 (ppm)

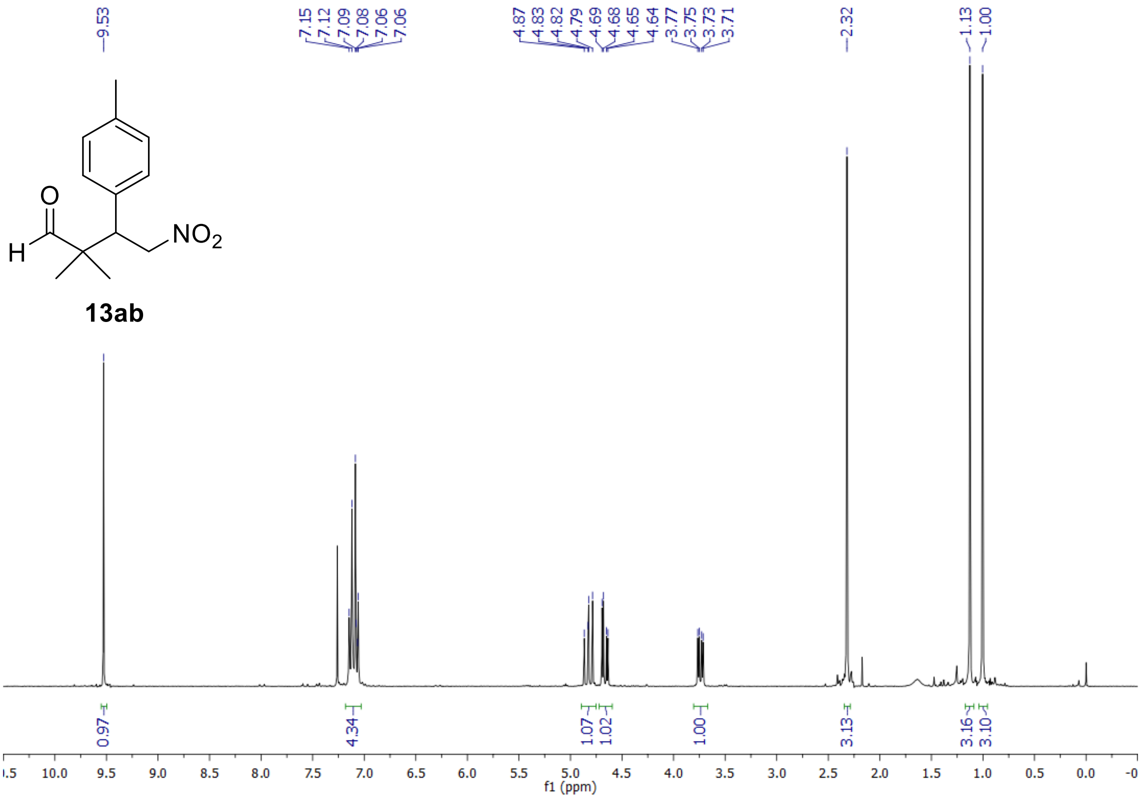
9.53
7.15
7.12
7.09
7.08
7.06
7.06
4.87
4.83
4.82
4.79
4.69
4.68
4.65
4.64
3.77
3.75
3.73
3.71
2.32
1.13
1.00
0.97
4.34
1.07
1.02
1.00
3.13
3.16
3.10
f1 (ppm)

13ab

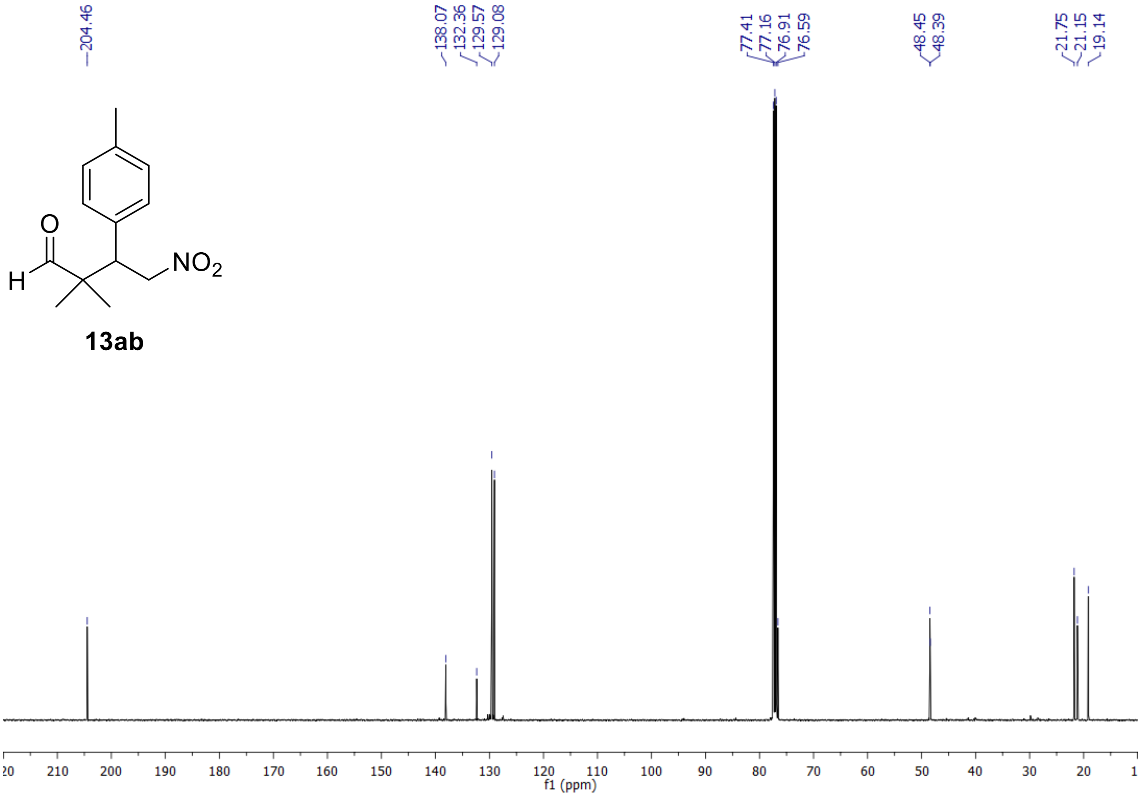
204.46
138.07
132.36
129.57
129.08
77.41
77.16
76.91
76.59
48.45
48.39
21.75
21.15
19.14
f1 (ppm)

13ab

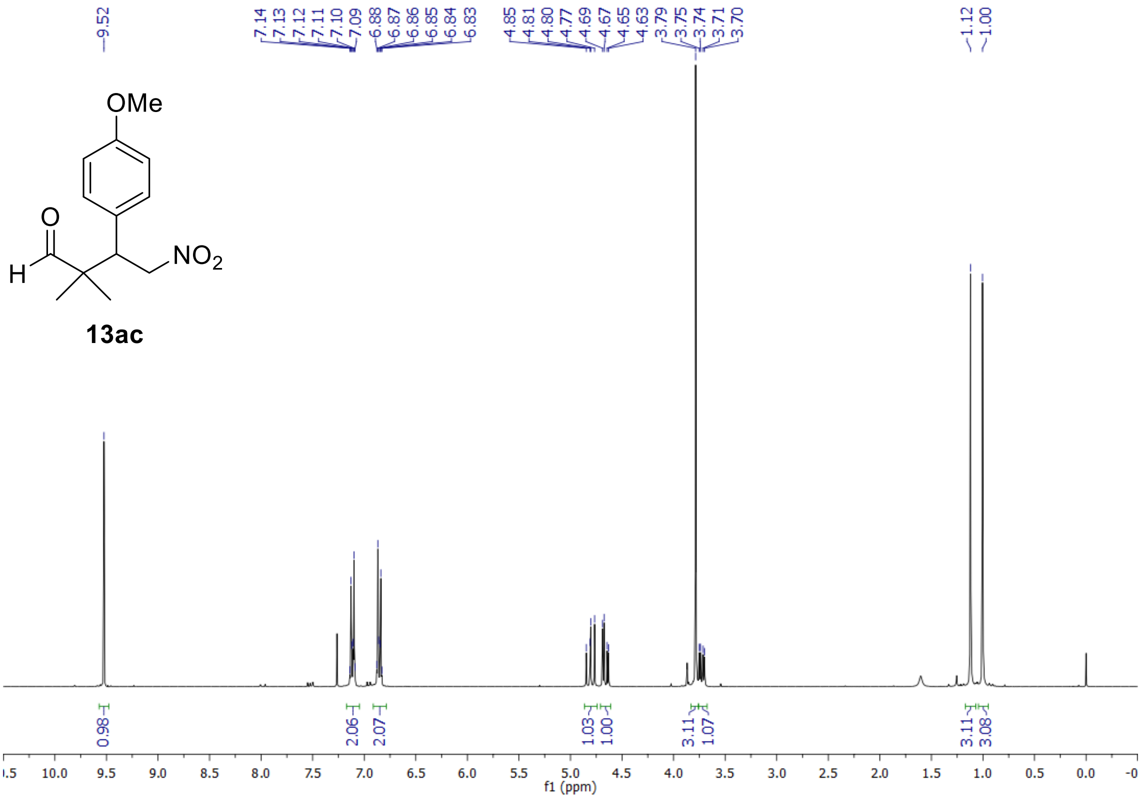

9.52
7.14 7.13 7.12 7.11 7.10 7.09 6.88 6.87 6.86 6.85 6.84 6.83
4.85 4.81 4.80 4.77 4.69 4.67 4.65 4.63 3.79 3.75 3.74 3.71 3.70
1.12 1.00
OMe
O
H
NO2
13ac
0.98
2.06 2.07
1.03 1.00
3.11 1.07
3.11 3.08
10.0 9.5 9.0 8.5 8.0 7.5 7.0 6.5 6.0 5.5 5.0 4.5 4.0 3.5 3.0 2.5 2.0 1.5 1.0 0.5 0.0
f1 (ppm)

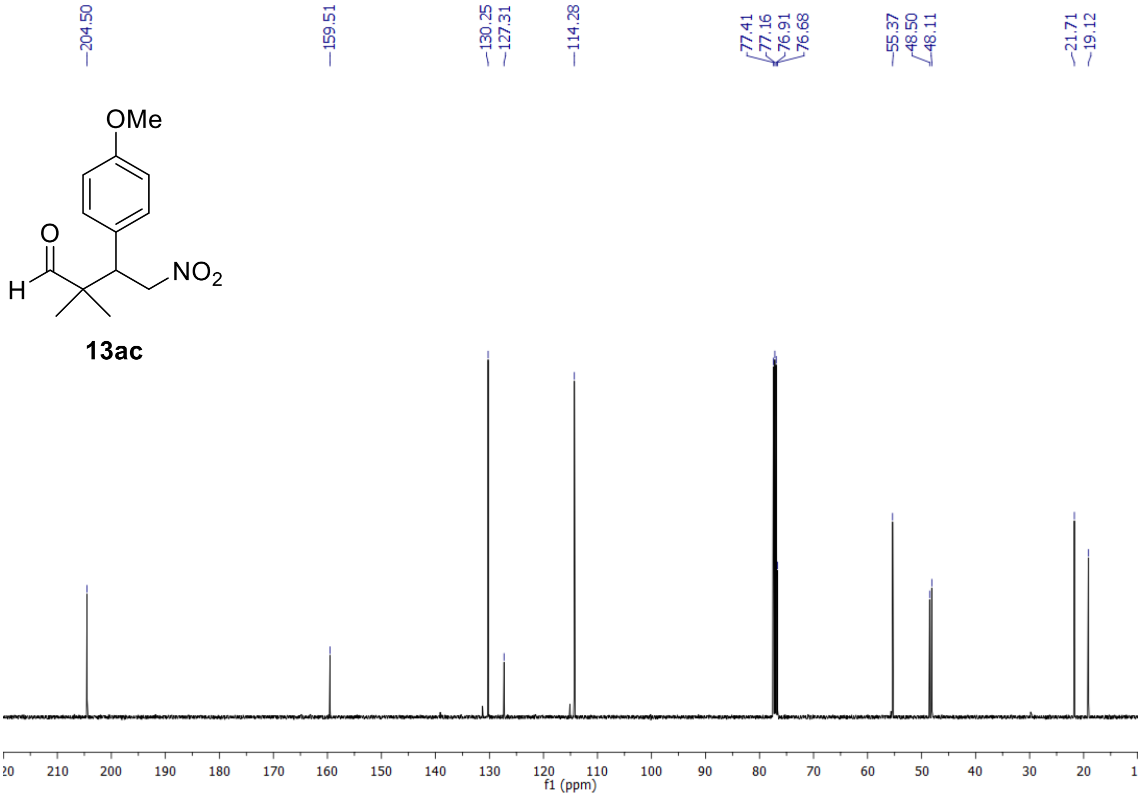

204.50
159.51
130.25 127.31
114.28
77.41 77.16 76.91 76.68
55.37 48.50 48.11
21.71 19.12
OMe
O
H
NO2
13ac
210 200 190 180 170 160 150 140 130 120 110 100 90 80 70 60 50 40 30 20
f1 (ppm)

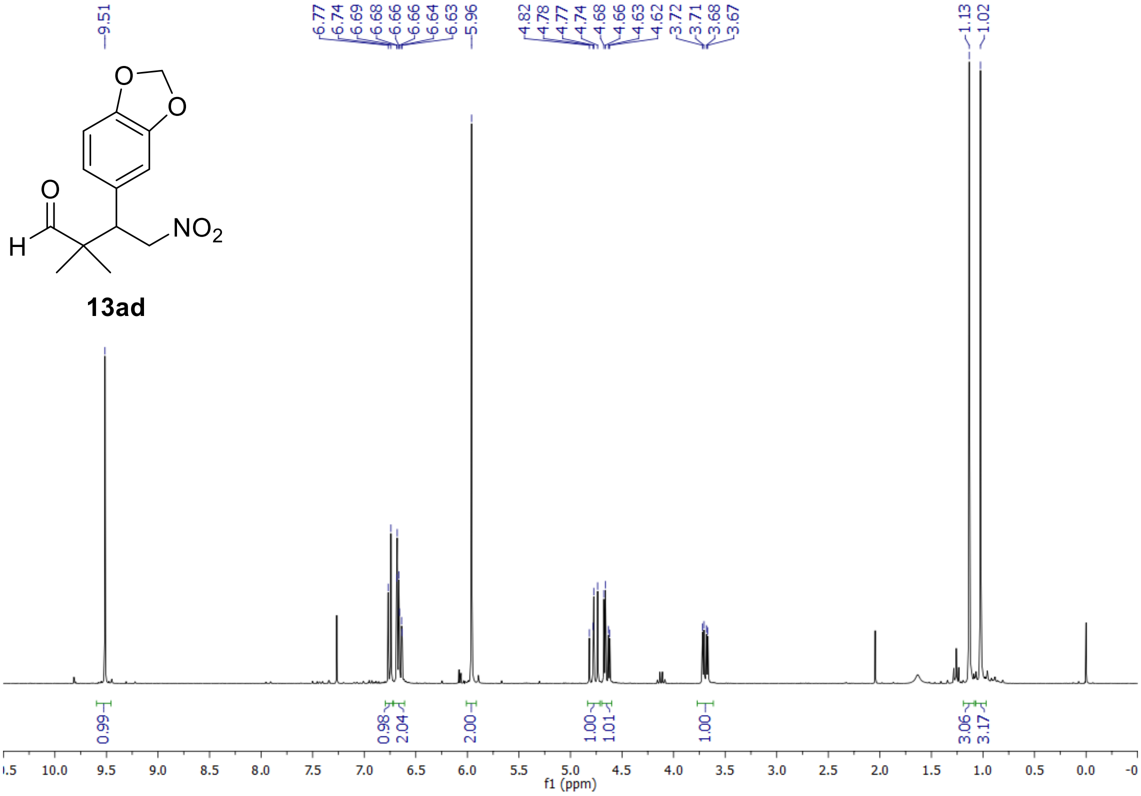
9.51
6.77 6.74 6.69 6.68 6.66 6.66 6.66 6.64 6.63
5.96
4.82 4.78 4.77 4.74 4.68 4.66 4.63 4.62
3.72 3.71 3.68 3.67
1.13 1.02
O
O
O
H
NO_2
0.99
0.98 2.04
2.00
1.00 1.01
1.00
3.06 3.17
10.0 9.5 9.0 8.5 8.0 7.5 7.0 6.5 6.0 5.5 5.0 4.5 4.0 3.5 3.0 2.5 2.0 1.5 1.0 0.5 0.0
f1 (ppm)

13ad

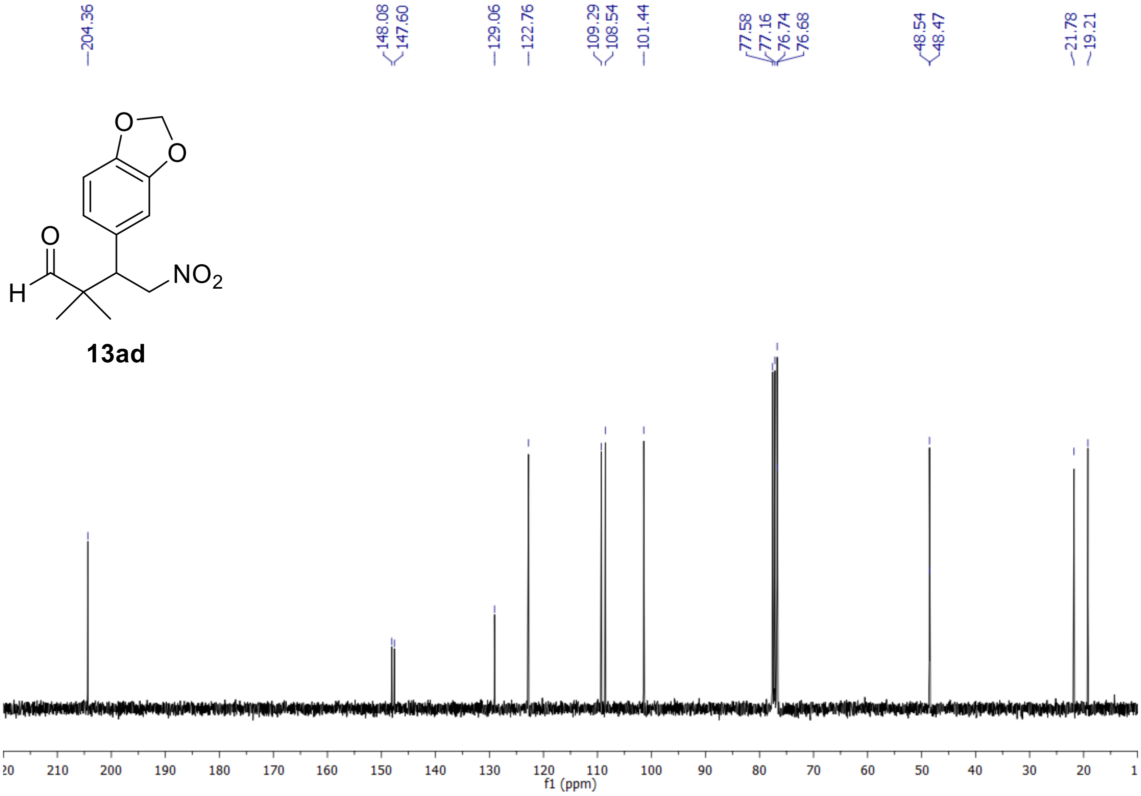
204.36
148.08 147.60
129.06
122.76
109.29 108.54
101.44
77.58 77.16 76.74 76.68
48.54 48.47
21.78 19.21
O
O
O
H
NO_2
210 200 190 180 170 160 150 140 130 120 110 100 90 80 70 60 50 40 30 20
f1 (ppm)

13ad

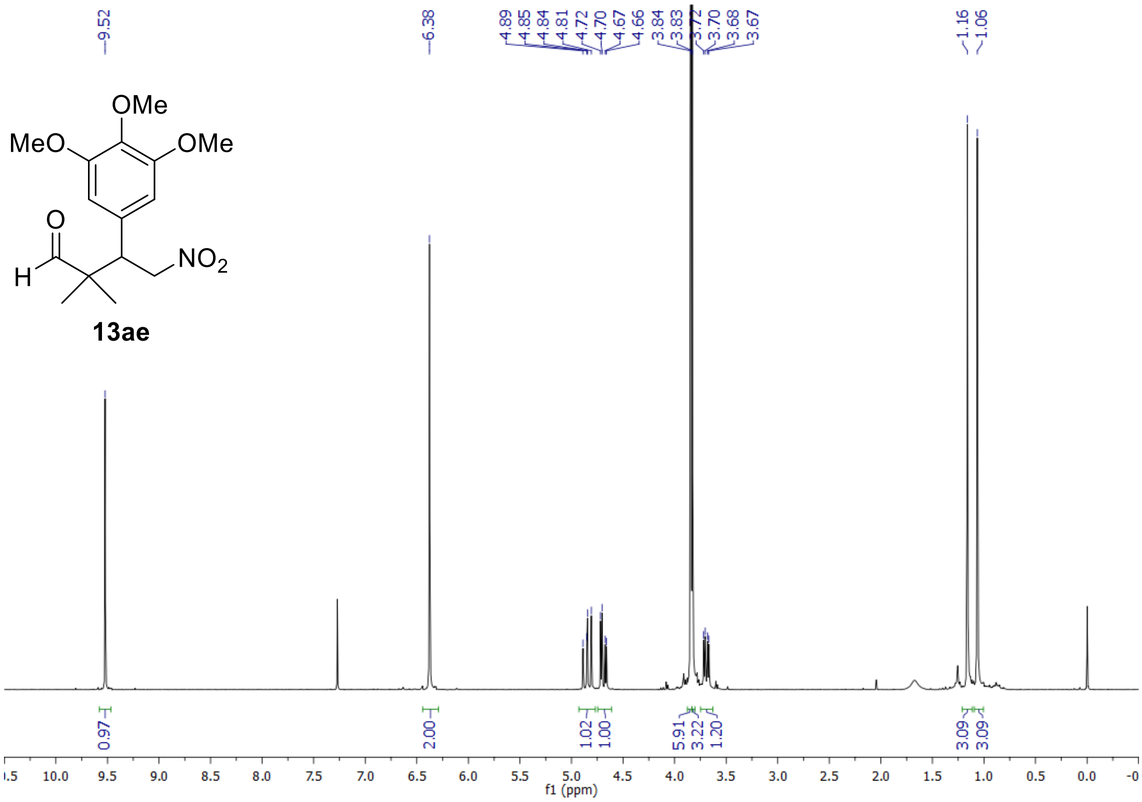

9.52
6.38
4.89
4.85
4.84
4.81
4.72
4.70
4.67
4.66
3.84
3.83
3.72
3.70
3.68
3.67
1.16
1.06
OMe
MeO
OMe
O
H
NO2
0.97
2.00
1.02
1.00
5.91
3.22
1.20
3.09
3.09
f1 (ppm)

13ae

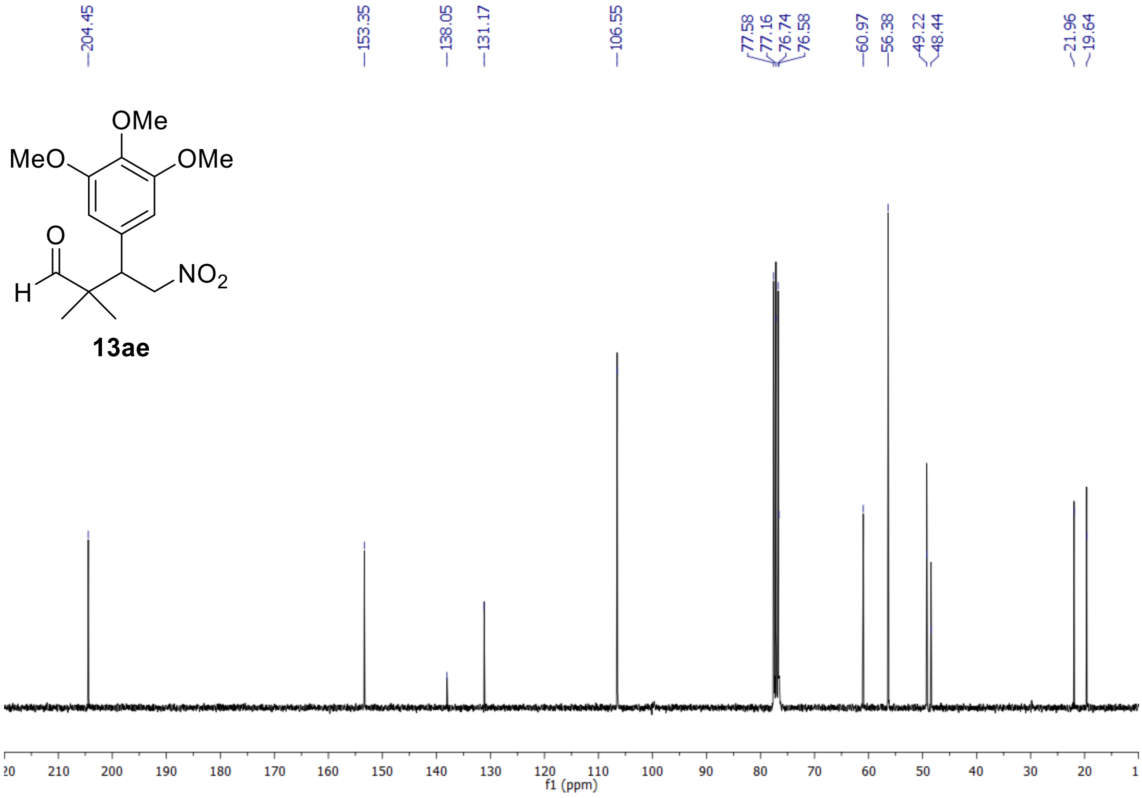

204.45
153.35
138.05
131.17
106.55
77.58
77.16
76.74
76.58
60.97
56.38
49.22
48.44
21.96
19.64
OMe
MeO
OMe
O
H
NO2
f1 (ppm)

13ae

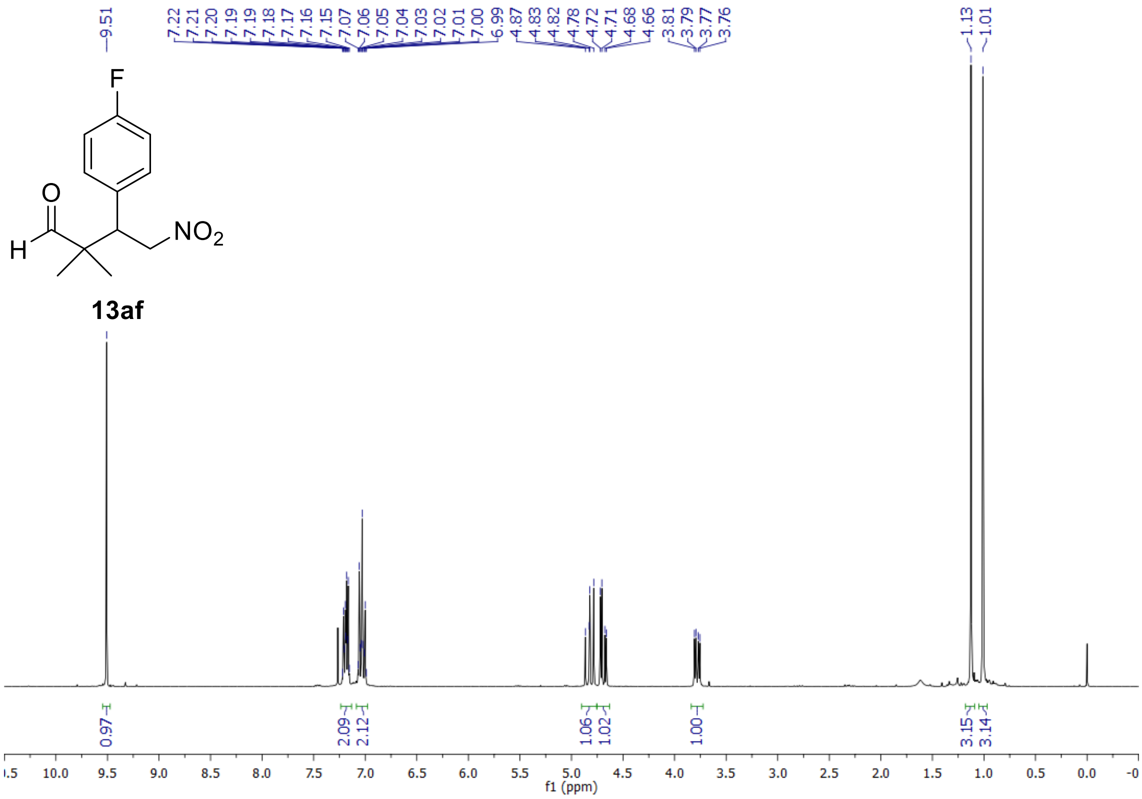
9.51
7.22 7.21 7.20 7.19 7.19 7.18 7.17 7.16 7.15 7.07 7.06 7.05 7.04 7.03 7.02 7.01 7.00 6.99
4.87 4.83 4.82 4.78 4.72 4.71 4.68 4.66
3.81 3.79 3.77 3.76
1.13
1.01
F
O
H
NO2
13af
0.97
2.09
2.12
1.06
1.02
1.00
3.15
3.14
f1 (ppm)

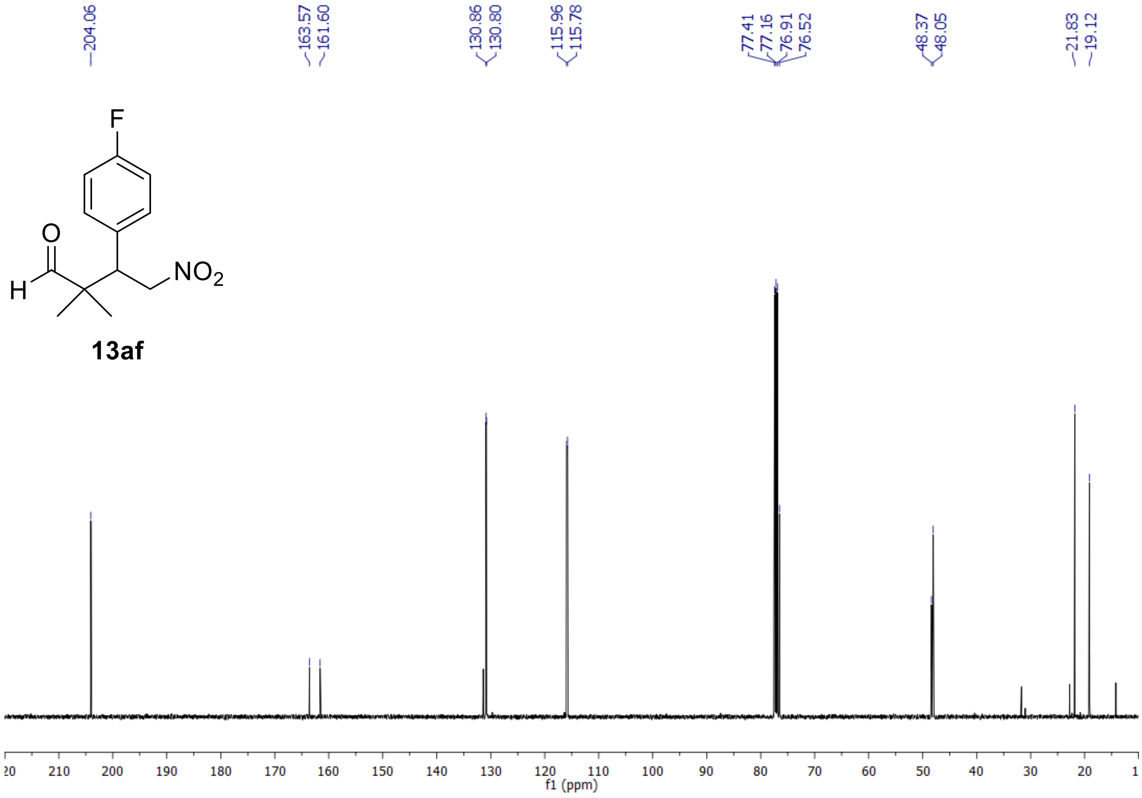
204.06
163.57 161.60
130.86 130.80
115.96 115.78
77.41 77.16 76.91 76.52
48.37 48.05
21.83
19.12
F
O
H
NO2
13af
f1 (ppm)

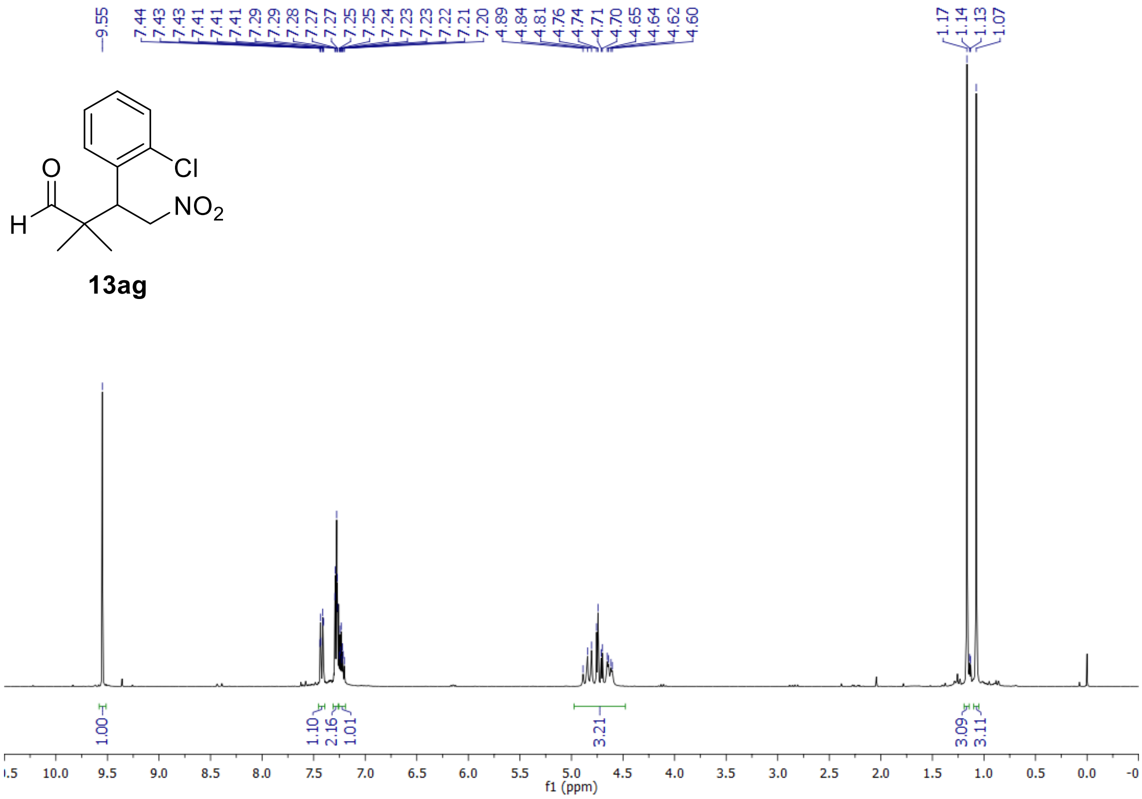
9.55
7.44 7.43 7.43 7.41 7.41 7.41 7.29 7.29 7.28 7.27 7.27 7.25 7.25 7.24 7.23 7.23 7.22 7.21 7.20
4.89 4.84 4.81 4.76 4.74 4.71 4.70 4.65 4.64 4.62 4.60
1.17 1.14 1.13 1.07
O
H
Cl
NO2
13ag
1.00
1.10
2.16
1.01
3.21
3.09
3.11
f1 (ppm)

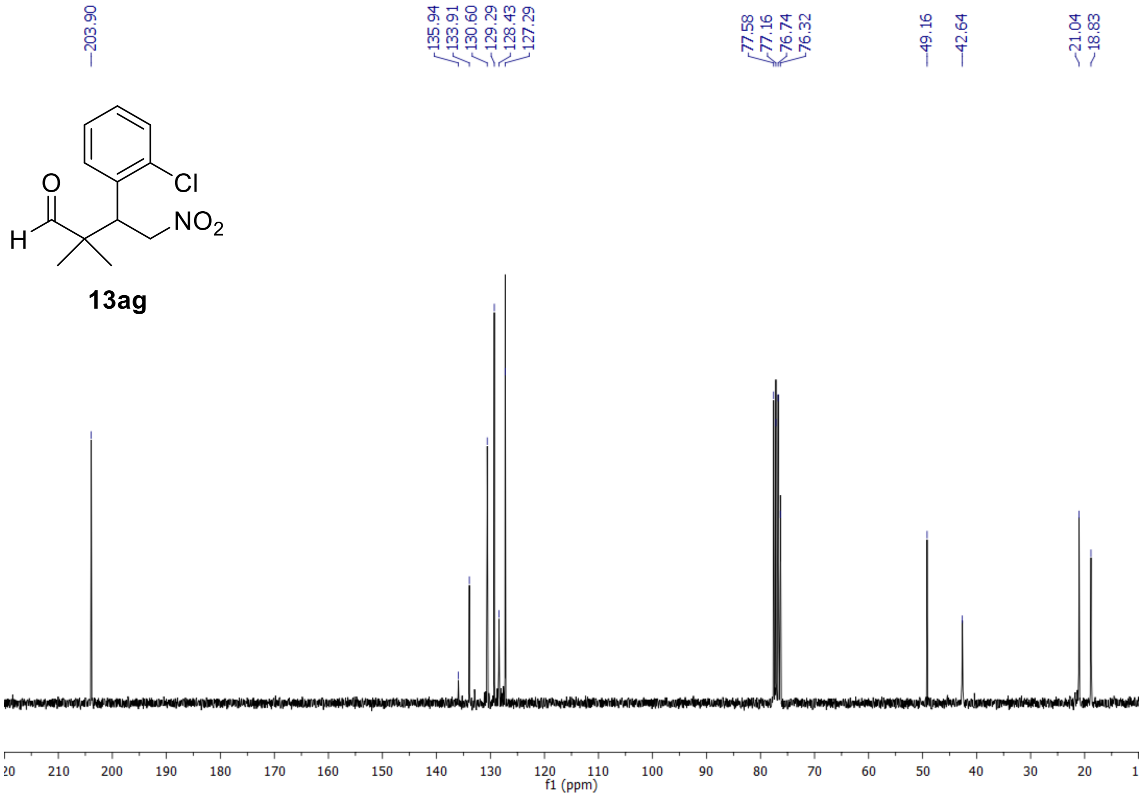
203.90
135.94 133.91 130.60 129.29 128.43 127.29
77.58 77.16 76.74 76.32
49.16
42.64
21.04 18.83
O
H
Cl
NO2
13ag
f1 (ppm)

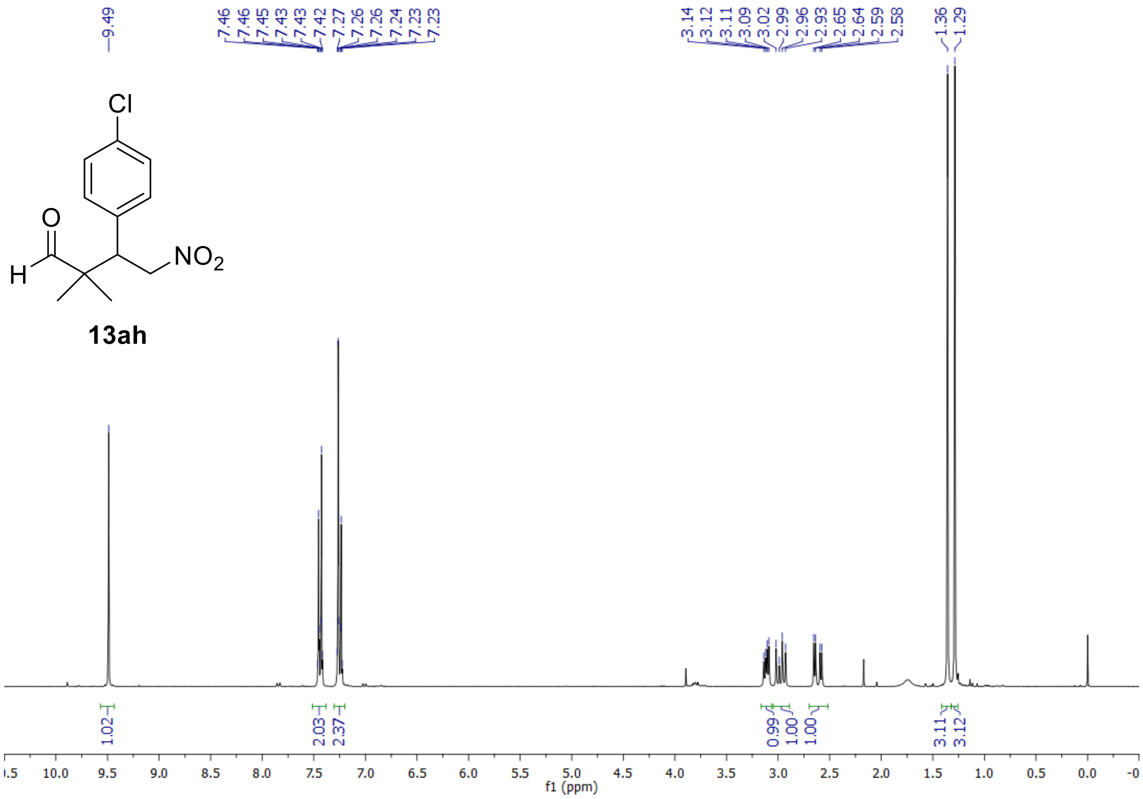

Cl
O
H
NO2
9.49
7.46
7.46
7.45
7.43
7.43
7.42
7.27
7.26
7.26
7.24
7.23
7.23
3.14
3.12
3.11
3.09
3.02
2.99
2.96
2.93
2.65
2.64
2.59
2.58
1.36
1.29
1.02
2.03
2.37
0.99
1.00
1.00
3.11
3.12
f1 (ppm)

13ah

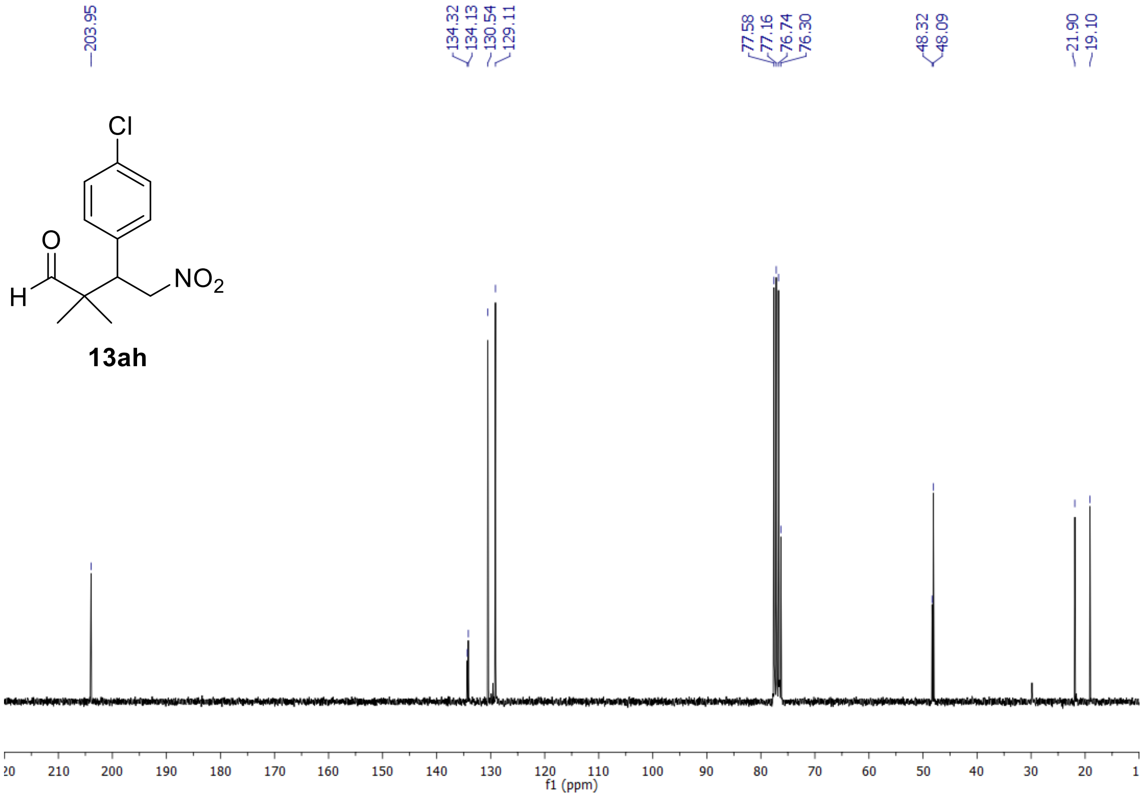

Cl
O
H
NO2
203.95
134.32
134.13
130.54
129.11
77.58
77.16
76.74
76.30
48.32
48.09
21.90
19.10
f1 (ppm)

13ah

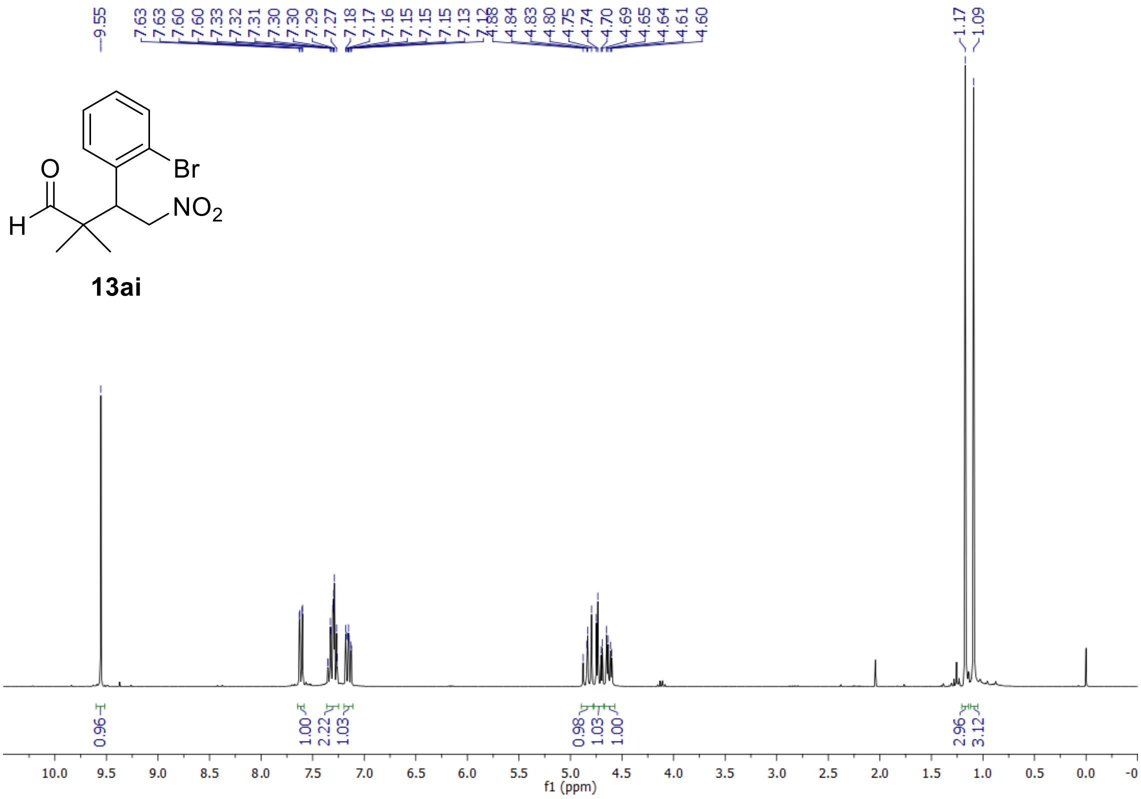

9.55
7.63 7.63 7.60 7.60 7.33 7.32 7.31 7.30 7.30 7.29 7.27 7.18 7.17 7.16 7.15 7.15 7.15 7.13 7.12
4.88 4.84 4.83 4.80 4.75 4.74 4.70 4.69 4.65 4.64 4.61 4.60
1.17 1.09
O
H
Br
NO_2
13ai
0.96 1.00 2.22 1.03 0.98 1.03 1.00 2.96 3.12
f1 (ppm)

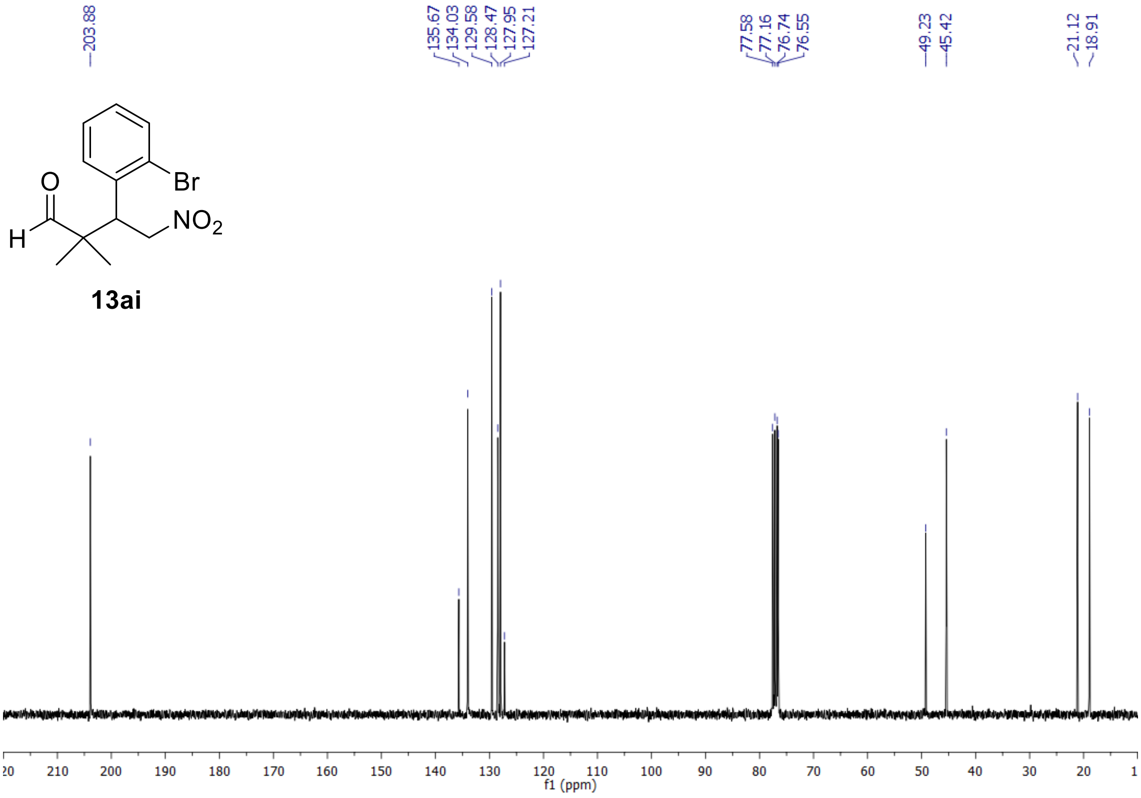

203.88
135.67 134.03 129.58 128.47 127.95 127.21
77.58 77.16 76.74 76.55
49.23 45.42
21.12 18.91
O
H
Br
NO_2
13ai
f1 (ppm)

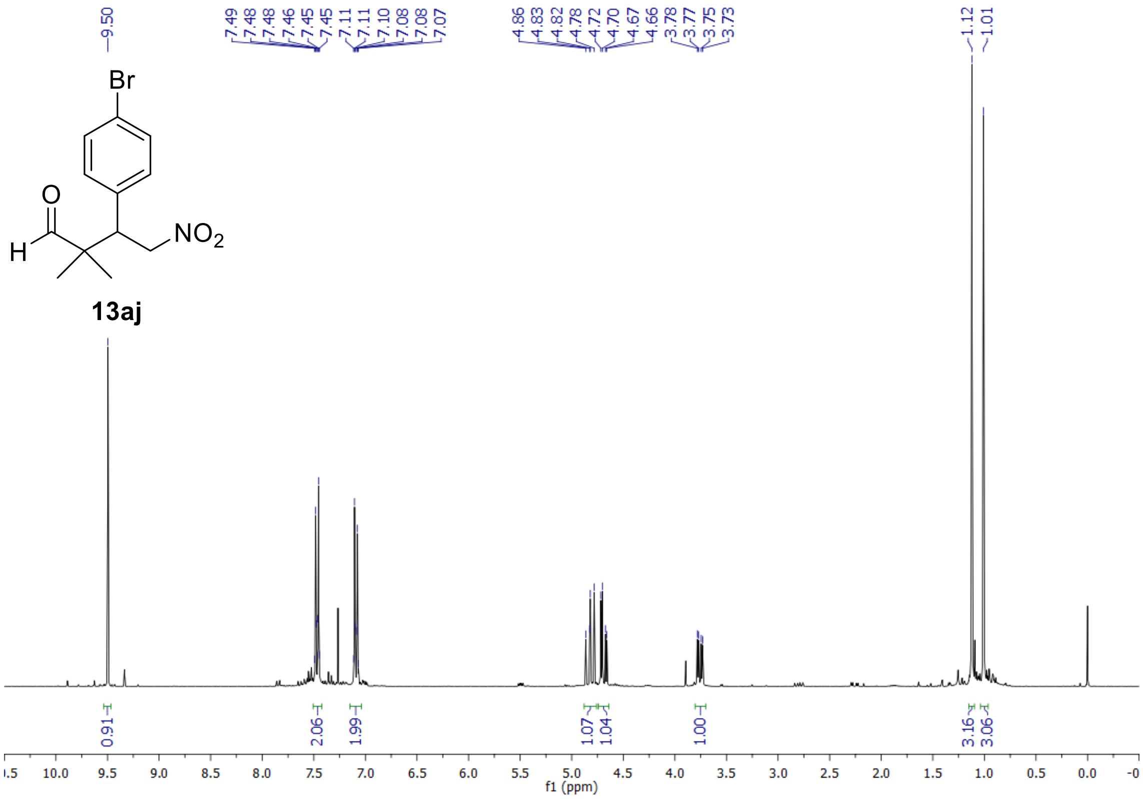

Br
O
H
NO2
13aj
9.50
7.49
7.48
7.48
7.46
7.45
7.45
7.11
7.11
7.10
7.08
7.08
7.07
4.86
4.83
4.82
4.78
4.72
4.70
4.67
4.66
3.78
3.77
3.75
3.73
1.12
1.01
0.91
2.06
1.99
1.07
1.04
1.00
3.16
3.06
f1 (ppm)

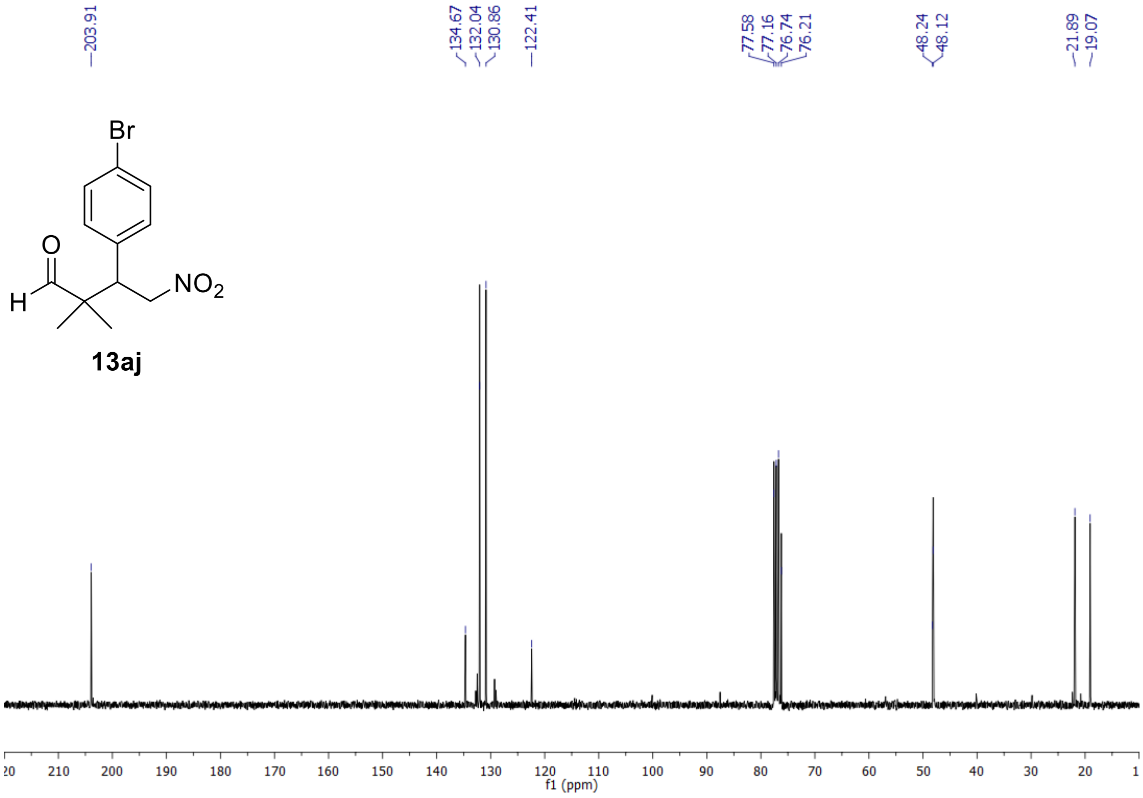

Br
O
H
NO2
13aj
203.91
134.67
132.04
130.86
122.41
77.58
77.16
76.74
76.21
48.24
48.12
21.89
19.07
f1 (ppm)

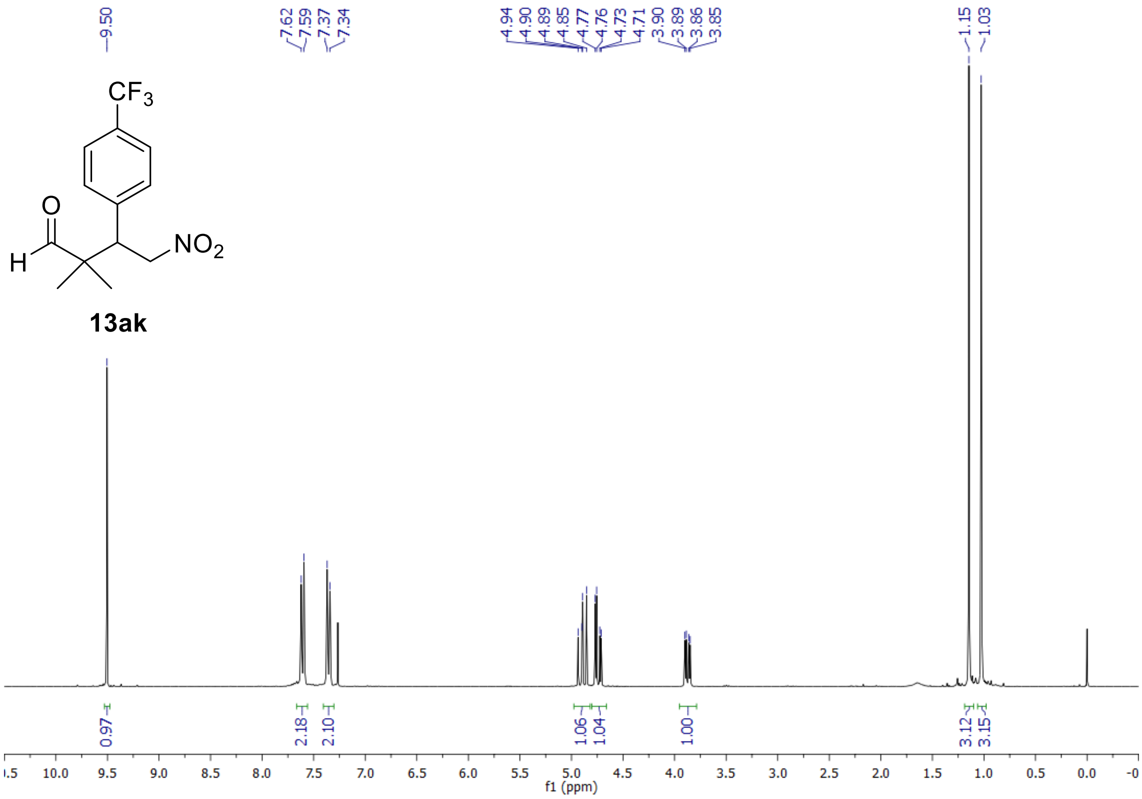
9.50
7.62
7.59
7.37
7.34
4.94
4.90
4.89
4.85
4.77
4.76
4.73
4.71
3.90
3.89
3.86
3.85
1.15
1.03
CF3
O
H
NO2
13ak
0.97
2.18
2.10
1.06
1.04
1.00
3.12
3.15
f1 (ppm)

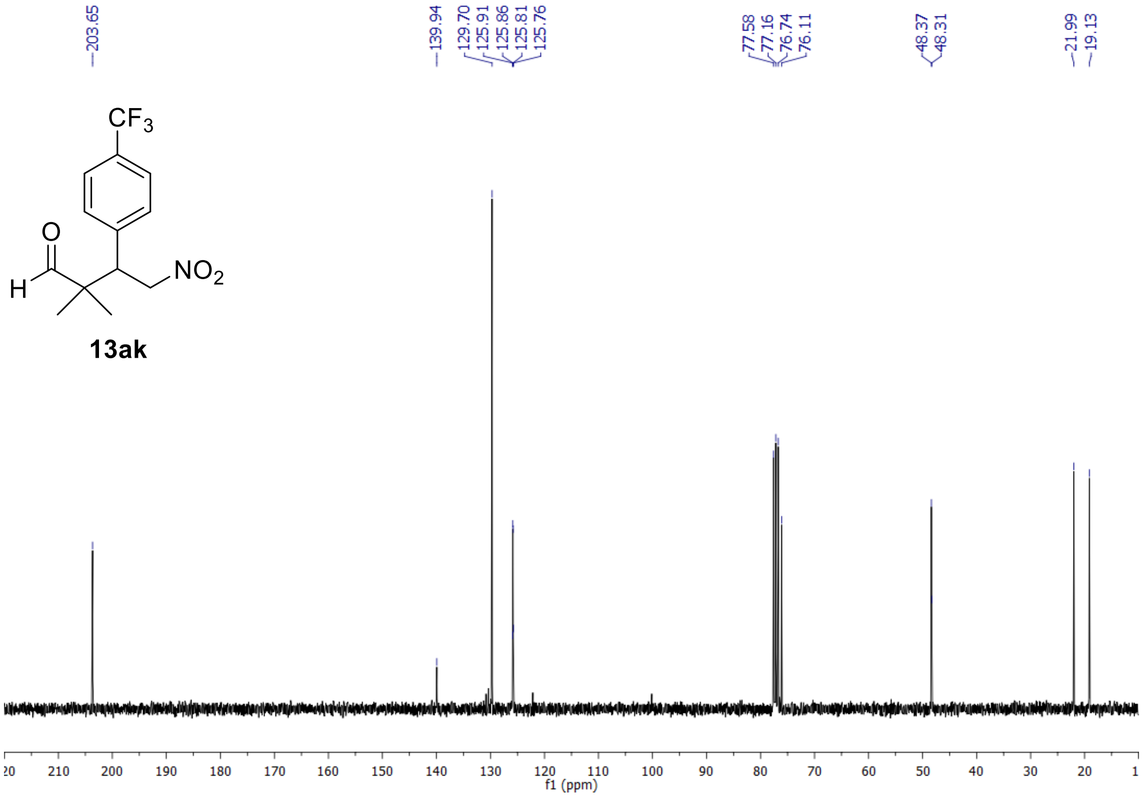
203.65
139.94
129.70
125.91
125.86
125.81
125.76
77.58
77.16
76.74
76.11
48.37
48.31
21.99
19.13
CF3
O
H
NO2
13ak
f1 (ppm)

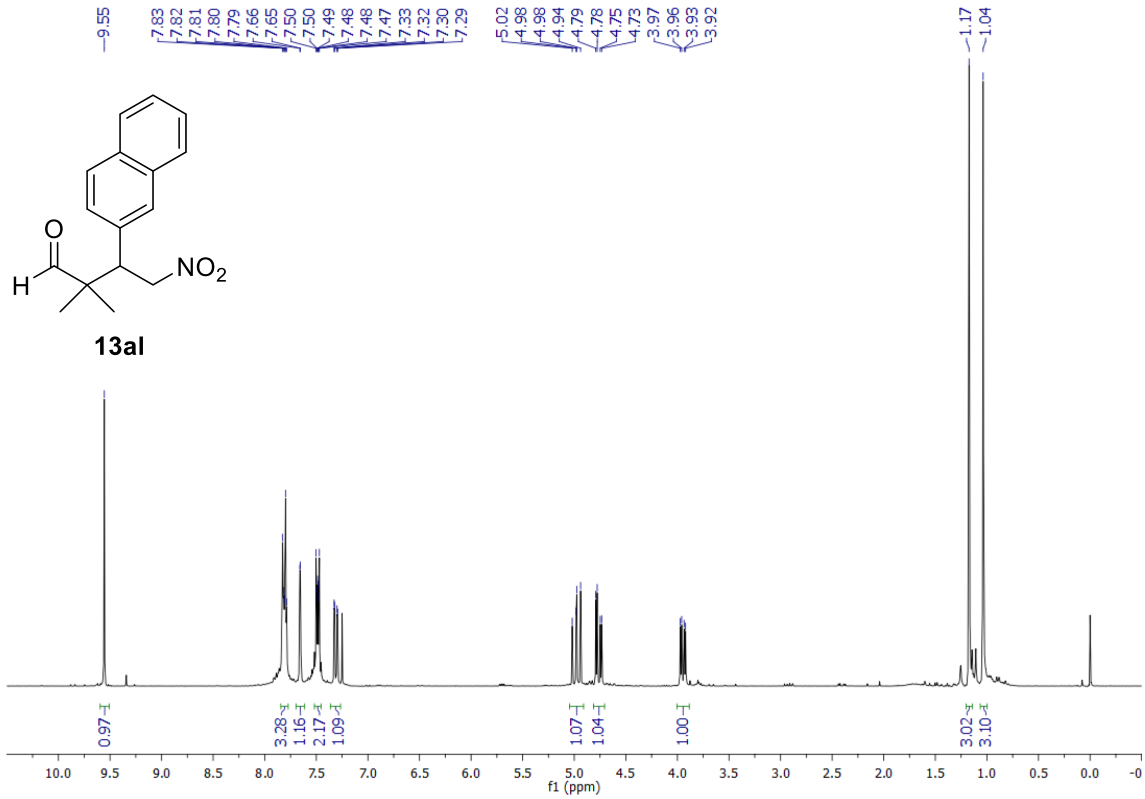

9.55
7.83 7.82 7.81 7.80 7.79 7.66 7.65 7.50 7.50 7.49 7.48 7.48 7.47 7.33 7.32 7.30 7.29
5.02 4.98 4.98 4.94 4.79 4.78 4.75 4.73 3.97 3.96 3.93 3.92
1.17 1.04
NO_2
H
O
13al
0.97 3.28 1.16 2.17 1.09 1.07 1.04 1.00 3.02 3.10
f1 (ppm)

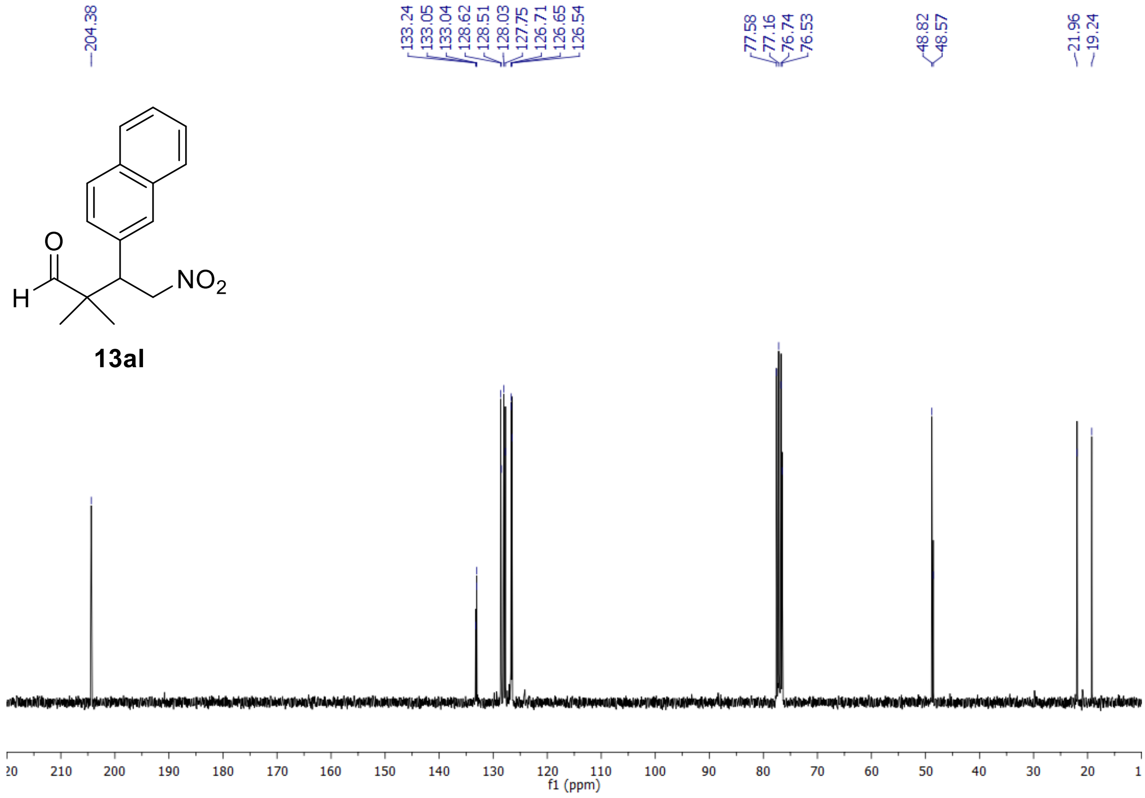

204.38
133.24 133.05 133.04 128.62 128.51 128.03 127.75 126.71 126.65 126.54
77.58 77.16 76.74 76.53
48.82 48.57
21.96 19.24
NO_2
H
O
13al
f1 (ppm)

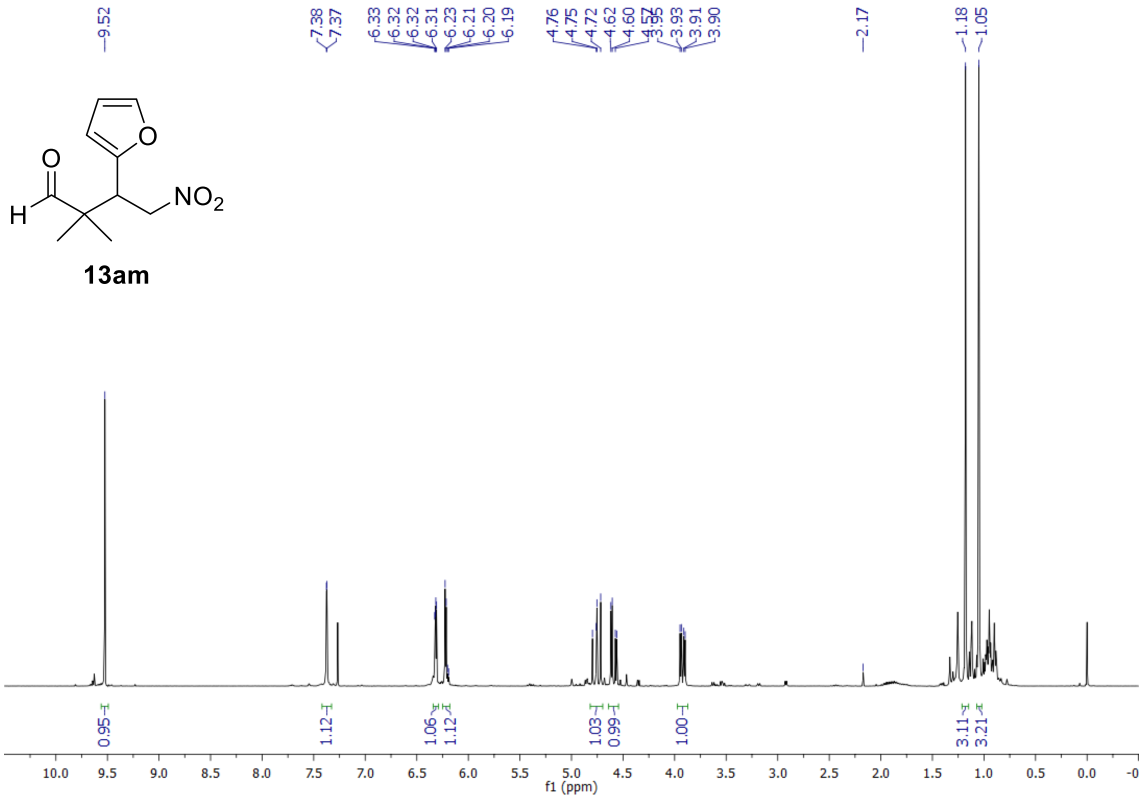
9.52
7.38
7.37
6.33
6.32
6.32
6.31
6.23
6.21
6.20
6.19
4.76
4.75
4.72
4.62
4.60
4.57
3.95
3.93
3.91
3.90
2.17
1.18
1.05
O
H
NO2
13am
0.95
1.12
1.06
1.12
1.03
0.99
1.00
3.11
3.21
10.0 9.5 9.0 8.5 8.0 7.5 7.0 6.5 6.0 5.5 5.0 4.5 4.0 3.5 3.0 2.5 2.0 1.5 1.0 0.5 0.0
f1 (ppm)

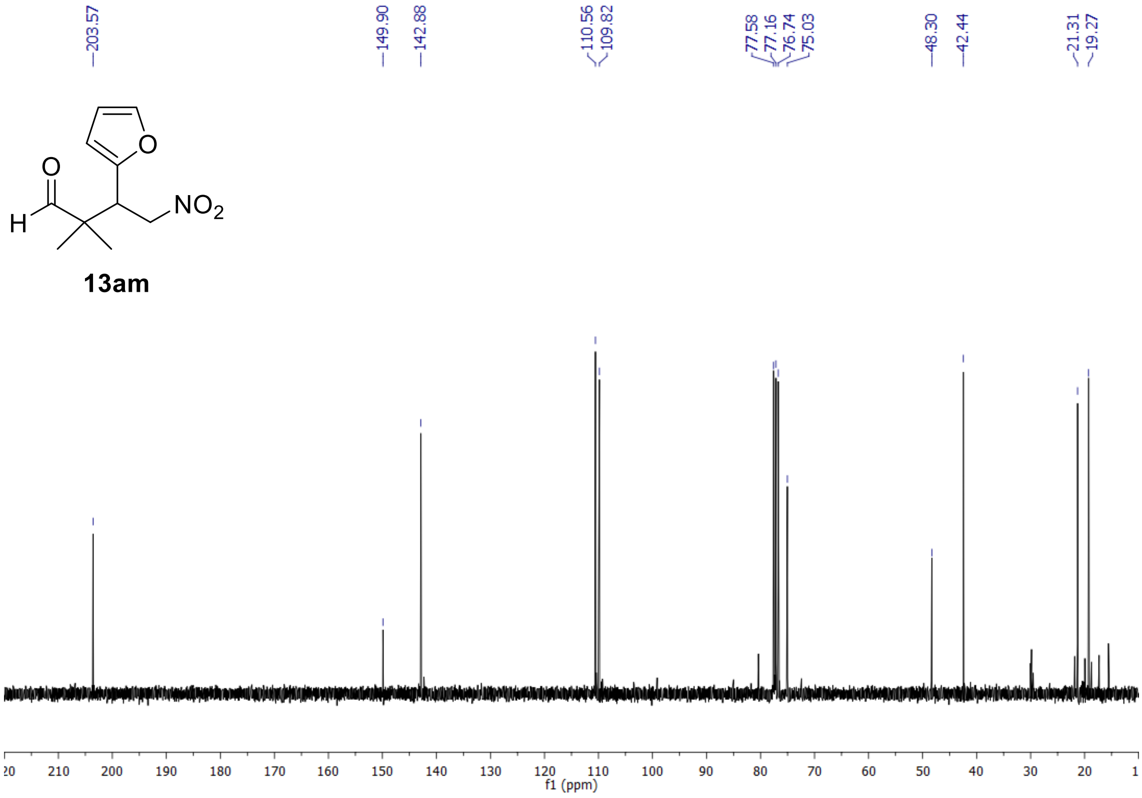
203.57
149.90
142.88
110.56
109.82
77.58
77.16
76.74
75.03
48.30
42.44
21.31
19.27
O
H
NO2
13am
210 200 190 180 170 160 150 140 130 120 110 100 90 80 70 60 50 40 30 20
f1 (ppm)

HPLC Chromatograms

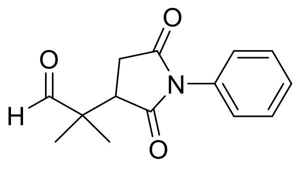

11aa

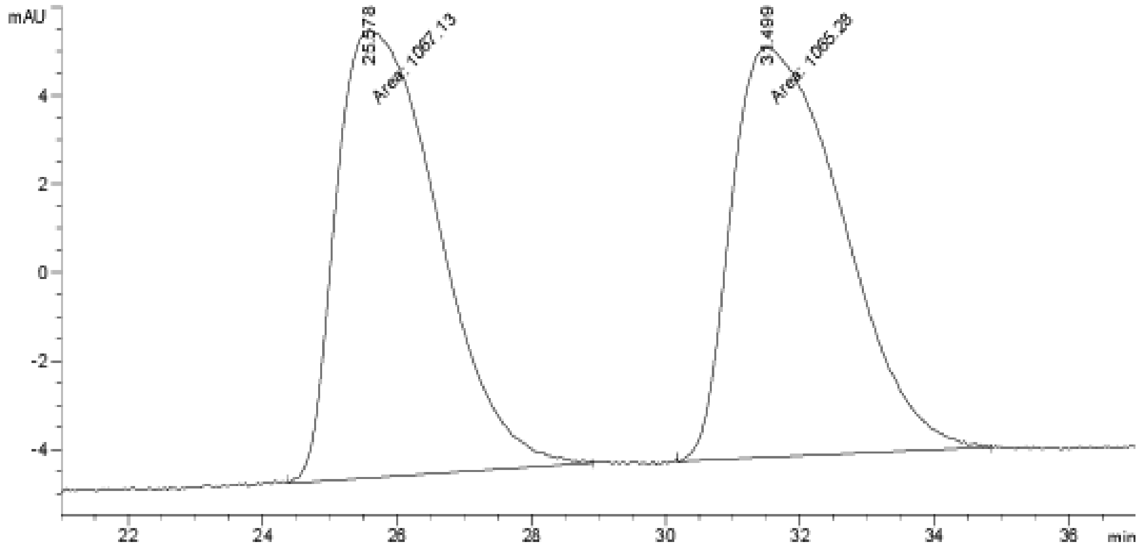

Peak #	RetTime [min]	Type	Width [min]	Area [mAU*s]	Height [mAU]	Area %
1	25.578	MM	1.7550	1067.12927	10.13421	50.0434
2	31.499	MM	1.9143	1065.27710	9.27456	49.9566

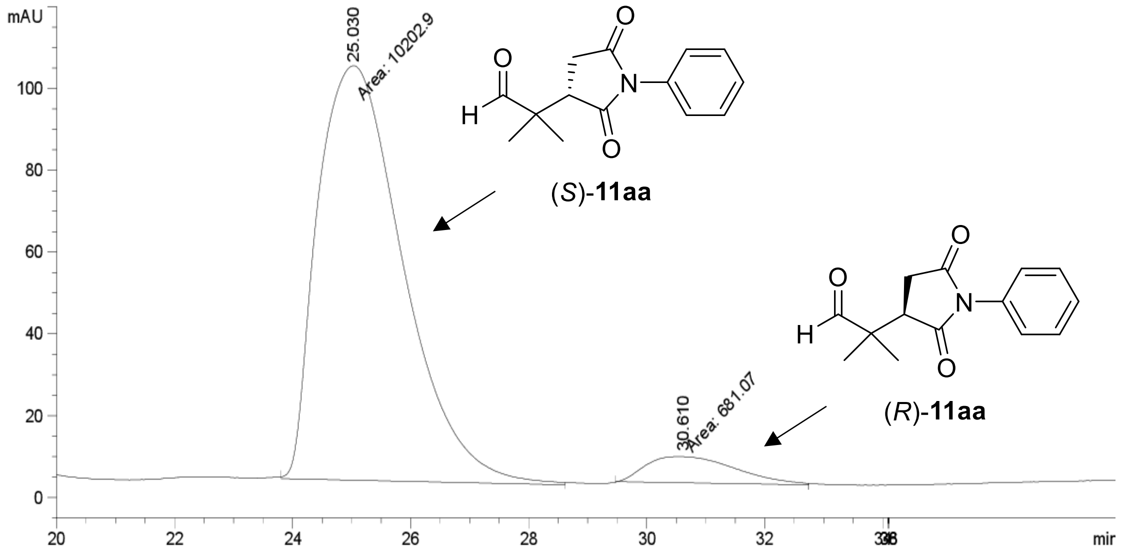

Peak #	RetTime [min]	Type	Width [min]	Area [mAU*s]	Height [mAU]	Area %
1	25.030	MM	1.6783	1.02029e4	101.32318	93.7425
2	30.610	MM	1.7671	681.07013	6.42370	6.2575

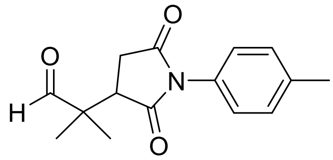

11ab

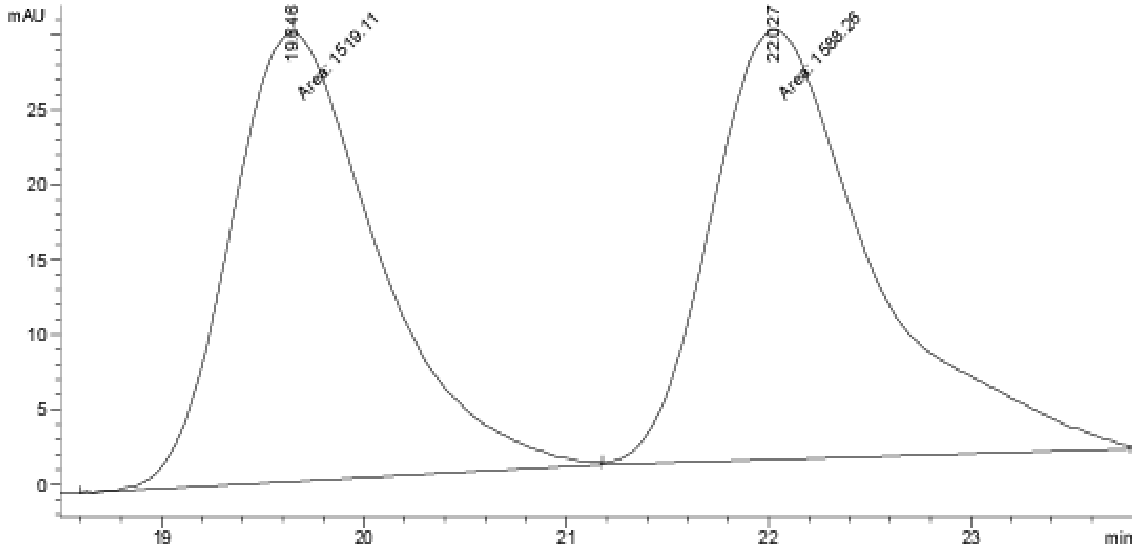

Peak #	RetTime [min]	Type	Width [min]	Area [mAU*s]	Height [mAU]	Area %
1	19.646	MM	0.8446	1519.10876	29.97655	48.8874
2	22.027	MM	0.9212	1588.25598	28.73413	51.1126

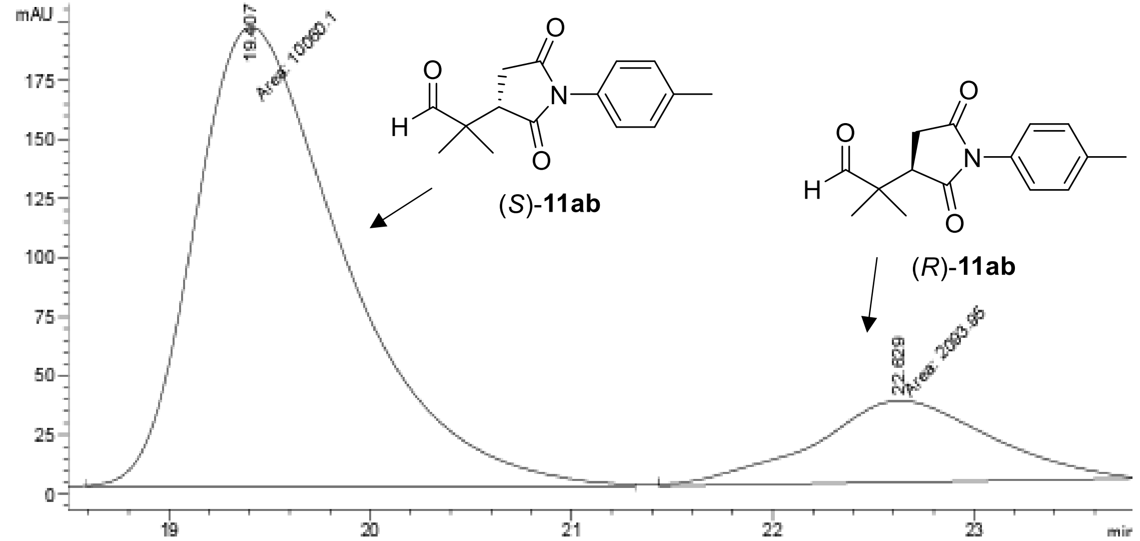

Peak #	RetTime [min]	Type	Width [min]	Area [mAU*s]	Height [mAU]	Area %
1	19.407	MM	0.8631	1.00601e4	194.25903	82.7716
2	22.629	MM	1.0115	2093.95093	34.50246	17.2284

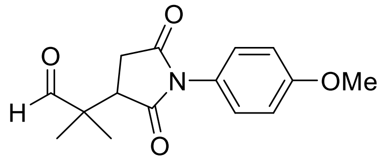

11ac

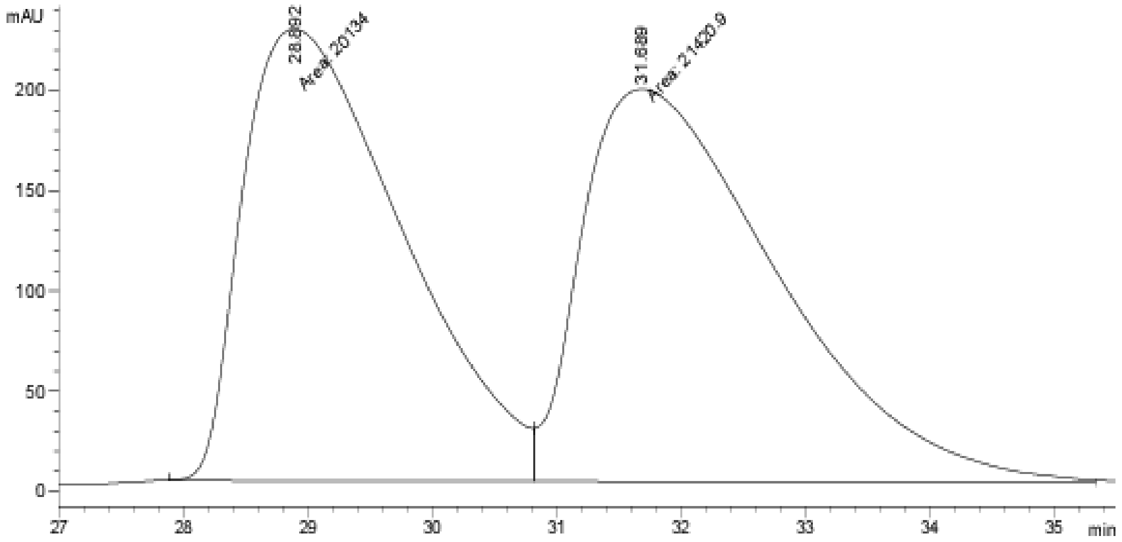

Peak #	RetTime [min]	Type	Width [min]	Area [mAU*s]	Height [mAU]	Area %
1	28.892	MF	1.4865	2.01340e4	225.74316	48.4515
2	31.689	FM	1.8220	2.14209e4	195.94800	51.5485

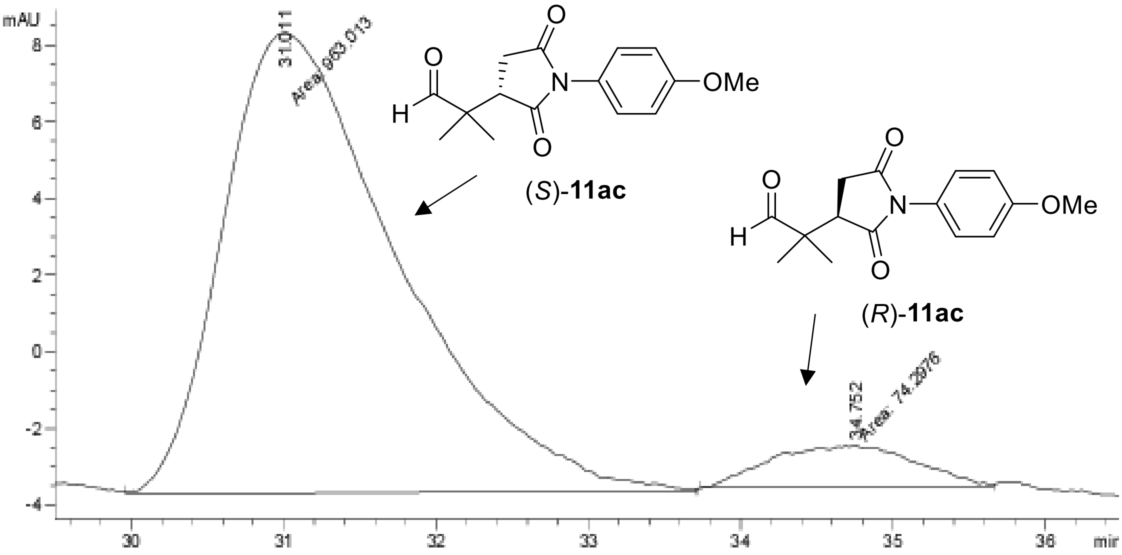

Peak #	RetTime [min]	Type	Width [min]	Area [mAU*s]	Height [mAU]	Area %
1	31.011	MM	1.3366	963.01294	12.00855	92.8375
2	34.752	MM	1.1159	74.29758	1.10971	7.1625

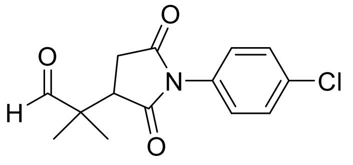

11ad

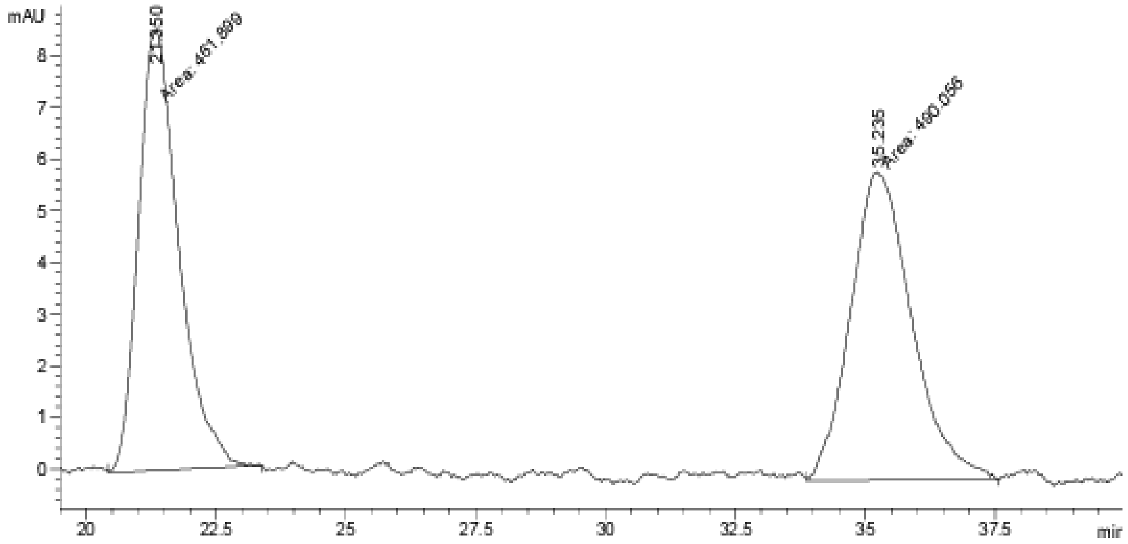

Peak #	RetTime [min]	Type	Width [min]	Area [mAU*s]	Height [mAU]	Area %
1	21.350	MM	0.9006	461.89896	8.54835	48.5211
2	35.235	MM	1.3697	490.05643	5.96305	51.4789

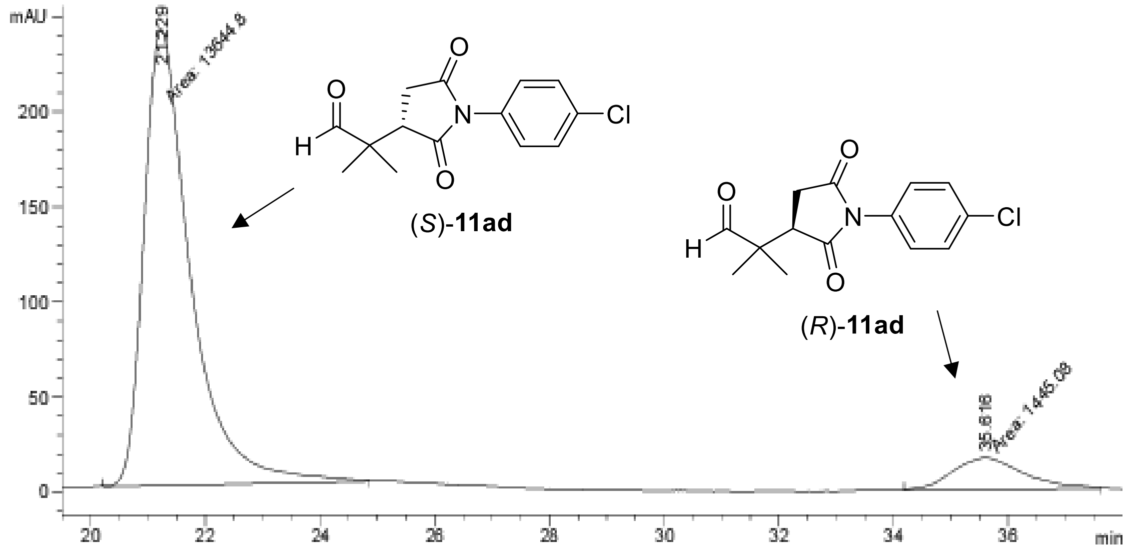

Peak #	RetTime [min]	Type	Width [min]	Area [mAU*s]	Height [mAU]	Area %
1	21.229	MM	0.9478	1.36448e4	239.92990	90.4235
2	35.616	MM	1.4437	1445.07654	16.68290	9.5765

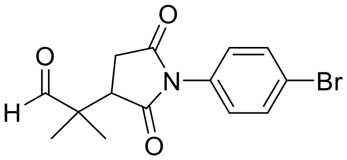

11ae

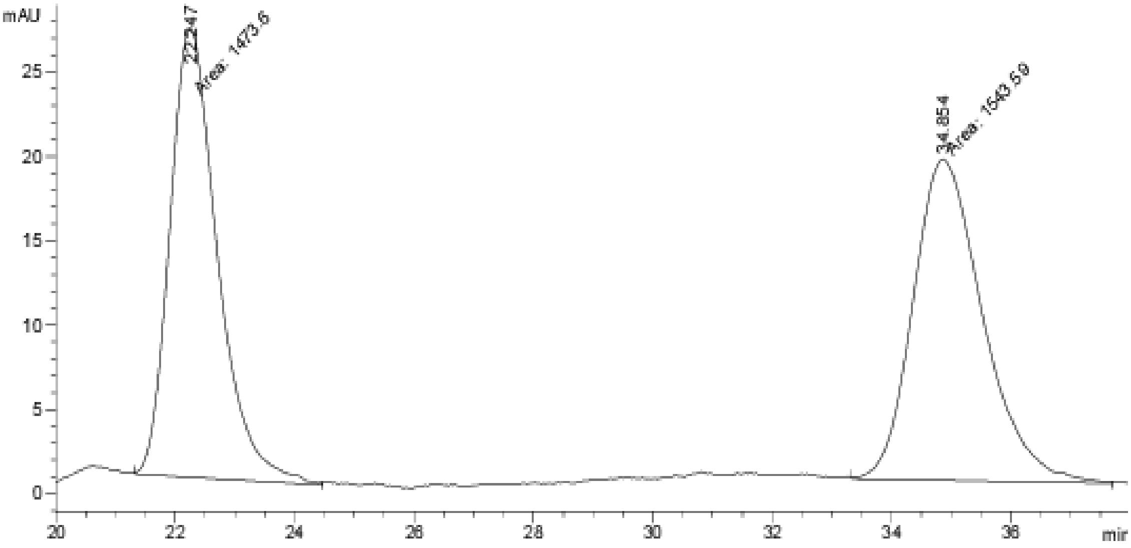

Peak #	RetTime [min]	Type	Width [min]	Area [mAU*s]	Height [mAU]	Area %
1	22.247	MM	0.9229	1473.59717	26.61119	48.8401
2	34.854	MM	1.3507	1543.58911	19.04656	51.1599

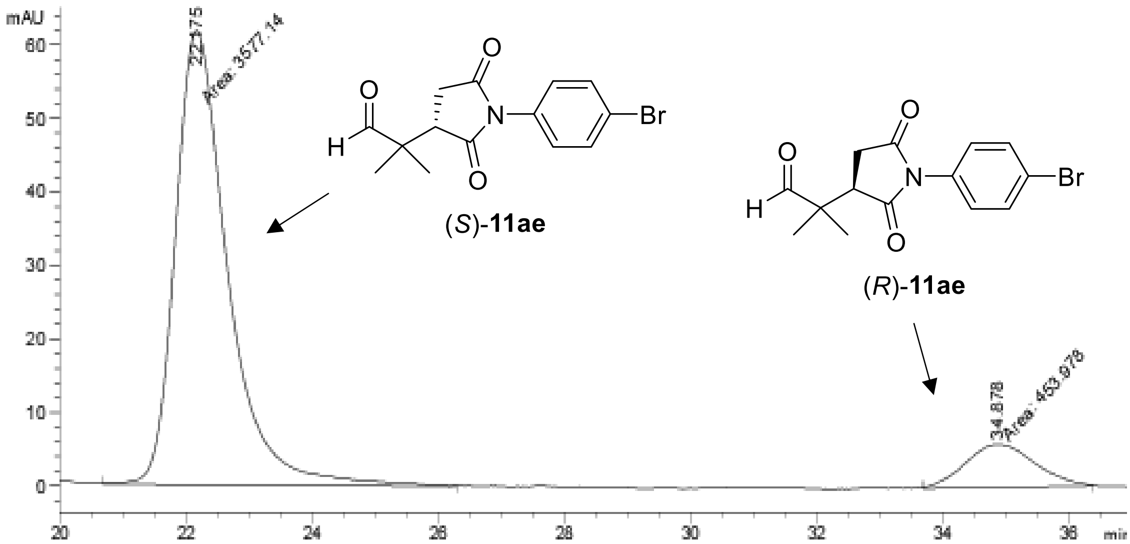

Peak #	RetTime [min]	Type	Width [min]	Area [mAU*s]	Height [mAU]	Area %
1	22.175	MM	0.9651	3577.13647	61.77628	88.7381
2	34.878	MM	1.2921	453.97833	5.85581	11.2619

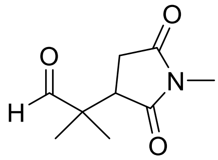

11af

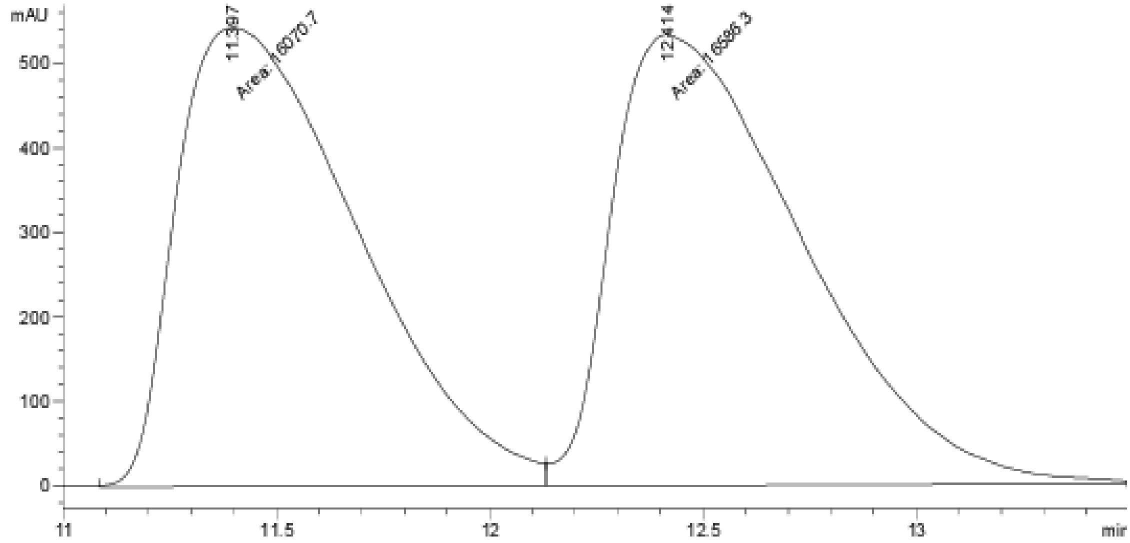

Peak #	RetTime [min]	Type	Width [min]	Area [mAU*s]	Height [mAU]	Area %
1	11.397	MF	0.4932	1.60707e4	543.09363	49.2105
2	12.414	FM	0.5181	1.65863e4	533.52704	50.7895

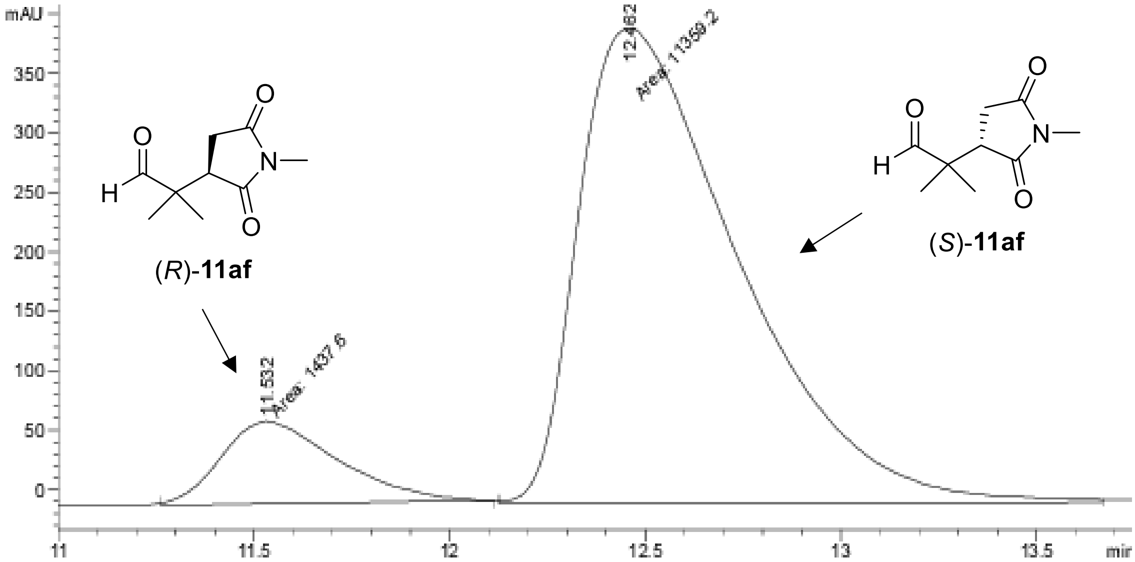

Peak #	RetTime [min]	Type	Width [min]	Area [mAU*s]	Height [mAU]	Area %
1	11.532	MM	0.3443	1437.60168	69.59547	11.2341
2	12.462	MM	0.4731	1.13592e4	400.18103	88.7659

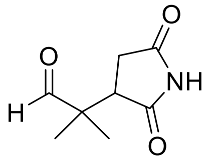

11ag

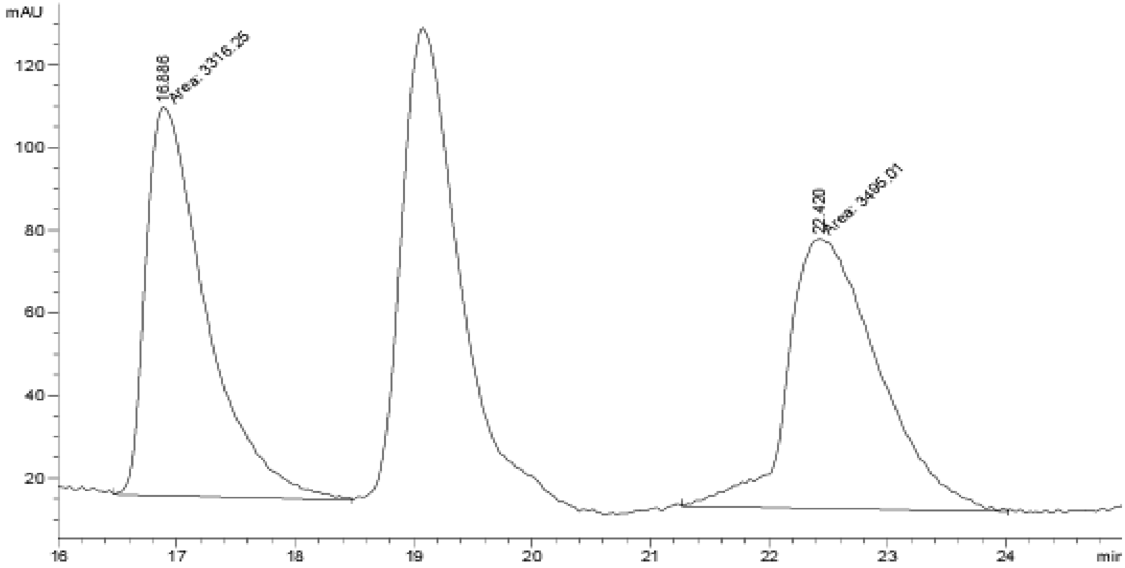

Peak #	RetTime [min]	Type	Width [min]	Area [mAU*s]	Height [mAU]	Area %
1	16.886	MM	0.5875	3316.25464	94.08321	48.6878
2	22.420	MM	0.8925	3495.01416	65.26379	51.3122

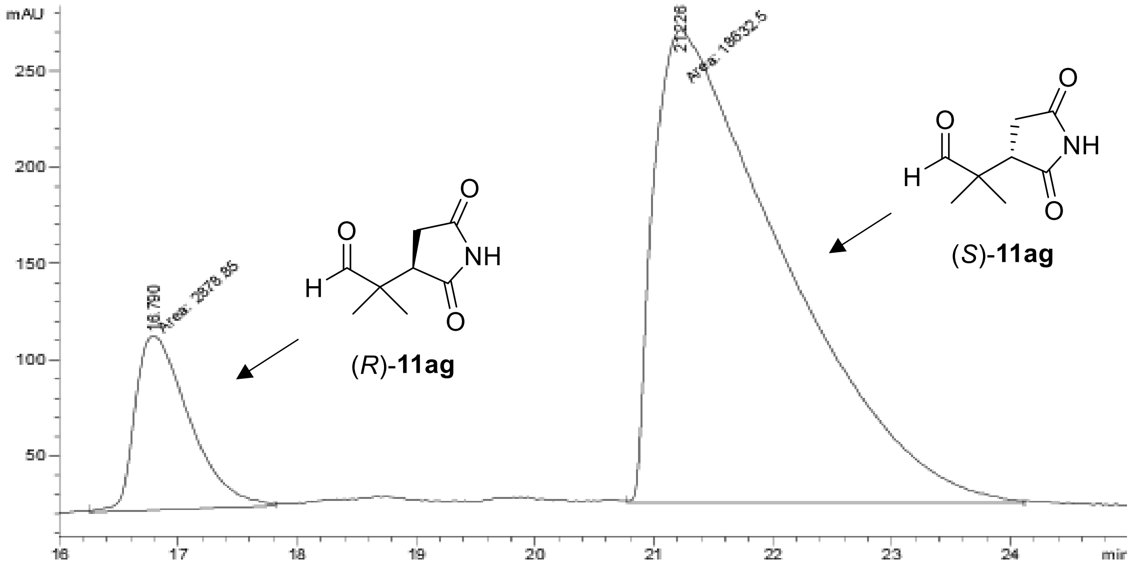

Peak #	RetTime [min]	Type	Width [min]	Area [mAU*s]	Height [mAU]	Area %
1	16.790	MM	0.5301	2878.84766	90.51169	13.3829
2	21.226	MM	1.2654	1.86325e4	245.40736	86.6171

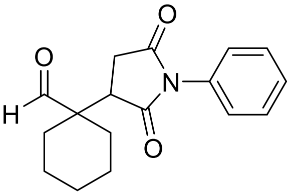

11ah

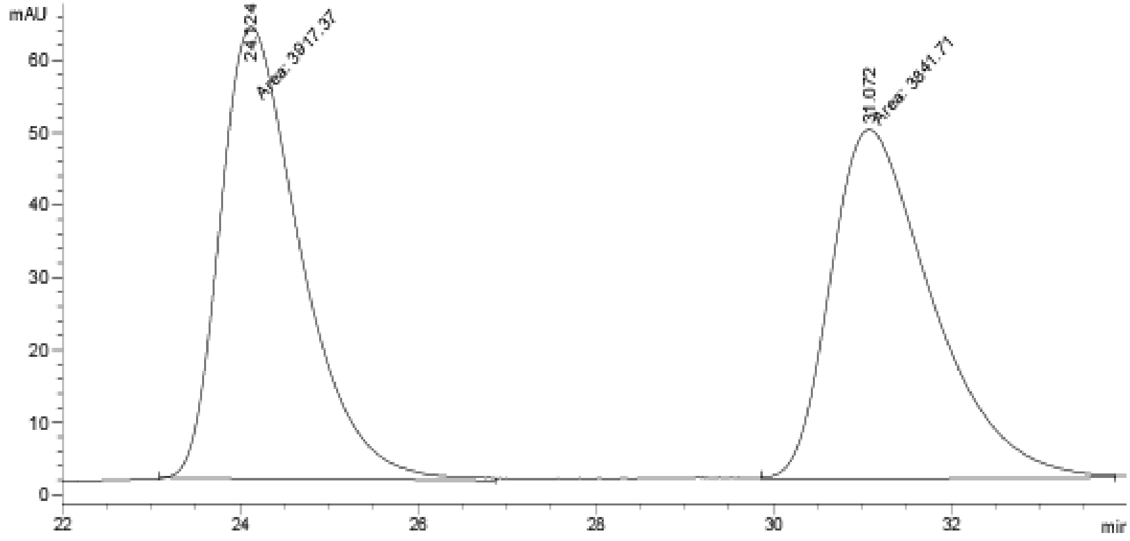

Peak #	RetTime [min]	Type	Width [min]	Area [mAU*s]	Height [mAU]	Area %
1	24.124	MM	1.0478	3917.36646	62.31222	50.4875
2	31.072	MM	1.3295	3841.71045	48.16110	49.5125

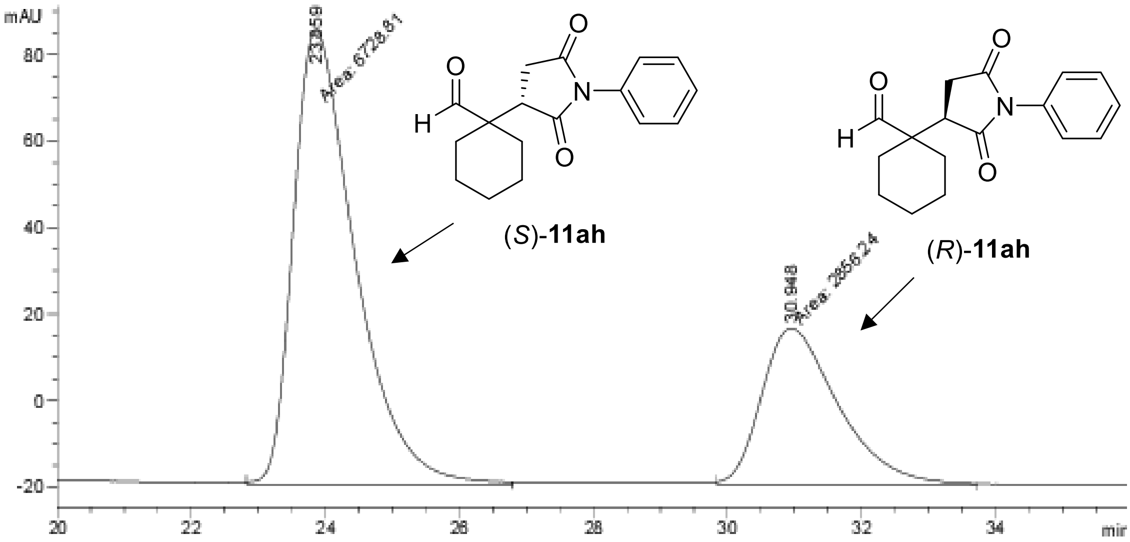

Peak #	RetTime [min]	Type	Width [min]	Area [mAU*s]	Height [mAU]	Area %
1	23.859	MM	1.0652	6728.80859	105.28285	70.2011
2	30.948	MM	1.3199	2856.24390	36.06693	29.7989

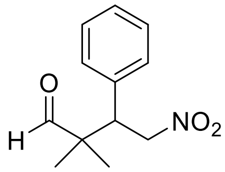

13aa

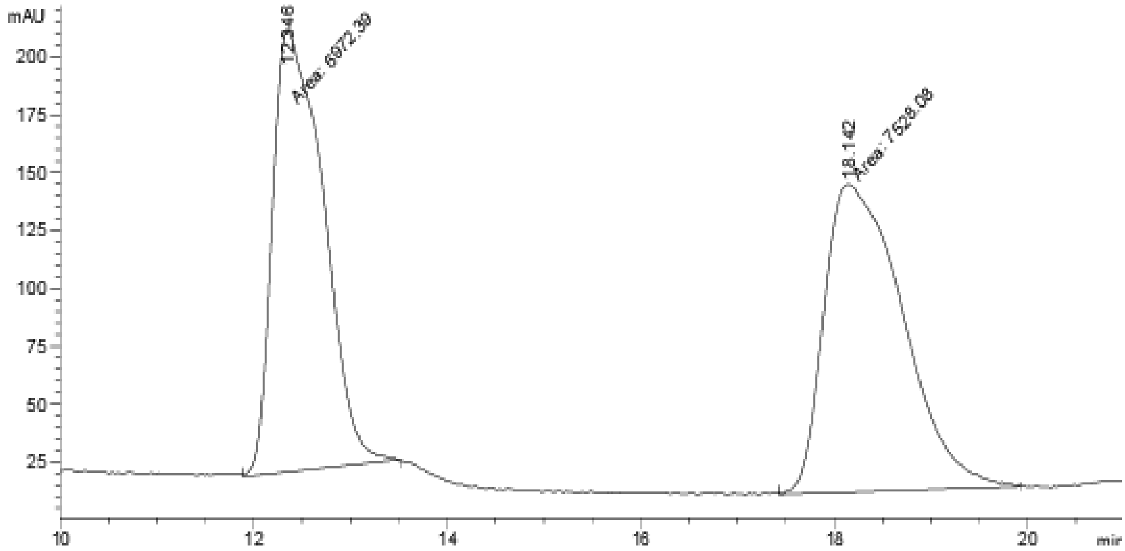

Peak #	RetTime [min]	Type	Width [min]	Area [mAU*s]	Height [mAU]	Area %
1	12.346	MM	0.6071	6972.39355	191.40536	48.0839
2	18.142	MM	0.9442	7528.07617	132.88312	51.9161

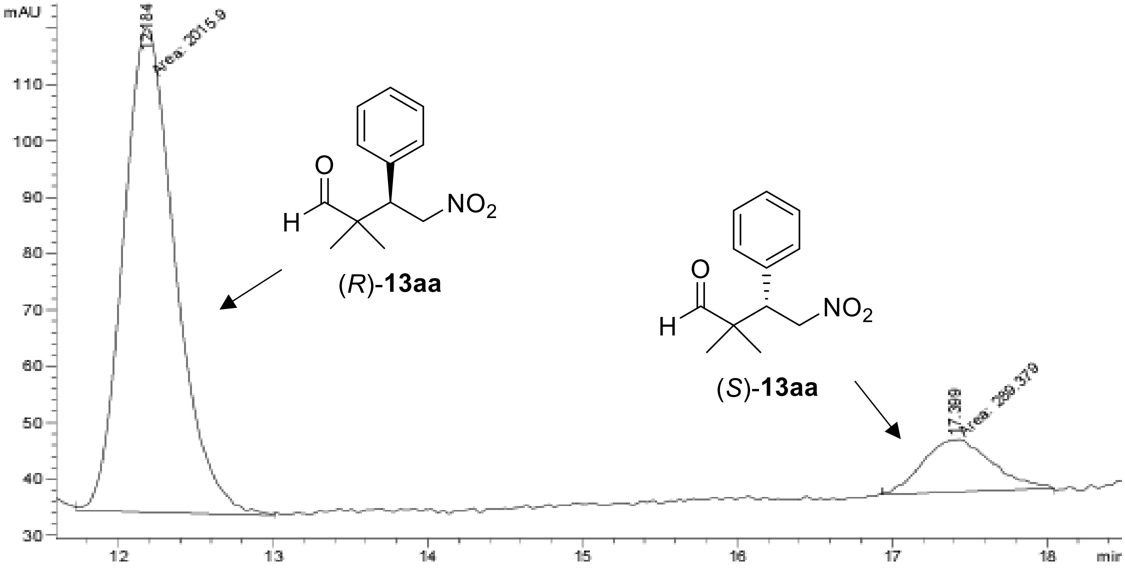

Peak #	RetTime [min]	Type	Width [min]	Area [mAU*s]	Height [mAU]	Area %
1	12.184	MM	0.3910	2015.90283	85.92458	87.4471
2	17.399	MM	0.5178	289.37946	9.31381	12.5529

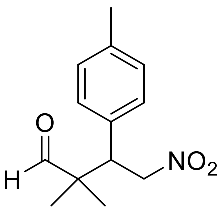

13ab

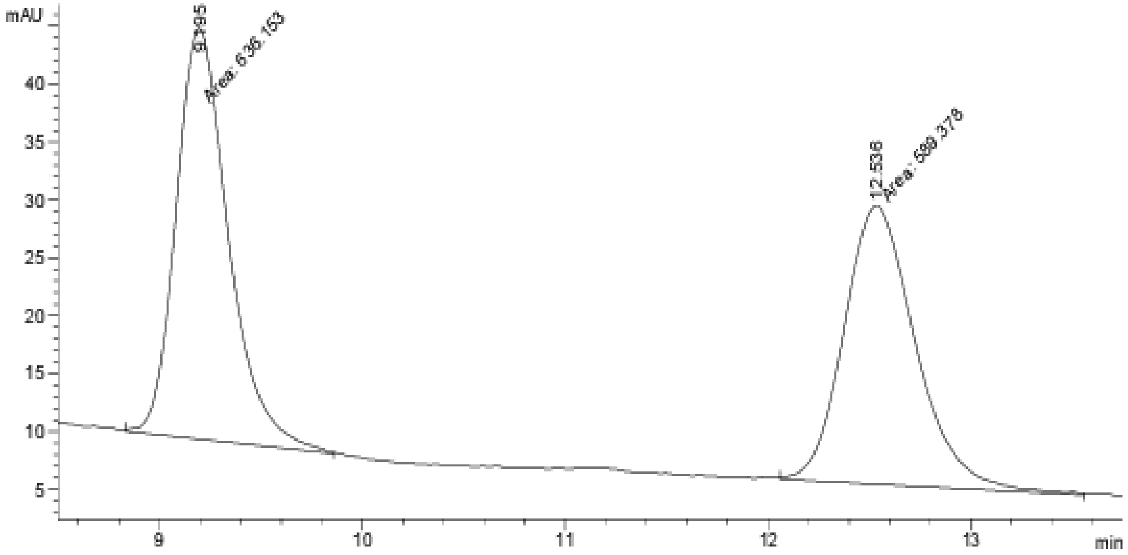

Peak #	RetTime [min]	Type	Width [min]	Area [mAU*s]	Height [mAU]	Area %
1	9.195	MM	0.2985	636.15277	35.51671	51.9083
2	12.536	MM	0.4080	589.37848	24.07382	48.0917

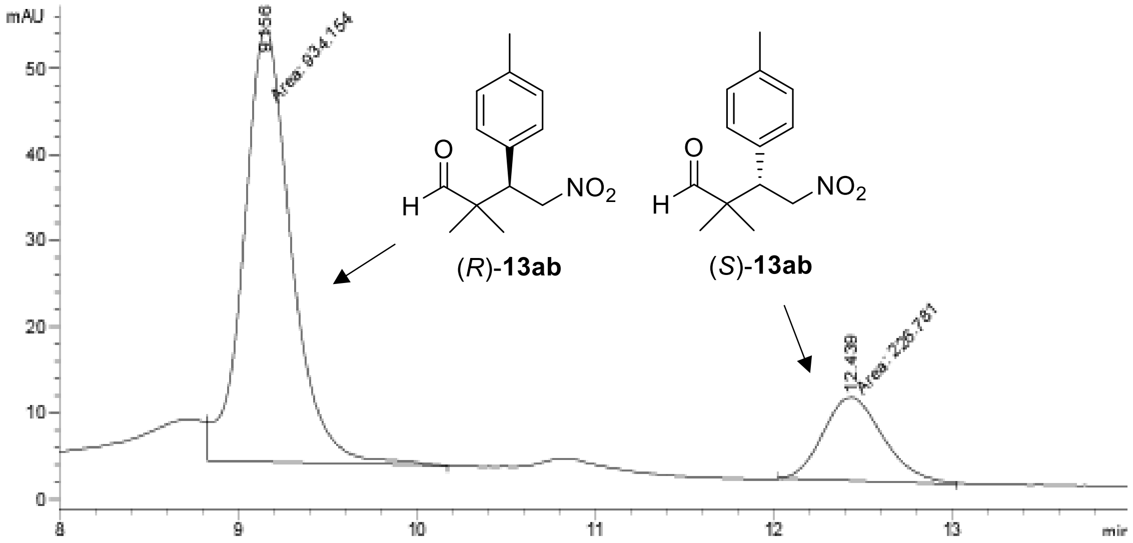

Peak #	RetTime [min]	Type	Width [min]	Area [mAU*s]	Height [mAU]	Area %
1	9.156	MM	0.3097	934.15424	50.26845	80.4656
2	12.439	MM	0.3924	226.78127	9.63192	19.5344

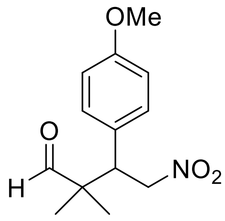

13ac

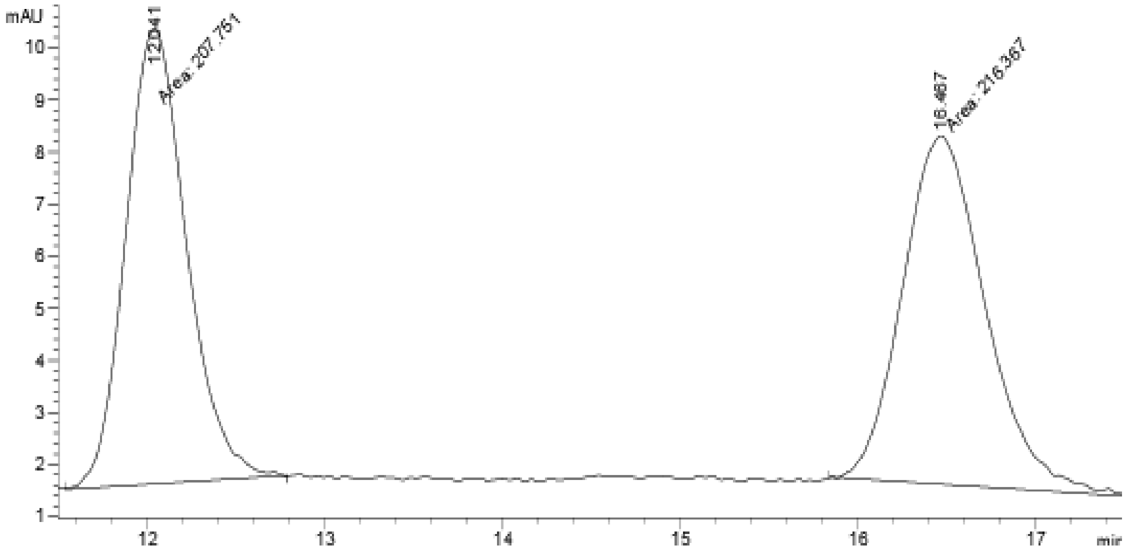

Peak #	RetTime [min]	Type	Width [min]	Area [mAU*s]	Height [mAU]	Area %
1	12.041	MM	0.3963	207.75137	8.73785	48.9842
2	16.467	MM	0.5384	216.36740	6.69733	51.0158

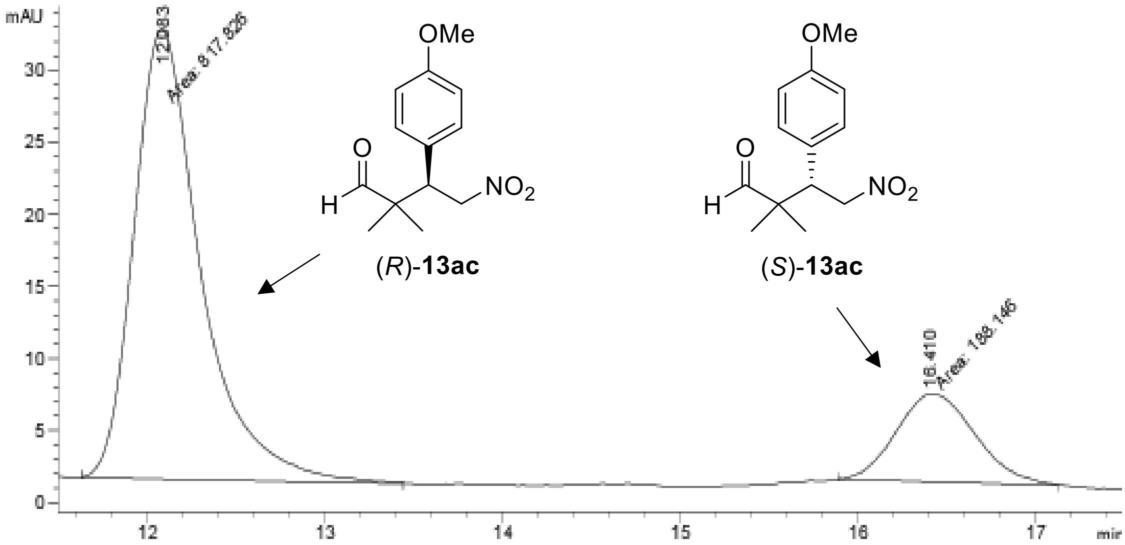

Peak #	RetTime [min]	Type	Width [min]	Area [mAU*s]	Height [mAU]	Area %
1	12.083	MM	0.4363	817.82568	31.24165	81.2971
2	16.410	MM	0.5130	188.14626	6.11217	18.7029

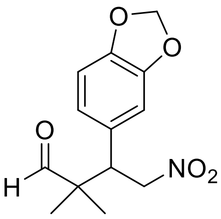

13ad

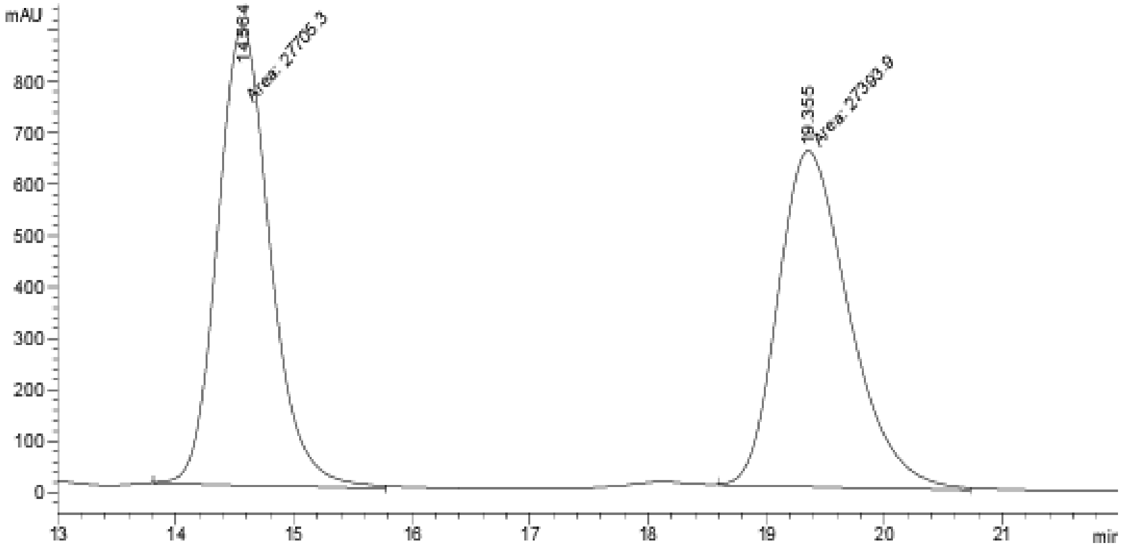

Peak #	RetTime [min]	Type	Width [min]	Area [mAU*s]	Height [mAU]	Area %
1	14.564	MM	0.5187	2.77053e4	890.18005	50.2826
2	19.355	MM	0.6989	2.73939e4	653.28668	49.7174

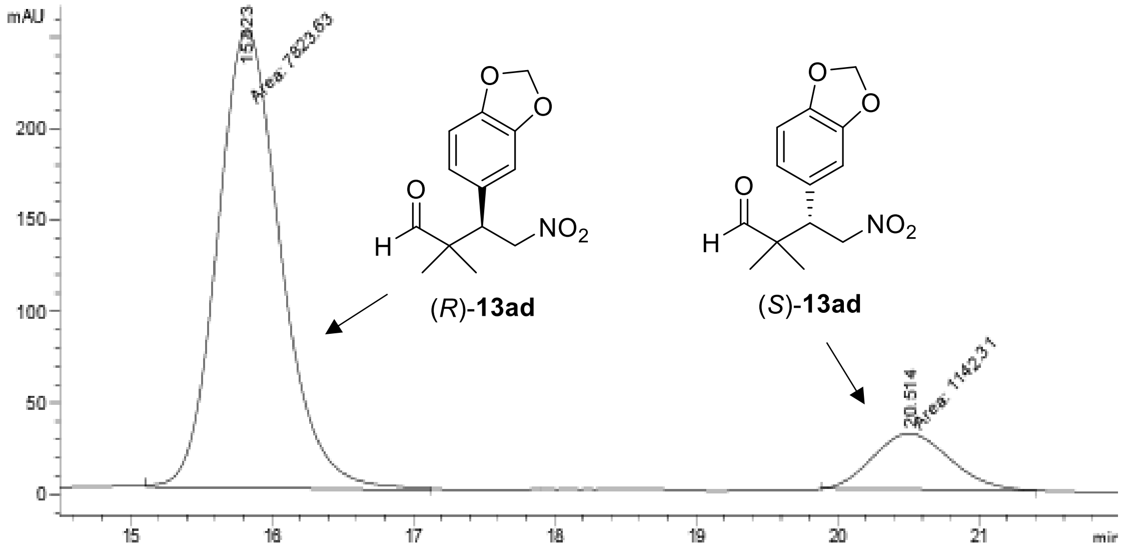

Peak #	RetTime [min]	Type	Width [min]	Area [mAU*s]	Height [mAU]	Area %
1	15.823	MM	0.5195	7823.63037	250.98103	87.2594
2	20.514	MM	0.6244	1142.31152	30.49207	12.7406

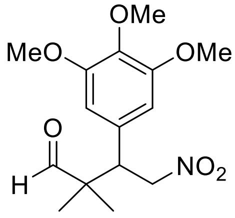

13ae

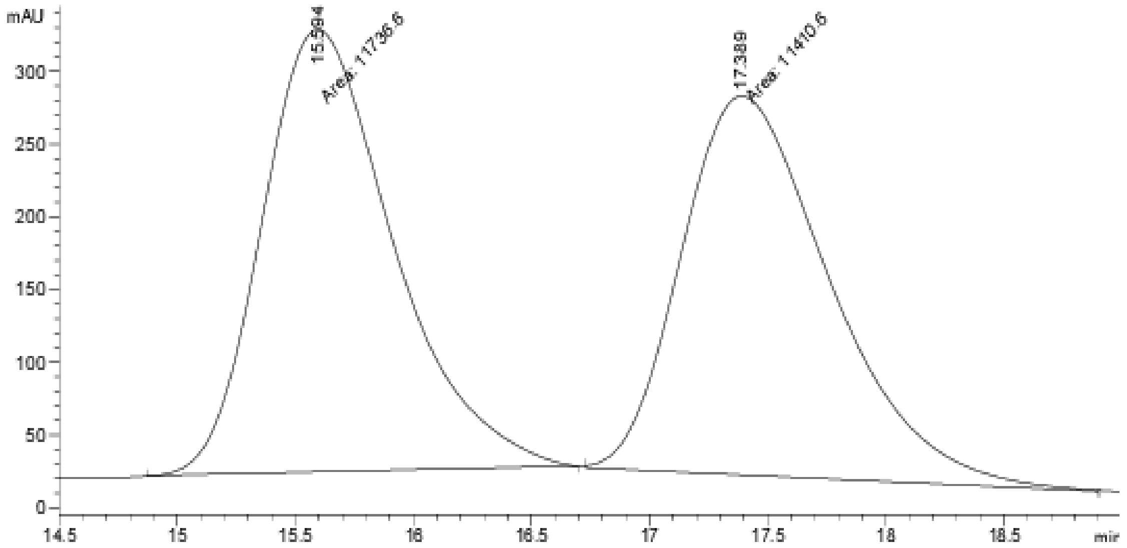

Peak #	RetTime [min]	Type	Width [min]	Area [mAU*s]	Height [mAU]	Area %
1	15.594	MM	0.6424	1.17366e4	304.50662	50.7041
2	17.389	MM	0.7301	1.14106e4	260.49799	49.2959

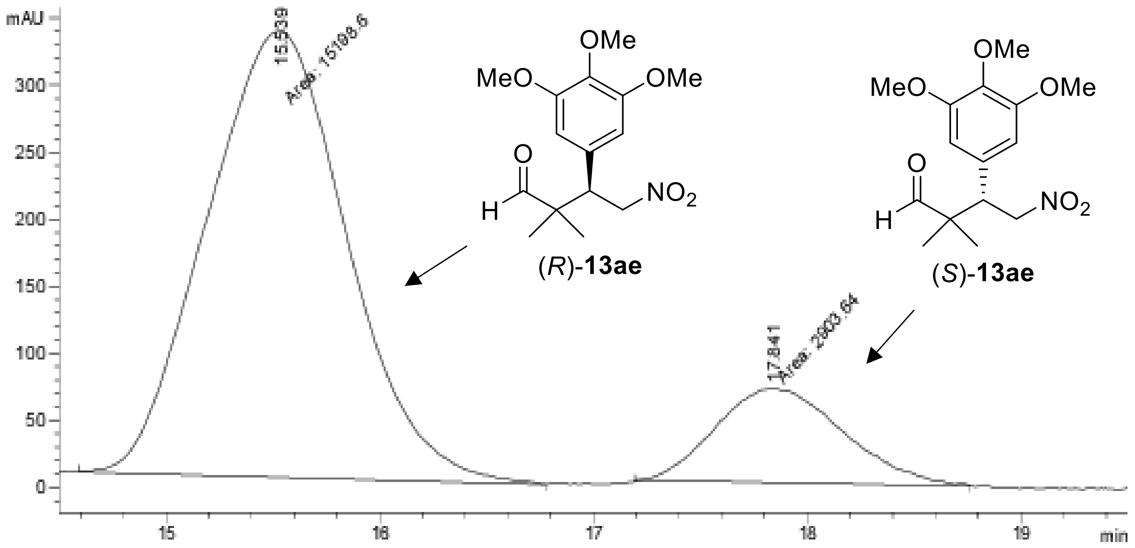

Peak #	RetTime [min]	Type	Width [min]	Area [mAU*s]	Height [mAU]	Area %
1	15.539	MM	0.7604	1.51986e4	333.12036	83.9598
2	17.841	MM	0.6869	2903.63989	70.45074	16.0402

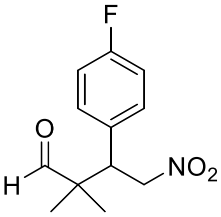

13af

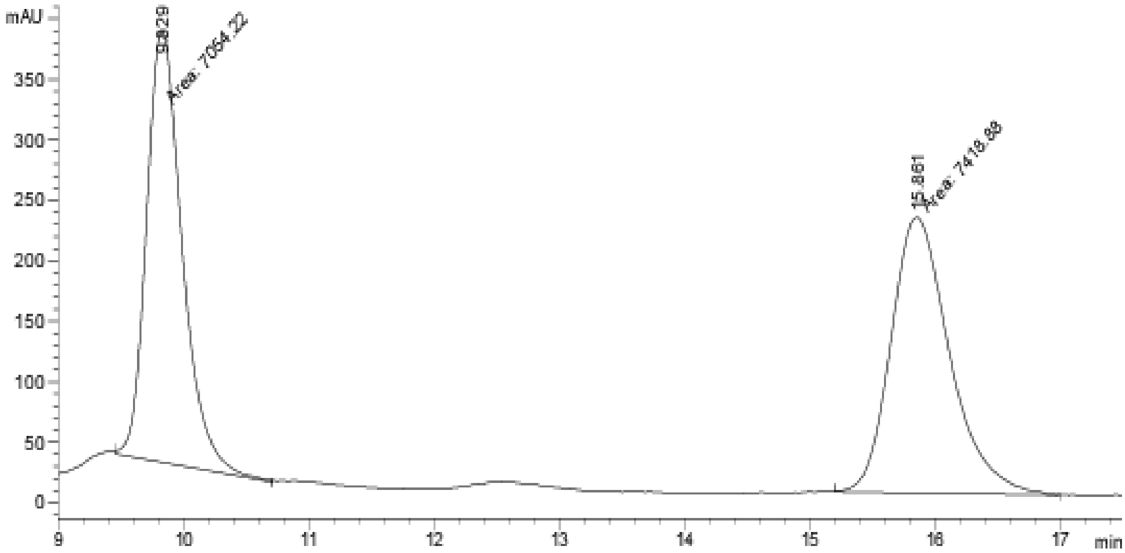

Peak #	RetTime [min]	Type	Width [min]	Area [mAU*s]	Height [mAU]	Area %
1	9.829	MM	0.3284	7054.22314	357.98685	48.7402
2	15.861	MM	0.5426	7418.88184	227.86906	51.2598

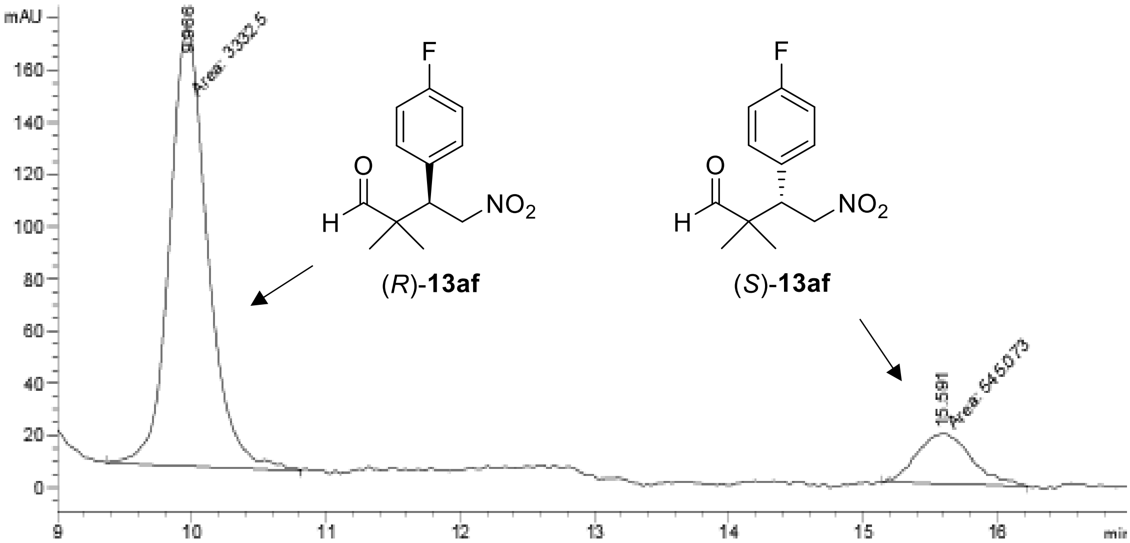

Peak #	RetTime [min]	Type	Width [min]	Area [mAU*s]	Height [mAU]	Area %
1	9.966	MM	0.3310	3332.50122	167.78302	85.9429
2	15.591	MM	0.4756	545.07300	19.10192	14.0571

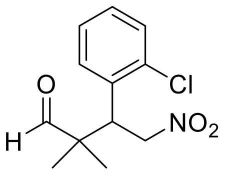

13ag

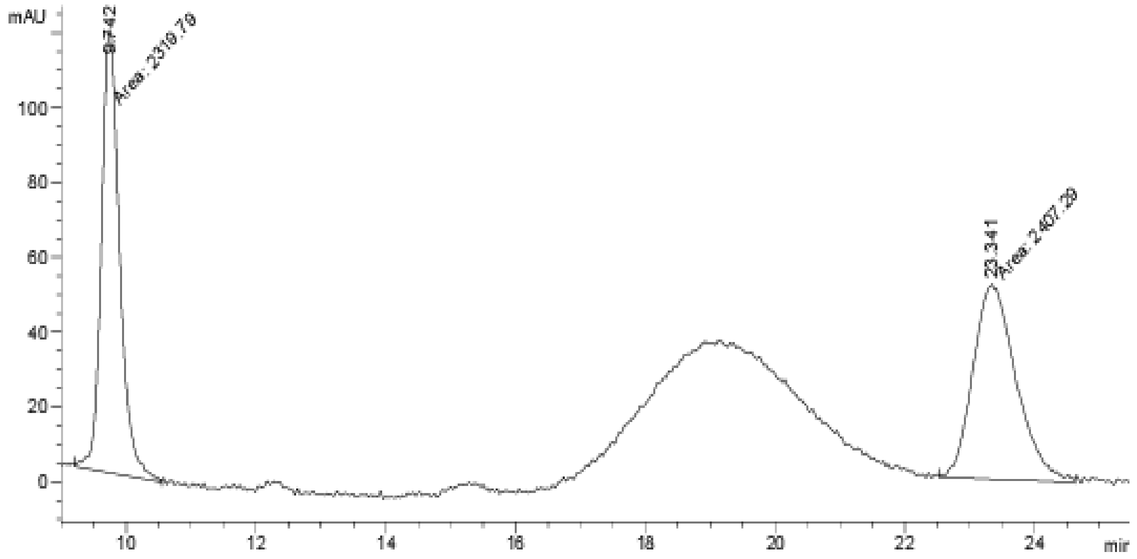

Peak #	RetTime [min]	Type	Width [min]	Area [mAU*s]	Height [mAU]	Area %
1	9.742	MM	0.3263	2319.79004	118.47284	49.0745
2	23.341	MM	0.7689	2407.28906	52.18210	50.9255

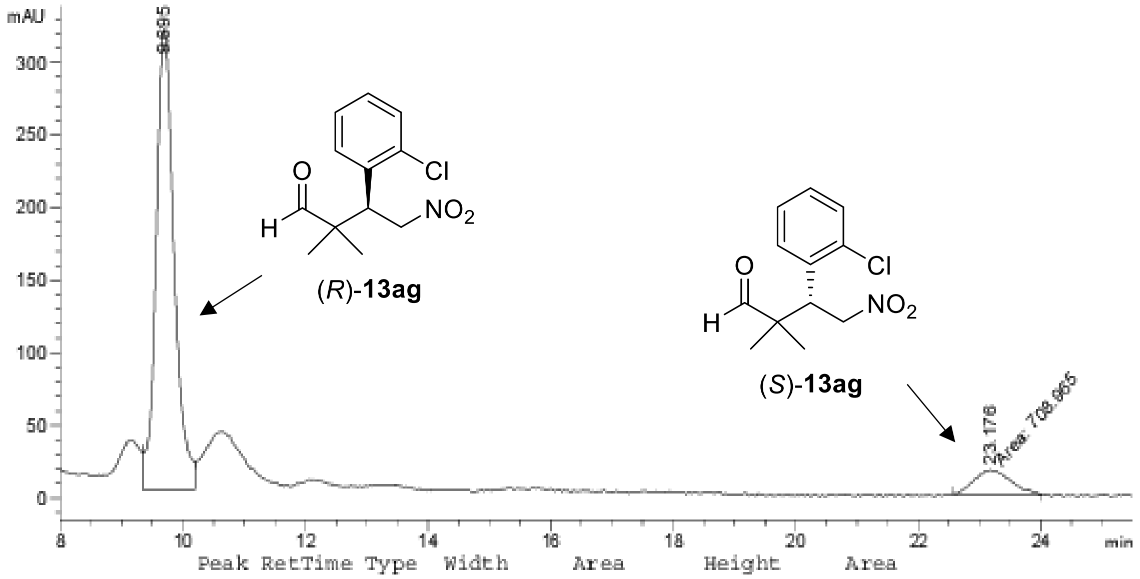

Peak #	RetTime [min]	Type	Width [min]	Area [mAU*s]	Height [mAU]	Area %
1	9.695	VV	0.3097	6467.21338	317.42947	90.1206
2	23.176	MM	0.6993	708.96527	16.89773	9.8794

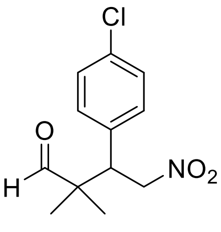

13ah

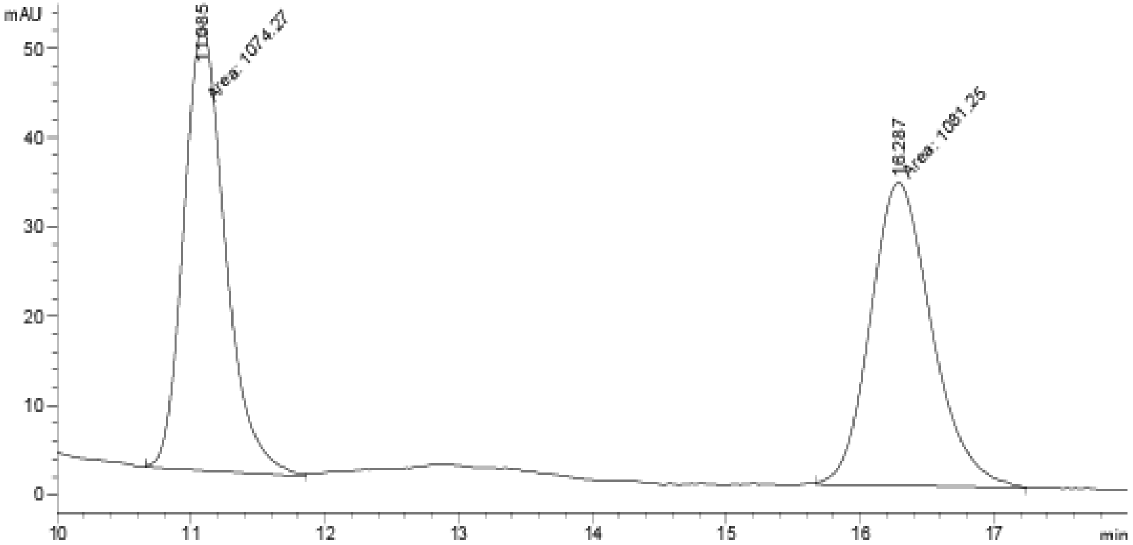

Peak #	RetTime [min]	Type	Width [min]	Area [mAU*s]	Height [mAU]	Area %
1	11.085	MM	0.3606	1074.26831	49.64988	49.8381
2	16.287	MM	0.5311	1081.24951	33.93406	50.1619

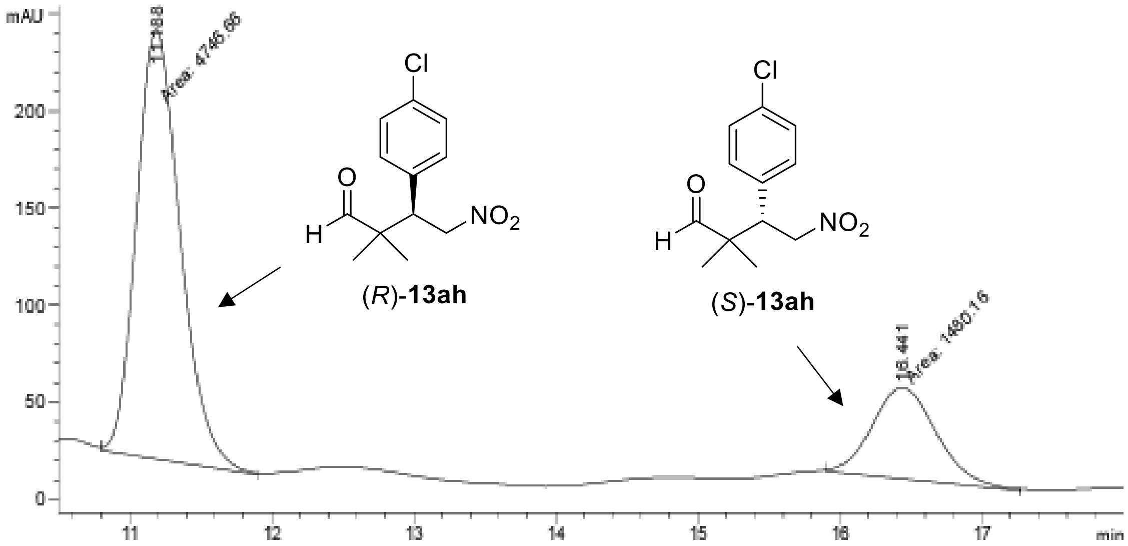

Peak #	RetTime [min]	Type	Width [min]	Area [mAU*s]	Height [mAU]	Area %
1	11.188	MM	0.3575	4746.65869	221.31865	76.2293
2	16.441	MM	0.5237	1480.15930	47.10495	23.7707

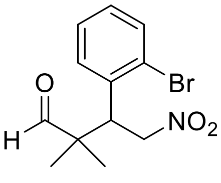

13ai

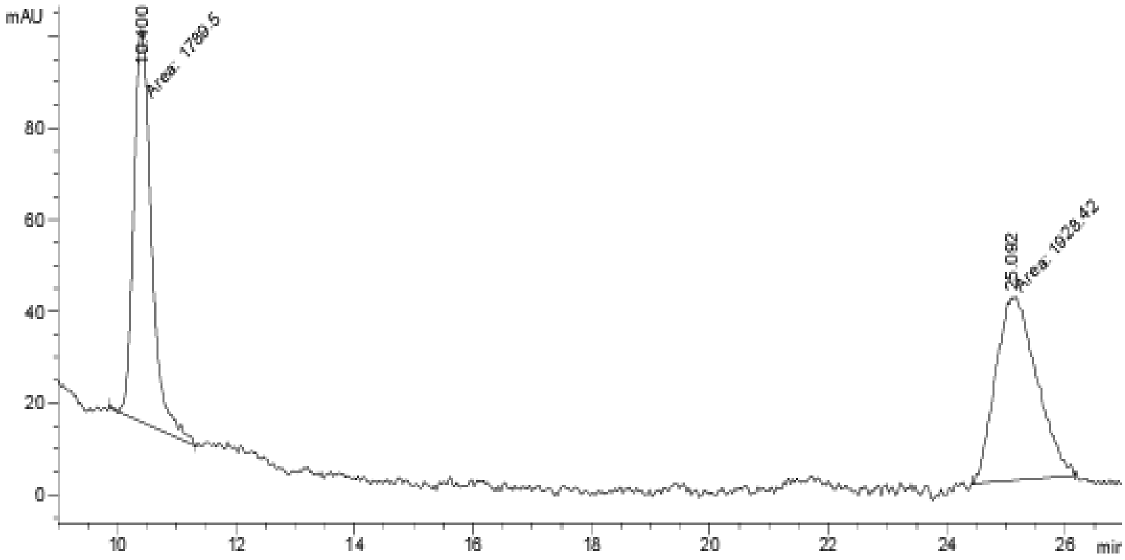

Peak #	RetTime [min]	Type	Width [min]	Area [mAU*s]	Height [mAU]	Area %
1	10.400	MM	0.3486	1789.49951	85.55537	48.1317
2	25.092	MM	0.8038	1928.42346	39.98392	51.8683

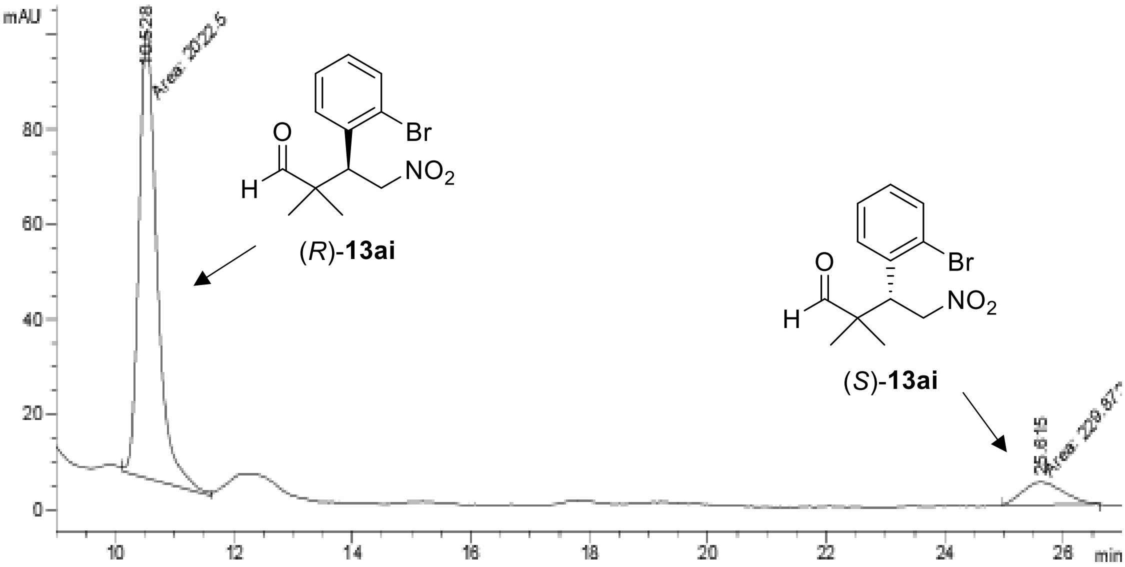

Peak #	RetTime [min]	Type	Width [min]	Area [mAU*s]	Height [mAU]	Area %
1	10.528	MM	0.3572	2022.50391	94.36970	89.7942
2	25.615	MM	0.7889	229.87239	4.85624	10.2058

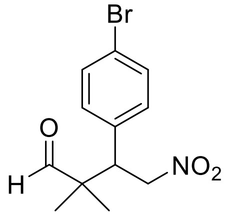

13aj

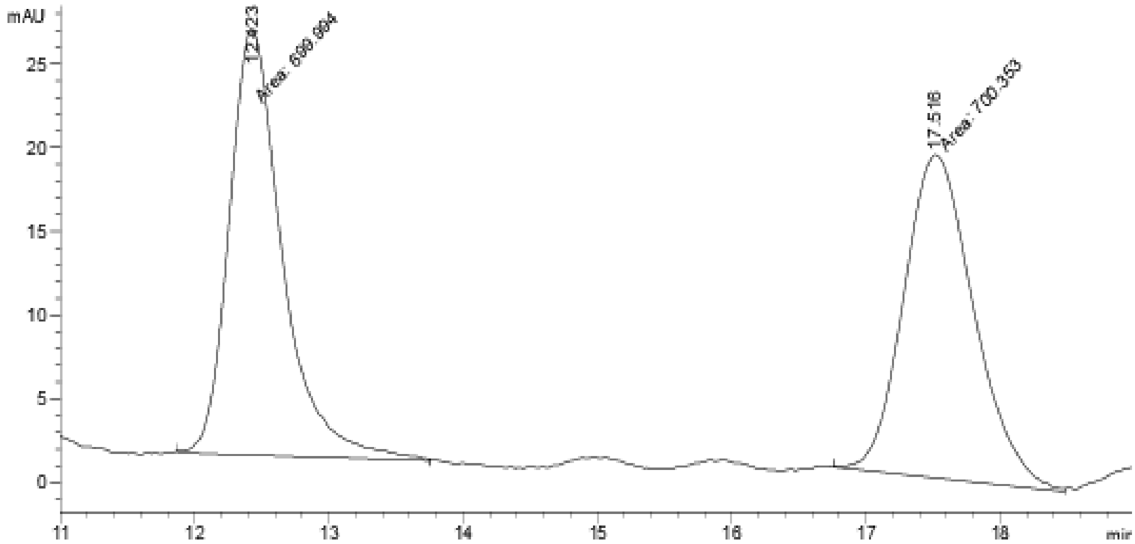

Peak #	RetTime [min]	Type	Width [min]	Area [mAU*s]	Height [mAU]	Area %
1	12.423	MM	0.4582	699.99438	25.46105	49.9872
2	17.516	MM	0.6048	700.35272	19.30123	50.0128

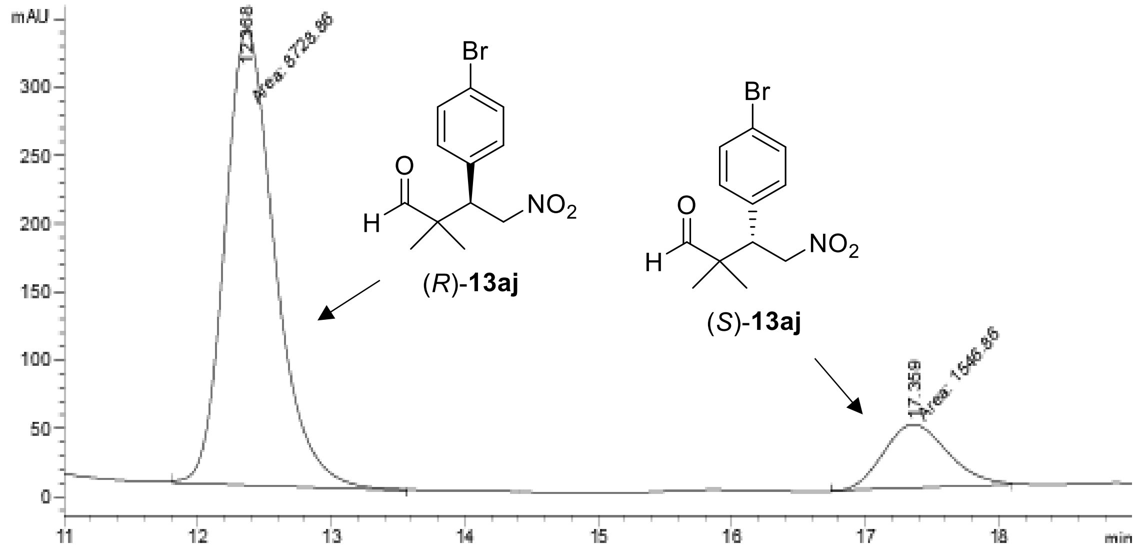

Peak #	RetTime [min]	Type	Width [min]	Area [mAU*s]	Height [mAU]	Area %
1	12.368	MM	0.4353	8728.85840	334.19949	84.9464
2	17.359	MM	0.5554	1546.86243	46.42253	15.0536

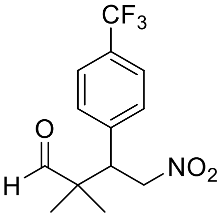

13ak

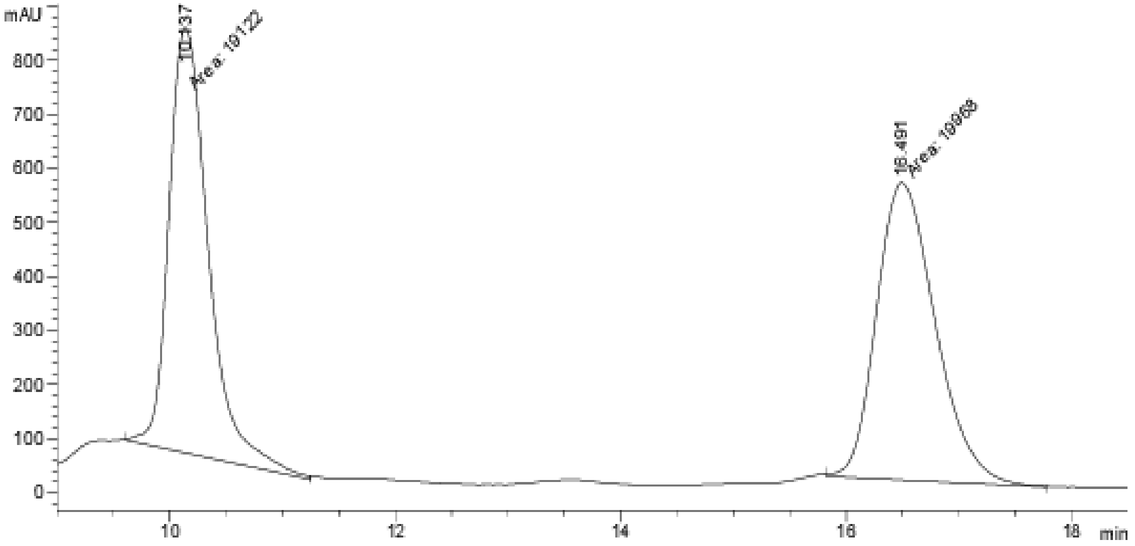

Peak #	RetTime [min]	Type	Width [min]	Area [mAU*s]	Height [mAU]	Area %
1	10.137	MM	0.4047	1.91220e4	787.52271	48.9179
2	16.491	MM	0.6043	1.99680e4	550.72736	51.0821

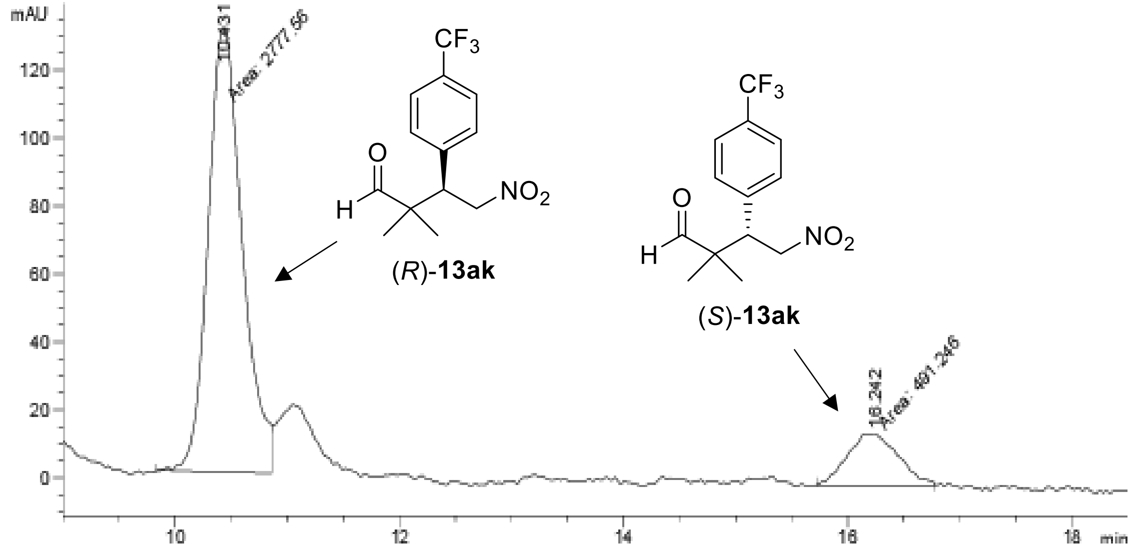

Peak #	RetTime [min]	Type	Width [min]	Area [mAU*s]	Height [mAU]	Area %
1	10.431	MF	0.3535	2777.55786	130.96844	84.9717
2	16.242	MM	0.5281	491.24628	15.50457	15.0283

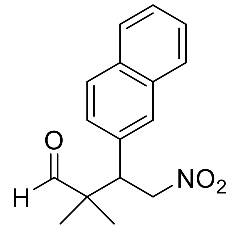

13al

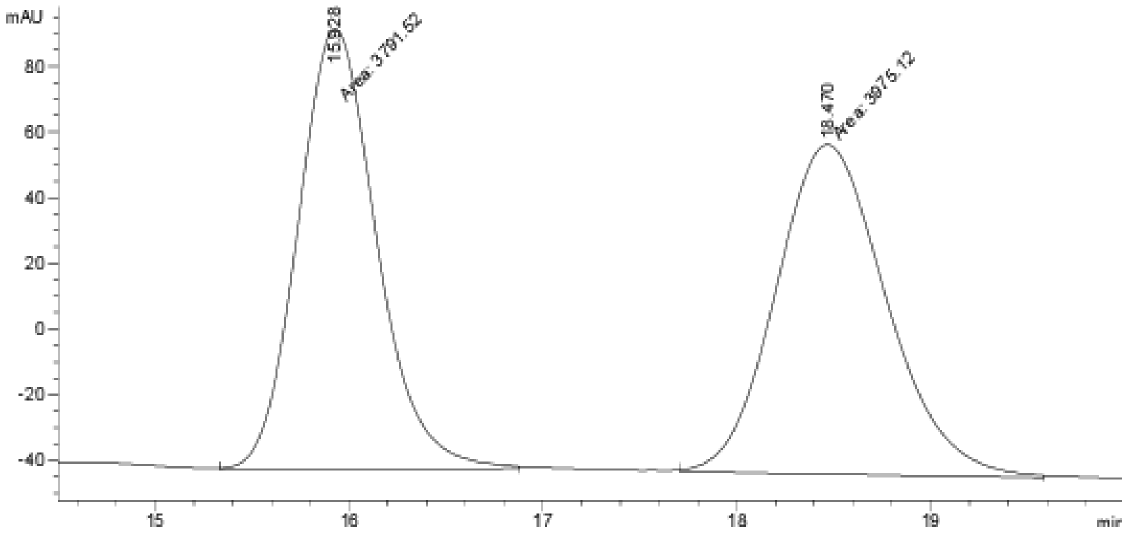

Peak #	RetTime [min]	Type	Width [min]	Area [mAU*s]	Height [mAU]	Area %
1	15.928	MM	0.4713	3791.51685	134.08318	48.8180
2	18.470	MM	0.6605	3975.12451	100.30030	51.1820

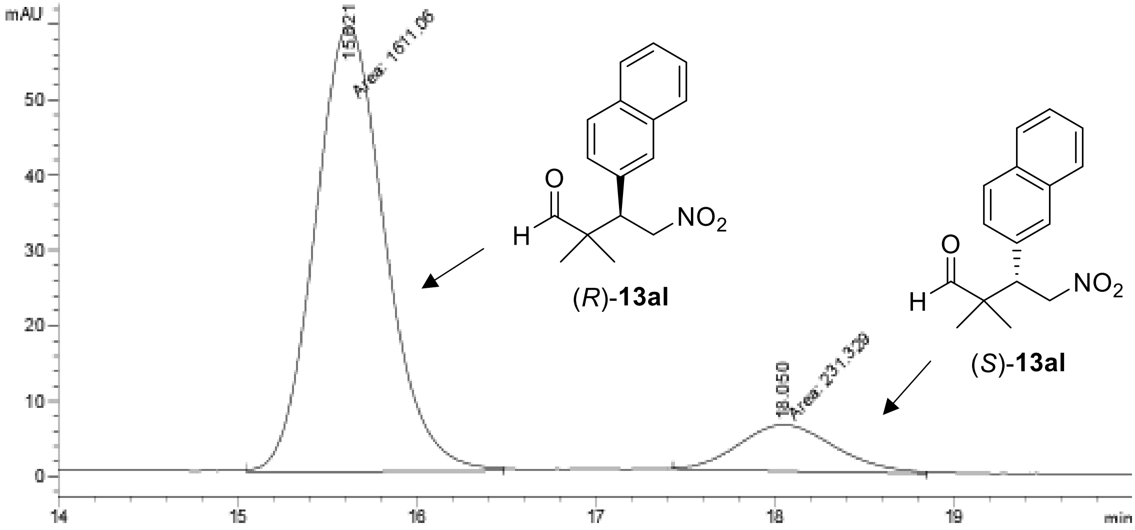

Peak #	RetTime [min]	Type	Width [min]	Area [mAU*s]	Height [mAU]	Area %
1	15.621	MM	0.4541	1611.06042	59.13071	87.4441
2	18.050	MM	0.6273	231.32935	6.14661	12.5559

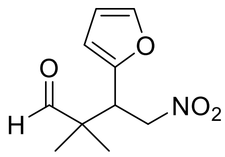

13am

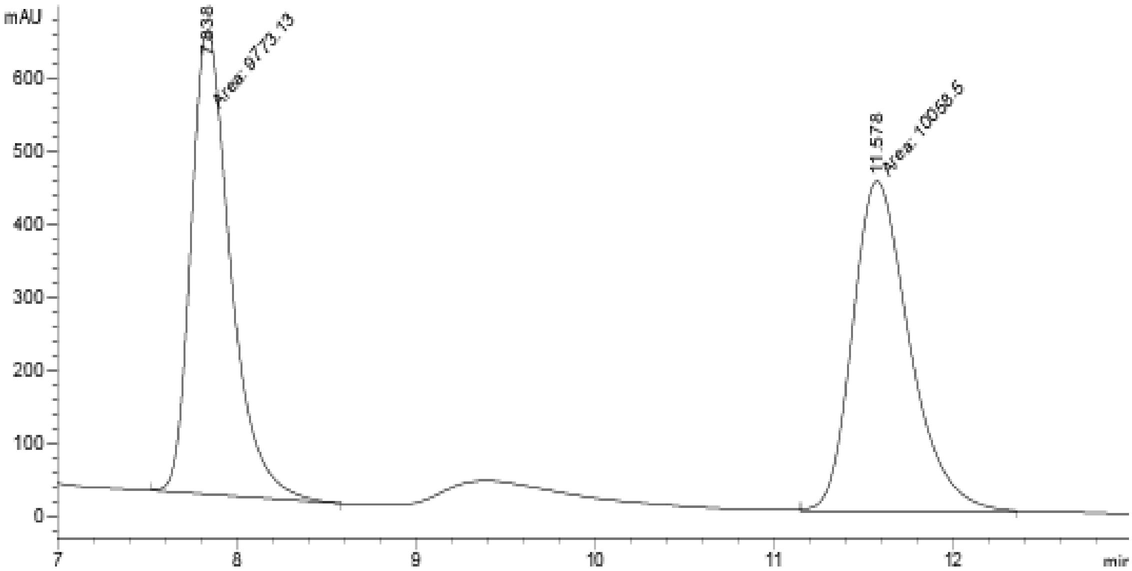

Peak #	RetTime [min]	Type	Width [min]	Area [mAU*s]	Height [mAU]	Area %
1	7.838	MM	0.2563	9773.12500	635.42133	49.2804
2	11.578	MM	0.3700	1.00585e4	453.11014	50.7196

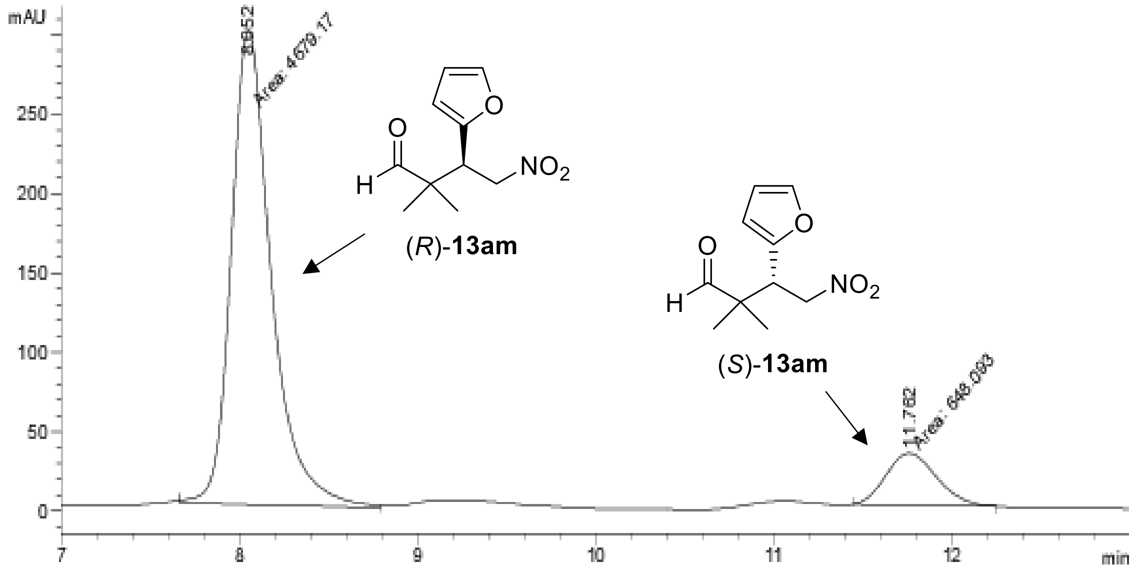

Peak #	RetTime [min]	Type	Width [min]	Area [mAU*s]	Height [mAU]	Area %
1	8.052	MM	0.2608	4679.16504	299.04559	87.8344
2	11.762	MM	0.3293	648.09320	32.80625	12.1656
